# Phylogenomic and molecular marker based studies to clarify the evolutionary relationships amongst Anoxybacillus species and demarcation of the family Anoxybacillaceae and some of its constituent genera

**DOI:** 10.1099/ijsem.0.006528

**Published:** 2024-09-17

**Authors:** Isha Patel, Sarah Bello, Radhey S. Gupta

**Affiliations:** 1 Department of Biochemistry and Biomedical Sciences, McMaster University, Hamilton, Ontario CA L8N 3Z5, Canada

**Keywords:** conserved signature indels (CSIs), molecular markers specific for the family Anoxybacillaceae and genera *Anoxybacillus*, *Geobacillus *and *Brevibacillus*, phylogenomic and comparative genomic analyses, Proposals for the novel genera *Anoxybacteroides*, *Paranoxybacillus* and *Thermaerobacillus*, *Brevibacillus*

## Abstract

The family *Anoxybacillaceae* was recently proposed encompassing the genera *Anoxybacillus*, *Geobacillus*, *Parageobacillus*, *Saccharococcus* and *Thermolongibacillus*. Of these genera, *Anoxybacillus* contains >50% of the *Anoxybacillaceae* species. However, *Anoxybacillus* species form multiple unrelated clades in phylogenetic trees and their evolutionary relationships are unclear. To clarify the evolutionary relationships of *Anoxybacillus* and other *Anoxybacillaceae* species, detailed phylogenomic and comparative analyses were conducted on 38 *Anoxybacillaceae* species with available genomes. In a phylogenomic tree based on 1148 core proteins, all *Anoxybacillus*, *Geobacillus*, *Parageobacillus*, *Saccharococcus* and *Thermolongibacillus* species, excepting *Anoxybacillus sediminis*, formed a strongly supported clade representing the family *Anoxybacillaceae*. Five conserved signature indels (CSIs) reported here are also uniquely found in these species, providing robust means for the demarcation of family *Anoxybacillaceae* in molecular terms. In our phylogenomic tree and in the Genomic Taxonomy Database, *Anoxybacillus* species formed four distinct clades designated as *Anoxybacillus sensu stricto* (containing the type species *A. pushchinoensis*), *Anoxybacillus*_A, *Anoxybacillus*_B and *Anoxybacillus*_C. Our analyses have identified 17 novel CSIs which offer means to reliably distinguish species from these clades based upon multiple uniquely shared molecular characteristics. Additionally, we have identified three and seven CSIs specific for the genera *Geobacillus* and *Brevibacillus*, respectively. All seven *Brevibacillus*-specific CSIs are also shared by *Anoxybacillus sediminis*, which branches reliably with this genus. Based on the strong phylogenetic and molecular evidence presented here, we are proposing that the genus *Anoxybacillus* should be restricted to only the species from *Anoxybacillus sensu stricto* clade, whereas the species from *Anoxybacillus*_A, *Anoxybacillus*_B, and *Anoxybacillus*_C clades should be transferred into three novel genera *Anoxybacteroides* gen. nov., *Paranoxybacillus* gen. nov. and *Thermaerobacillus* gen. nov., respectively. Additionally, we are also proposing the transfer of *Anoxybacillus sediminis* to the genus *Brevibacillus*. The proposed changes, which reliably depict the evolutionary relationships among *Anoxybacillaceae* species, should be helpful in the studies of these organisms.

## Data availability

All supplementary data files associated with this manuscript are available at the following link. https://doi.org/10.6084/m9.figshare.26485276.v1https://doi.org/10.6084/m9.figshare.26485276[1]

## Introduction

The genus *Anoxybacillus* was proposed by Pikuta *et al.* [2] and it initially consisted of only two species, *A. flavithermus* and *A. pushchinoensis* (type species). Since then, the number of validly published species in this genus has grown to 23 [3,4]. These newer species have been assigned to this genus, or transferred into it from other genera, based primarily on phenotypic characteristics and 16S rRNA or 16S rDNA gene-based phylogeny and sequence similarity studies [2,5]. *Anoxybacillus* species generally show Gram-positive staining and they are spore-forming facultative anaerobic bacteria [4,6]. These species have been isolated from various extreme environments such as geothermal soils, hot springs, manure, and mud samples [2,4, 7,13]. Due to their generally alkaliphilic and thermophilic properties, several species from this genus (viz. *A. amylolyticus*, *A. flavithermus*, *A. kamchatkensis* and *A. pushchinoensis*), are currently used in industrial applications, including biotechnology, biodegradation, and bioremediation processes [7,10, 14,16].

Recently, Chuvochina *et al.* [17,18] in their comprehensive proposal on defining the higher taxonomic ranks of prokaryotic organisms, based on relative evolutionary divergence in their Genome Taxonomic Database (GTDB) tree [18], have transferred the genus *Anoxybacillus* along with the genera *Geobacillus* [19], *Parageobacillus* [20,21], *Saccharococcus* [22] and *Thermolongibacillus* [23] into a new family, *Anoxybacillaceae*. Like the *Anoxybacillus* species, species from these other genera are also Gram-stain-positive, spore-forming, facultative anaerobic bacteria, which have generally been isolated thermophilic environments [24,25]. A close relationship among the species of these genera is also seen in earlier phylogenetic studies based on 16S rRNA/rDNA sequences [5,7, 12, 23, 26, 27]. However, the placement of these genera into the family *Anoxybacillaceae* is primarily based on their clustering in phylogenetic trees. No characteristic is known at present that is specifically shared by the members of this family. Another unresolved aspect of this family concerns the genus *Anoxybacillus*, which comprises >50% of the *Anoxybacillaceae* species. Earlier phylogenetic studies based on the 16S rRNA and *rpoB* gene sequences indicate that *Anoxybacillus* species form multiple distinct clades, which are separated from each other by long branches, suggesting that they are evolutionarily distantly related to each other [4,10,12, 28]. In some studies, which included species from closely related genera, viz. *Geobacillus* and *Saccharococcus*, *Anoxybacillus* species showed polyphyletic branching with the species from these genera, indicating that they do not form a monophyletic lineage [13,29]. In the GTDB taxonomy, which provides an important resource for understanding prokaryotic taxonomy [18], *Anoxybacillus* species are placed into four genus-level groupings corresponding to some of the clades observed in earlier studies [4,11,13, 18, 28, 29, 31]. These observations indicate the need for undertaking detailed phylogenomic and comparative genomic studies on *Anoxybacillaceae* species to reliably delineate their evolutionary relationships and demarcate different genus-level clades of *Anoxybacillus* species.

Genome sequences are now available for 38 of the 44 validly *Anoxybacillaceae* species [3] in the NCBI database (https://www.ncbi.nlm.nih.gov/genome/) [32]. Using these genome sequences, we report here a comprehensive examination of the evolutionary relationships among *Anoxybacillaceae* species using multiple independent approaches. These approaches include, (i) reconstruction of a phylogenomic tree based on 1148 core proteins from the genomes of these species; (ii) reconstruction of a 16S rRNA gene tree for the type strains of all *Anoxybacillaceae* species; (iii) determination of pairwise average amino acid identity (AAI) between these species; (iv) detailed analyses of protein sequences from *Anoxybacillaceae* species to identify molecular markers consisting of conserved signature indels (CSIs), that are specifically present in species from different strongly supported clades. The results from these studies provide a reliable framework for elucidating the evolutionary relationships amongst *Anoxybacillaceae* species and for the demarcation of this family and its several genus-level clades both in phylogenetic and molecular terms. Based upon the presented results we are proposing the division of *Anoxybacillus* species into an emended genus *Anoxybacillus* and three novel genera viz. *Anoxybacteroides* gen. nov. *Paranoxybacillus* gen. nov., and *Thermaerobacillus* gen. nov. Another misclassified *Anoxybacillus* species (viz. *A. sediminis*), which branches with the genus *Brevibacillus*, is transferred into this genus as *Brevibacillus sedimenti* nom. nov.

## Methods

### Phylogenetic tree reconstruction

The genome sequences for 38 *Anoxybacillaceae* species for which annotated protein sequences were available were downloaded from the NCBI database [32]. The available genome sequences for the following species on the NCBI (viz. A. *geothermalis*, accession number: GCA_000948315.1, *Geobacillus lituanicus,* accession number: GCA_002243605.1, *Parageobacillus yumthangensis*, accession number: GCA_002494375.1) are indicated as contaminated. However, as the CheckM analyses of the genomes of * G. lituanicus* and *P. yumthangensis* (results not shown) indicate only 0.6 and 0% contamination*,* respectively, we have included these genomes in our analyses. Additionally, as the species *A. sediminis* in the GTDB is indicated to be related to the genus *Brevibacillus* [18], genomes for several *Brevibacillus* species including its type species, *B. brevis*, were also included. Additionally, the genome sequences for two *Paenibacillaceae* species (viz. *Paenibacillus polymyxa*, *Thermobacillus xylanilyticus*) were included to root the phylogenetic tree due to their phylogenetic proximity to the ingroup and common use as outgroups for this group of organisms [33,34]. Using these genome sequences, a phylogenomic tree was reconstructed using the concatenated sequences of 1148 core proteins from this family. The methods used for the phylogenetic tree reconstruction are described in previous work [35,36]. The CD-HIT program was used to identify those protein families for which homologues sharing a minimum of 50% sequence length and identity were present in at least 80% of the genomes [37]. The sequence alignments of these proteins were trimmed using the TrimAl program [38] prior to their concatenation. The trimmed concatenated alignment used for phylogenetic analysis consisted of 340 955 aligned positions. A maximum-likelihood (ML) tree based on this sequence alignment was initially reconstructed with FastTree2 [39]. Then RaxML [40], employing the Le and Gascuel model [41], was used to optimize this tree and calculate the Shimodaira–Hasegawa (SH)-like statistical support values for each node. Sequence alignment of the core proteins was also used to calculate the AAI between different species pairs [42]. It has been shown in earlier studies that the AAI values based on core proteins are comparable to those based on whole genomes [43]. However, as the AAI values based on core proteins are less impacted by horizontal gene transfers, they show better correlation with the boundaries of different clades [44].

We also reconstructed a phylogenetic tree based on the 16S rRNA gene sequences of the type strains for *Anoxybacillaceae* species and representative *Brevibacillus* species, obtained from the silva rRNA database [45]. The sequences of *Paenibacillus polymyxa* and *Thermobacillus xylanilyticus* were also included to root the tree. The sequences were aligned using the muscle program in the mega X software [46] and trimmed to remove non-conserved regions leaving 1313 aligned positions in the dataset. An ML phylogenetic tree based on it was reconstructed using mega X software [46], based on the Tamura–Nei model [47] and 100 bootstrap replicates.

### Identification of CSIs

Identification of CSIs was carried out as described in earlier work [48,49]. In brief, local blastp searches were conducted on protein sequences from the genomes of representative species from different *Anoxybacillaceae* clades and genera. Based on these blastp searches, sequences of 10–15 high-scoring homologues (E value <1e^−20^) from the clade of interest and 8–10 other outgroup species were obtained and their multiple sequence alignments were examined for the presence of insertions or deletions (indels) of fixed lengths, which were flanked on both sides by at least four conserved amino acid (aa) residues in the neighbouring 40–50 aa and shared by members of specific *Anoxybacillaceae* species clades. A second blastp search was performed on the sequence region that contained the indel and flanking conserved regions (40–50 aa) using the NCBI nr (non-redundant) database. The results for top 500 blastp hits were examined to identify those CSIs, which were present only the species from specific *Anoxybacillaceae* clades and they were formatted using the SIG_CREATE and SIG_STYLE programs [48,49]. Due to limited space, sequence information in the main figures is shown for only a limited number of species from different examined clades. Unless otherwise stated, the described CSIs are exclusively present in all members from an indicated clade. More detailed information for these CSIs is presented in the supplementary data.

## Results

### Phylogenetic analysis of *Anoxybacillaceae* species

**Fig. 1.**
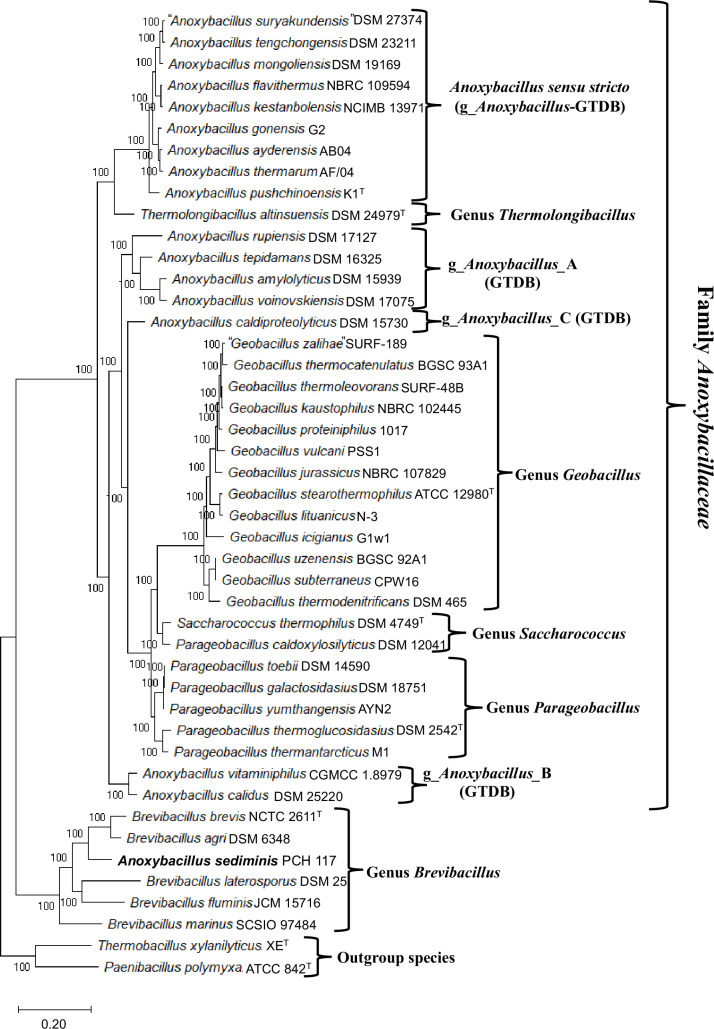
A bootstrapped maximum-likelihood tree for 38 genome-sequenced *Anoxybacillaceae* species based on 1148 core proteins. The statistical support values for different branches are indicated on the nodes. The tree was rooted as indicated in the Methods section. Non-validly published species are shown within quotation (“ ”) marks and type species are identified with a superscript ^T^. Different main species clades observed in the tree are labelled by the genus names or other designated names. The GTDB taxon names for some clades are also indicated.

**Fig. 2.**
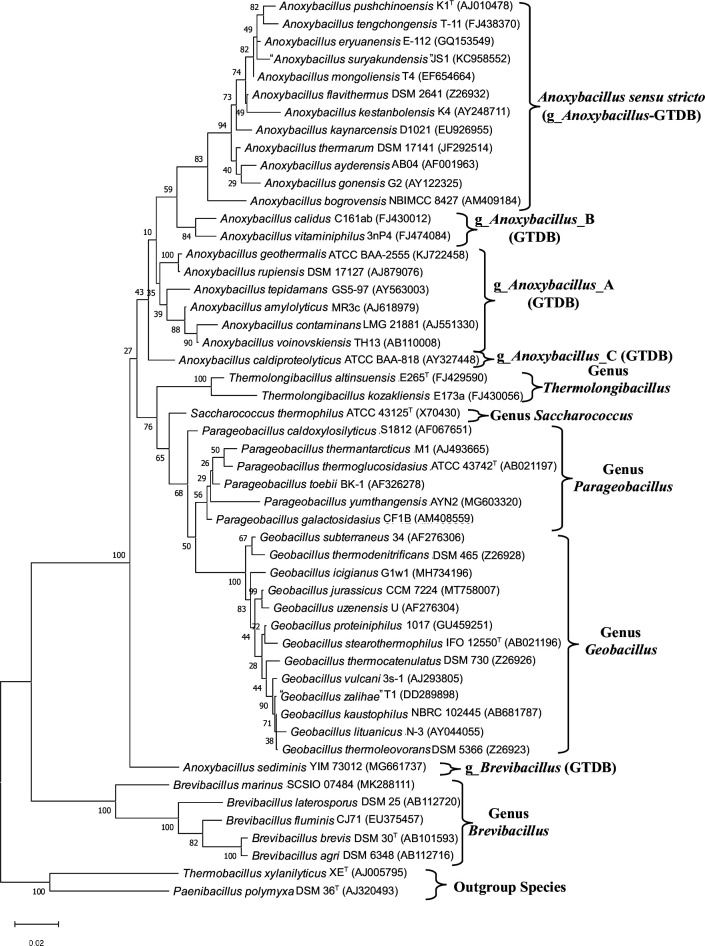
A maximum-likelihood phylogenetic tree based on 16S rRNA gene sequences for the type strains of all *Anoxybacillaceae* species. The tree was rooted as indicated in the Methods section. The accession numbers of the 16S rRNA gene sequences are provided within brackets after each species name in the tree. Different main clades within the tree are marked with the names of the genera or other designated names as in Fig. 1.

To delineate the evolutionary relationships among *Anoxybacillaceae* species, we reconstructed an ML phylogenetic tree for 38 available genome-sequenced species based on concatenated sequences of 1148 core proteins. This tree, presented in Fig. 1, is the most comprehensive tree reconstructed for this group of species and it will be referred to as the core protein tree. In this tree, all the observed nodes are supported by 100% SH-like statistical support values, indicating that the observed evolutionary relationships amongst *Anoxybacillaceae* species are trustworthy. We also reconstructed a phylogenetic tree based on 16S rRNA gene sequences that included information for all validly named *Anoxybacillaceae* species including *A. bogrovensis*, *A. contaminans*, *A. eryuanensis*, *A. geothermalis, A. kaynarcensis* and *Thermolongibacillus kozakliensis*, for which genome sequences were not available (Fig. 2). Of the examined *Anoxybacillus* species, a recent study indicates that *A. geothermalis*, which branches with * A. rupiensis* in the 16S rRNA tree, is a later heterotypic synonym of *A. rupiensis* [50]. The overall branching pattern and the clusters formed by the species in the two trees are generally very similar. As the core protein tree showed higher statistical support for different nodes, this tree was used to draw most of the phylogenetic inferences. However, any significant differences seen between the two trees are noted below.

Based on the reconstructed trees, several insights into the evolutionary relationships among *Anoxybacillaceae* species can be drawn. One prominent observation from both trees is that the *Anoxybacillus* species formed multiple distinct clades (Figs1  2), which in the core protein tree (Fig. 1) were interspersed between other *Anoxybacillaceae* genera. The type species of genus *Anoxybacillus*, *A. pushchinoensis*, grouped within a clade labelled as the *Anoxybacillus sensu stricto* in Figs1  2. The species composition of this clade and the other three *Anoxybacillus* clades in our core protein tree is similar to that in the GTDB taxonomy [18]. Following the designations used in the GTDB, we have labelled the other three *Anoxybacillus* species clades as *Anoxybacillus_*A, *Anoxybacillus_*B, and *Anoxybacillus_*C in Figs1  2. The species *Anoxybacillus sediminis* branched outside of the clade consisting of all other *Anoxybacillaceae* clades/genera in both our core protein tree and the tree based on 16S rRNA gene sequences. In the GTDB taxonomy, this species is assigned to the genus *Brevibacillus* [18]. In accordance with this assignment, in our core protein tree (Fig. 1), this species grouped reliably with the species from genus *Brevibacillus*. Of the other *Anoxybacillaceae* genera for which multiple genome sequences were available, *Geobacillus* species formed a strongly supported clade in the trees (Figs1  2). Species from the genera *Parageobacillus* and *Saccharococcus* formed outgroups of the *Geobacillus* clade. In our phylogenetic trees, species from these two genera did not form monophyletic clades. In the core protein tree (Fig. 1), while the other *Parageobacillus* species grouped together, *P. caldoxylosilyticus* was found to branch with the species *S. thermophilus*. The distinct branching of *P. caldoxylosilyticus* from other *Parageobacillus* species is also observed in earlier work [25]. It should be noted that *P. caldoxylosilyticus* was originally described as *Saccharococcus caldoxylosilyticus* [51] and later transferred to the genus *Parageobacillus* [20]. However, based on its branching, the GTDB taxonomy also places this species into the genus *Saccharococcus* [18].

Using the sequence alignment of the core proteins, we have also determined the pairwise average amino acid identity (AAI) between different *Anoxybacillaceae* species. A matrix of these results is shown in Table S1, available in the online version of this article, where a darker shade of green indicates higher sequence similarities between the species pairs. As shown Table S1, the different clades formed by *Anoxybacillaceae* species generally showed higher AAI values in comparison to the species from other clades Notably, the intra-clade AAI values for the *Anoxybacillus sensu stricto*, *Anoxybacillus*_A and *Anoxybacillus*_B clades, which consisted of multiple species, ranged from 79% to greater than 95%. In comparison, inter-clade AAI values between different *Anoxybacillus* species clades were much lower, ranging between 58 and 81%. Species from the genus *Geobacillus* were also clearly distinguished based on their AAI values. However, in contrast to these species’ clades, based on AAI values no distinction between species from the genera *Parageobacillus* and *Saccharococcus* could be made.

### Identification of molecular markers specific for different *Anoxybacillus* species clades and other clades/genera within the family *Anoxybacillaceae*

The results from our phylogenetic trees (Figs1  2), and other studies in the literature (see Introduction) including the GTDB [18], show that *Anoxybacillus* species consistently form four distinct clades, apart from the species *A. sediminis*, which branches with the genus *Brevibacillus*. To obtain further independent evidence supporting the distinctness of these *Anoxybacillus* species clades, and to demarcate them reliably, we have conducted detailed comparative analyses on protein sequences from *Anoxybacillaceae* genomes to identify conserved signature indels (CSIs), which are specific for the members of different clades. Earlier studies have shown that the CSIs in gene/protein sequences which are uniquely found in a monophyletic group of species, represent molecular synapomorphies providing reliable means for discerning evolutionary relationships in molecular terms [36,49, 52,55]. Our analyses of protein sequences from *Anoxybacillaceae* genomes have identified 39 novel CSIs specific for different *Anoxybacillus* species clades and other clades/genera within this family, offering strong independent evidence supporting the distinctness of these clades in molecular terms. The group-specificities and characteristics of the identified CSIs are described below.

### Molecular markers delineating different *Anoxybacillus* species clades

**Fig. 3.**
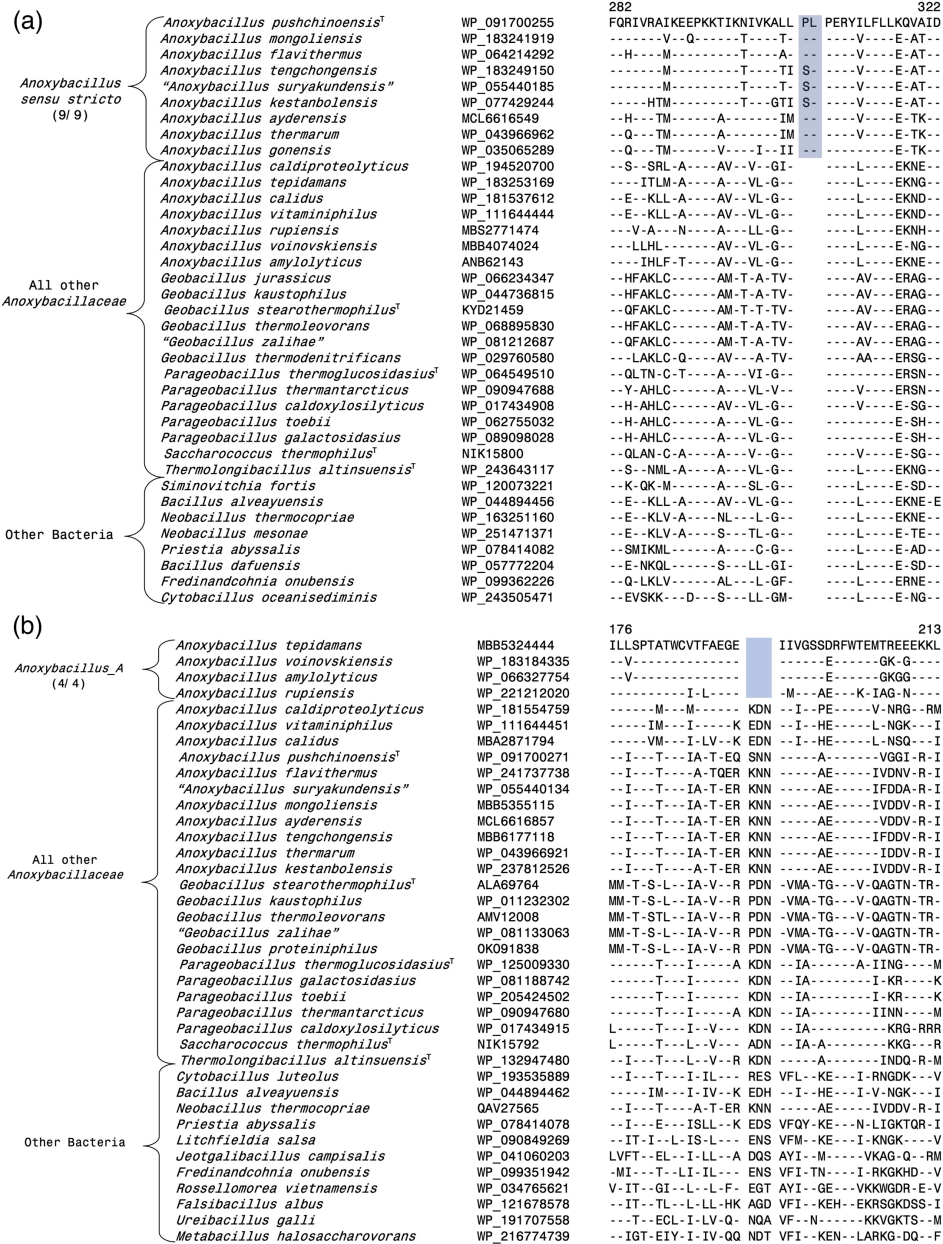
Examples of molecular markers specific for *Anoxybacillus sensu stricto* and the *Anoxybacillus*_A clades. The dashes (-) in this and all other sequence alignments indicate identity with the amino acids on the top line. Gaps in sequence alignment indicate that no amino acid is present in that position. Accession numbers for different sequences are indicated in the second column and the position of this sequence fragment within the protein is indicated above the sequences. (**a**) Partial sequence alignment of the protein NAD(P)/FAD-dependent oxidoreductase showing a two aa insert (highlighted) that is exclusively shared by all species from the *Anoxybacillus sensu stricto* clade. (**b**) Partial sequence alignment of the nuclease-related domain-containing protein showing a three aa deletion (highlighted) that is specific for the species from *Anoxybacillus*_A clade.

Of the four main clades formed by the *Anoxybacillus* species, the *Anoxybacillus sensu stricto* clade containing the type species * A. pushchinoensis*, encompasses the largest number of species. Our analyses have identified eight CSIs that are uniquely shared by the species from this group and not present in other *Anoxybacillaceae* species, or other bacteria. One example of a CSI specific for this clade is shown in Fig. 3a, where a 2 aa insert in a conserved region of the protein NAD(P)/FAD-dependent oxidoreductase is exclusively shared by all species from this clade but not found in the protein homologues of other *Anoxybacillus* species. Additional information for this CSI and the seven other CSIs specific for this clade is presented in Figs S1–S8 [56], and some of their characteristics are summarized in Table 1. In our core protein tree (Fig. 1), the species *T. altinsuensis* forms an outgroup of the *Anoxybacillus sensu stricto* clade. Our work has identified four CSIs which, except for an isolated exception, are uniquely shared by the *Anoxybacillus sensu stricto* clade species and *T. altinsuensis*. Sequence information for these CSIs is provided in Figs S9–S12 [56], and Table 1 and they provide evidence that the species from these two clades/genera are closely related.

**Table 1.** Summary of CSIs specific for different *Anoxybacillus* species clades

Protein name	Accession no.	Indel size	Indel position	Figure no.	Specificity
NAD(P)/FAD-dependent oxidoreductase	WP_091700255	2 aa Ins	282–322	Fig. 3AFig. S1	*Anoxybacillus sensu stricto*
Cof-type HAD-IIB family hydrolase	WP_091701995	2 aa Del	101–147	Fig. S2	
Intercompartmental signalling factor BofC	WP_244149206	1 aa Del	59–96	Fig. S3	
Alanine racemase*	WP_091704058	2 aa Ins	59–100	Fig. S4	
Glutamate synthase subunit beta*	WP_091700721	1 aa Del	263–306	Fig. S5	
Glyceraldehyde-3-phosphate dehydrogenase*†	WP_091702457	1 aa Del	117–156	Fig. S6	
FAD-dependent oxidoreductase*	WP_091704556	1 aa Del	58–89	Fig. S7	
GNAT family *N*-acetyltransferase*	WP_091705020	1 aa Del	78–109	Fig. S8	
O-succinylbenzoate-CoA ligase*	WP_208601848	1 aa Del	272–312	Fig. S9	*Anoxybacillus sensu stricto* and *Thermolongibacillus*
Carboxypeptidase M32*	WP_091702529	1 aa Del	301–351	Fig. S10	
3-methyl-2-oxobutanoate hydroxymethyltransferase*	WP_091702745	1 aa Del	66–111	Fig. S11	
Iron-containing alcohol dehydrogenase*	WP_091700886	1 aa Del	8–49	Fig. S12	
Nuclease-related domain-containing protein	MBB5324444	3 aa Del	176–213	Fig. 3BFig. S13	*Anoxybacillus_*A
DUF2268 domain-containing protein	WP_027408930	2 aa Del	112–160	Fig. S14	
YpmS family protein*	WP_183255419	1 aa Del	72–124	Fig. S15	
sporulation inhibitor KapD*	MBB5324401	1 aa Del	67–109	Fig. S16	
Excinuclease ABC subunit UvrC	WP_111644581	1 aa Ins	99–136	Fig. 4aFig. S17	*Anoxybacillus_*B
Cytochrome c oxidase subunit II	WP_111643591	1 aa Ins	178–223	Fig. S18	
Rod shape-determining protein MreC*	WP_111644636	1 aa Ins	140–174	Fig. S19	
Heavy metal translocating P-type ATPase	WP_181556046	4 aa Del	165–207	Fig. S20	*Anoxybacillus_*C
DUF2225 domain-containing protein	WP_181556420	1 aa Ins	63–116	Fig. S21	

*Isolated exception present in distantly related species.

§†hHomologue not detected in some species.

Of the other observed *Anoxybacillus* species clades (Figs1  2), the *Anoxybacillus_*A clade harbours the species *A. amylolyticus*, *A. rupiensis. A. tepidamans* and *A. voinovskiensis*. Based on the species branching in the 16S rRNA tree (Fig. 2) [10,12], two other species viz*. A. contaminans* and *A. geothermalis* are also part of this clade. Our analyses have identified four CSIs that are exclusively shared by all four genome-sequenced species of this clade, providing dependable means for the demarcation of this clade. Sequence information for one of the CSIs specific for this clade is presented in Fig. 3b, where a 3 aa deletion in the nuclease-related domain-containing protein is present in all four species from *Anoxybacillus_*A clade but absent in all other *Anoxybacillaceae* species. Detailed sequence information for this CSI and three other CSIs specific for the *Anoxybacillus_*A is presented in Figs S13–S16 [56], and some of their characteristics are summarized in Table 1.

**Fig. 4.**
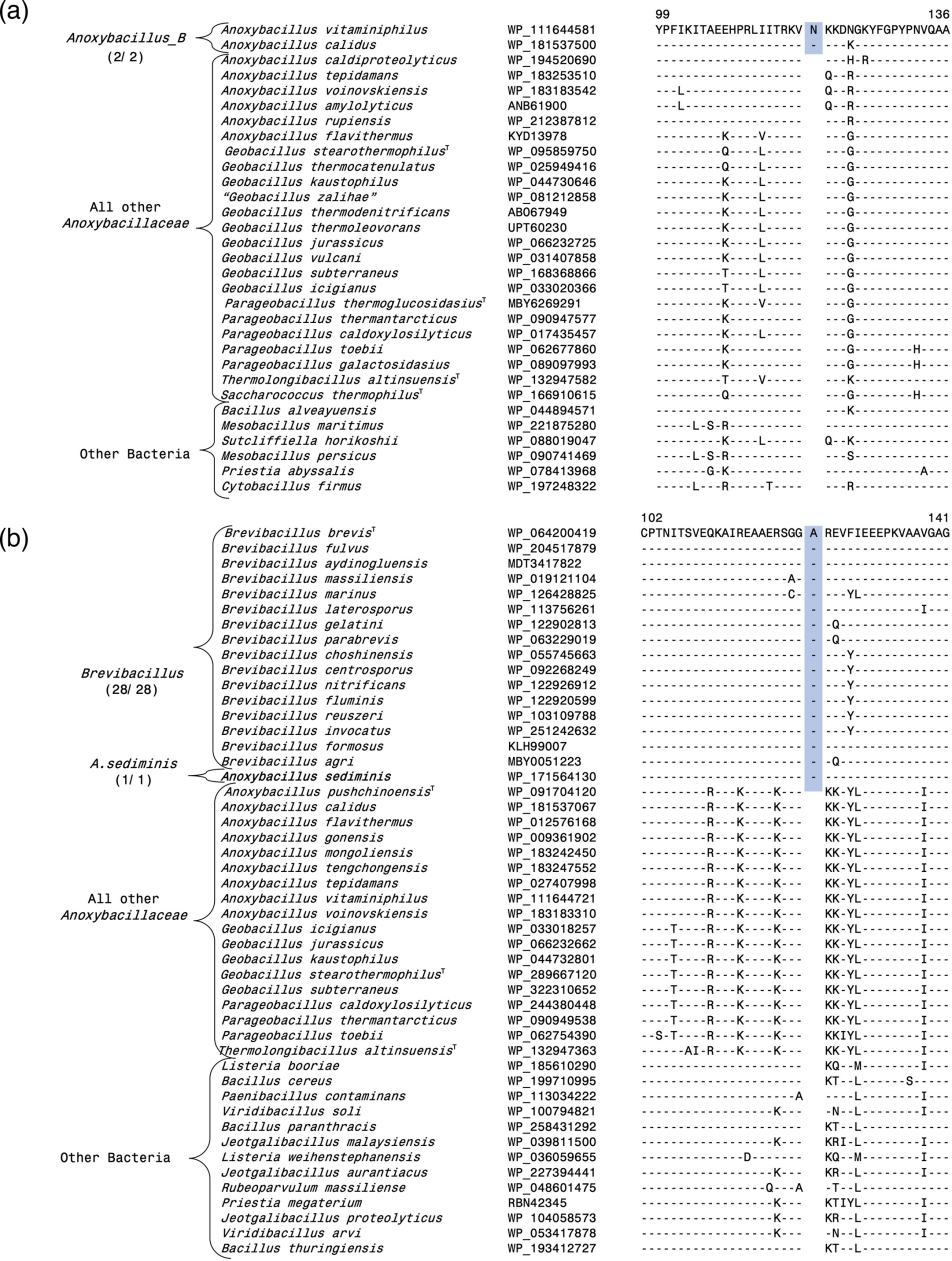
Examples of molecular markers specific for the *Anoxybacillus*_B clade and the genus *Brevibacillus*. (**a**) Partial sequence alignment of the nuclease-related domain-containing protein showing a one aa insert (highlighted) that is exclusively shared by all species from the *Anoxybacillus*_B clade (**b**) Partial sequence alignment of the rod shape-determining protein showing a one aa insert (highlighted) that is generally exclusively shared by species from the genus *Brevibacillus* and *A. sediminis*.

Our work has also identified three CSIs, which are specific for the *Anoxybacillus_*B clade consisting of the species *A. calidus* and *A. vitaminiphilus*. These two species also formed a distinct clade in earlier phylogenetic studies [10,11, 13, 31]. An example of a CSI specific for the *Anoxybacillus_*B clade is shown in Fig. 4a, where a 1 aa insert in the protein excinuclease ABC subunit UvrC, is only present in the two species from this clade but not found in any other *Anoxybacillus* species or other bacteria. More detailed information for this CSI and the sequence information for the other two CSIs specific for the *Anoxybacillus_*B clade is provided in Figs S17–S19 [56], and some of their characteristics are listed in Table 1. The *Anoxybacillus*_C clade consists only of the species *A. caldiproteolyticus*, which branches distinctly from all others. We have identified two CSIs (Figs S20–S21 and Table 1) [56], which are uniquely found in this species supporting its distinctness from others.

The species *A. sediminis* was placed into the genus *Anoxybacillus* based on its branching in the 16S rRNA tree [13]. The described tree contained only limited number of closely related species, and in it *A. sediminis* formed the deepest branching lineage within the species from *Anoxybacillaceae* family [13]. However, based upon the GTDB taxonomy [18], and as seen in on our core protein tree (Fig. 1), the species *A. sediminis* branches reliably with members of the genus *Brevibacillus*. This inference is also supported by seven CSIs identified by our analyses that are generally uniquely shared by *A. sediminis* and different *Brevibacillus* species. Sequence information for one of these CSIs is shown in Fig. 4b, where a 1 aa insert in the rod-shape determining protein is commonly shared by different *Brevibacillus* species and *A. sediminis*, but not found in any other *Anoxybacillaceae* species, and other bacteria. More detailed sequence information for this CSI and six other CSIs showing similar specificities is presented in Figs S22–S28 [56], and some of their properties are shown in Table 2.

**Table 2.** Summary of CSIs specific for the family *Anoxybacillaceae*, genera *Brevibacillus* and *Geobacillus*, and a clade consisting of *Geobacillus–Parageobacillus–Saccharococcus* genera

Protein name	Accession no.	Indel size	Indel position	Figure no.	Specificity
Rod shape-determining protein*	WP_064200419	1 aa Ins	102–141	Fig. 4bFig. S22	Genus *Brevibacillus+A. sediminis*
Agmatinase*†	WP_016740148	1 aa Del	162–205	Fig. S23	
Fur family peroxide stress response transcriptional regulator *†	RED27321	1 aa Ins	103–143	Fig. S24	
Outer membrane lipoprotein-sorting protein*†	WP_188066926	1 aa Ins	146–180	Fig. S25	
Proline dehydrogenase*†	WP_048031337	1 aa Ins	104–139	Fig. S26	
Uroporphyrinogen-III C-methyltransferase†*	WP_106655671	1 aa Ins	206–240	Fig. S27	
Lrp/AsnC family transcriptional regulator*†	WP_012684838	1 aa Ins	84–125	Fig. S28	
Electron transfer flavoprotein beta subunit	OAO76761	1 aa Ins	27–65	Fig. 5aFig. S29	Genus *Geobacillus*
Dihydroxy-acid dehydratase*	KAF6510349	1 aa Ins	295–328	Fig. S30	
Aminotransferase class I/II-fold pyridoxal phosphate-dependent enzyme	WP_095858690	1 aa Ins	84–117	Fig. S31	
GNAT family protein	WP_033016698	2 aa Del	124–164	Fig. 5bFig. S32	*Geobacillus- Parageobacillus-Saccharococcus* cluster
NYN domain-containing protein*†	WP_064213561	1 aa Ins	6–55	Fig. S33	
Ribonuclease J	WP_235597858	1 aa Del	95–145	Fig. S34	
DNA-binding protein WhiA *†	WP_091702361	1 aa Ins	143–192	Fig. 6Fig. S35	Family *Anoxybacillaceae*
SNF2-related protein*†	WP_091700607	1 aa Del	212–267	Fig. S36	
Heat-inducible transcriptional repressor HrcA*†	WP_091703604	1 aa Ins	238–293	Fig. S37	
HslU-HslV peptidase ATPase subunit*†	WP_091699997	1 aa Del	101–150	Fig. S38	
Bifunctional (p)ppGpp synthetase/guanosine-3′,5′-bis(diphosphate) 3′-pyrophosphohydrolase *†	WP_091702253	1 aa Ins	669–719	Fig. S39	

*Isolated exception present in distantly related species.

§†hHomologue not detected in few species.

**Fig. 5.**
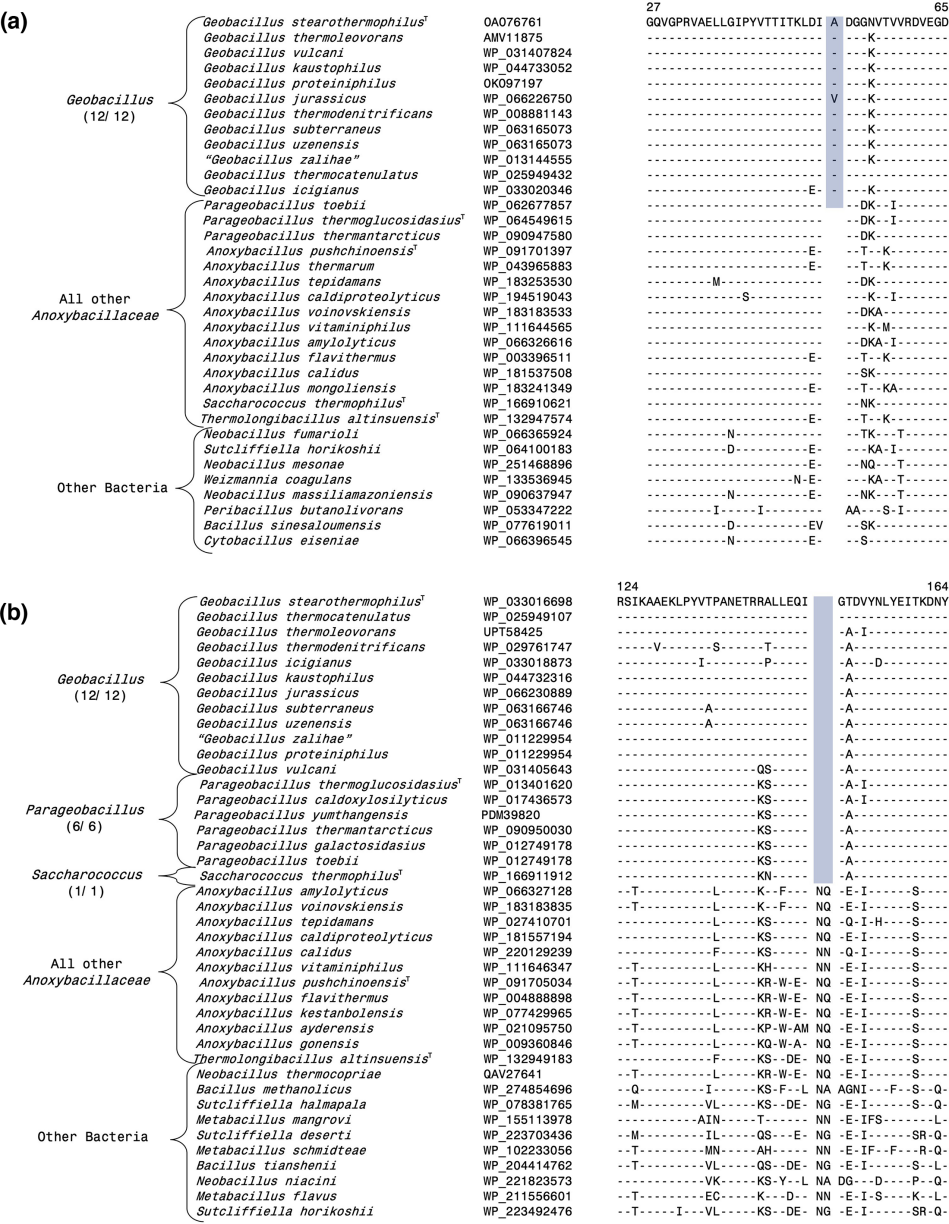
Examples of molecular markers specific for the genus *Geobacillus* and a clade consisting of the genera *Geobacillus*, *Parageobacillus* and *Saccharococcus*. (**a**) Partial sequence alignment of the protein electron transfer flavoprotein beta subunit showing a one aa insert (highlighted) that is exclusively shared by all species from the *Geobacillus.* (**b**) Partial sequence alignment of the GNAT family protein showing a two aa deletion (highlighted) that is exclusively shared by species from the genera *Geobacillus*, *Parageobacillus* and *Saccharococcus*.

### CSIS specific for the genus *Geobacillus* and family *Anoxybacillaceae*

*Geobacillus* species form a strongly supported clade in the phylogenetic trees shown in Figs1  2. We have also identified three CSIs in different proteins that are generally exclusively shared by all *Geobacillus* species. One example of a *Geobacillus* specific CSI is shown in Fig. 5a, where a 1 aa insert in the protein electron transfer flavoprotein beta subunit is uniquely found in different *Geobacillus* species but absent in other *Anoxybacillaceae* species, as well as other bacteria. Detailed sequence information for all three CSI specific for the genus *Geobacillus* is provided in Figs S29–S31 [56], and some of their properties are listed in Table 2. In our phylogenetic trees (Figs1  2), species from the genera *Geobacillus*, *Parageobacillus* and *Saccharococcus* form a strongly supported clade. It should be noted that the genus *Parageobacillus* was created by the transfer of several deeper branching *Geobacillus* species into this new genus [20]. A close relationship of the species from these three genera is also supported by three CSIs identified by our work that are generally exclusively shared by the species from these genera. An example of one of these CSIs is shown in Fig. 5b, where a 2 aa insert in the GNAT family protein is commonly shared by species from *Geobacillus, Parageobacillus* and *Saccharococcus*. Sequence information for these CSIs is presented in Figs S32–S34 [56], and some of their characteristics are listed in Table 2. In contrast to the CSIs specific for the clades described above, our analyses did not identify any CSI that was uniquely shared by *P. caldoxylosilyticus* and *S. thermophilus*, or by the other four *Parageobacillus* species.

**Fig. 6.**
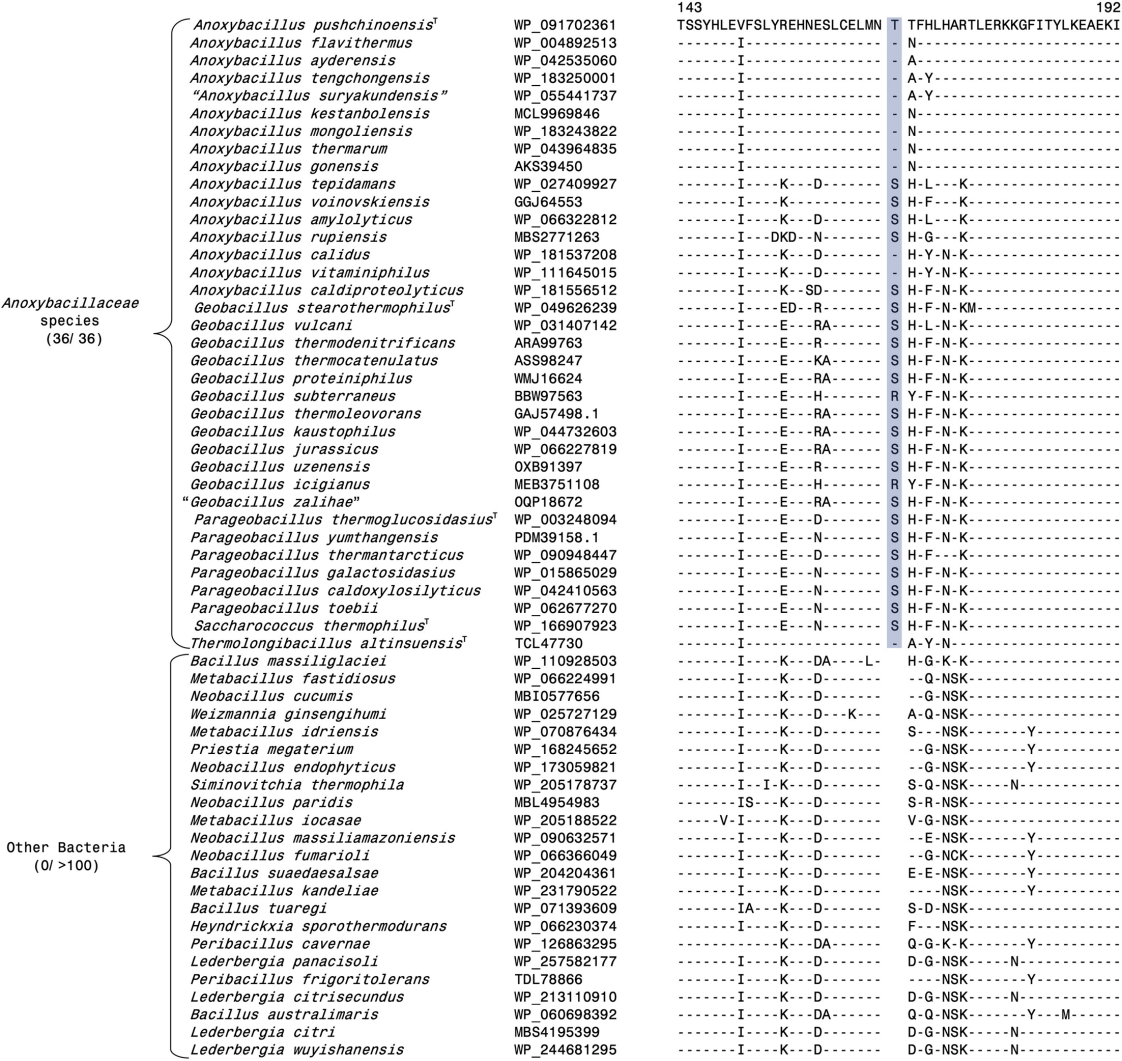
Partial sequence alignment of the protein DNA-binding protein WhiA showing a one aa insert (highlighted) that is specific for the species from the family *Anoxybacillaceae*.

The family *Anoxybacillaceae* is presently demarcated based on the relative evolutionary divergence of a clade consisting of these species in the GTDB tree (Fig. 1) [17]. However, our work has now identified five CSIs in diverse proteins that are generally exclusively shared by different *Anoxybacillaceae* species providing molecular means for the demarcation of this family. One example of a CSI specific for this family is shown in Fig. 6, where a 1 aa insert in the DNA-binding protein WhiA is uniquely shared by different *Anoxybacillaceae* species. Detailed sequence information for this CSI and the four others specific for this family is presented in Figs S35–S39 [56], and some of their properties are listed in Table 2.

## Discussion

The present study was undertaken to clarify the evolutionary relationships among members of the family *Anoxybacillaceae* [17], which contains several species that are useful for industrial processes such as the bioremediation of wastewater and starch hydrolysis [7,14, 16]. Within the family *Anoxybacillaceae*, species from the genus *Anoxybacillus*, which holds >50% of the members are known to be polyphyletic and they form multiple unrelated clades in earlier phylogenetic studies [4,10,12, 28]. In the GTDB taxonomy [18], species from this genus are assigned to four genus-level clades apart from the species *A. sediminis*, which is indicated to group with *Brevibacillus*. Therefore, the main objective of this study was to reliably understand the evolutionary relationships amongst the *Anoxybacillus* species and dependably demarcate different genus-level clades formed by these species.

**Fig. 7.**
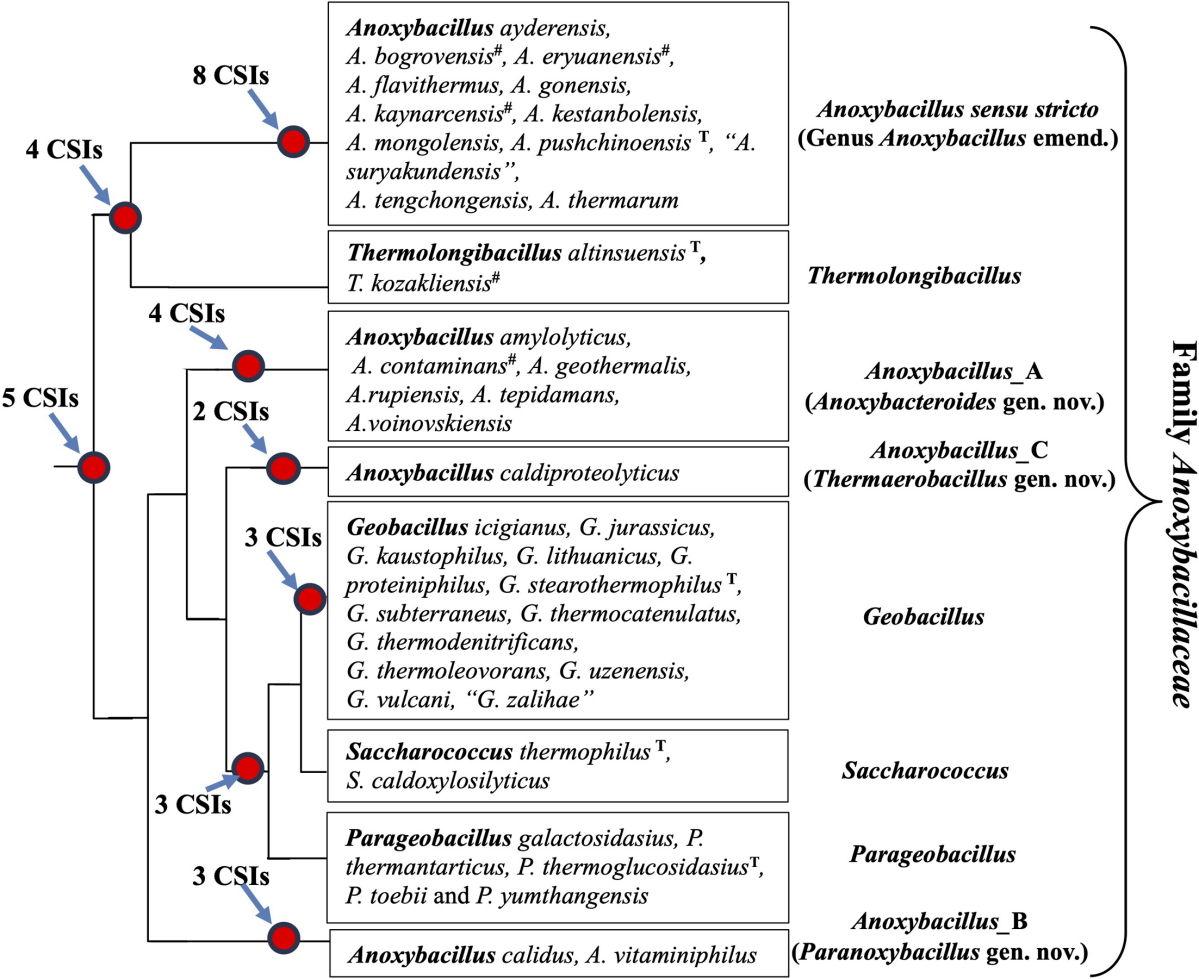
A conceptual diagram summarizing results from our phylogenomic and comparative genomic analyses on *Anoxybacillaceae* species. The placement of the species into different genus-level clades is based on different lines of evidence presented in this work. This diagram also shows the numbers of identified CSIs specific for different species clades and also the proposed taxonomic revisions. The superscript ^#^ indicates that the placement of these non-genome sequenced species into the indicated genus is based on branching in the 16S rRNA gene tree. Apart from this, our work also provides evidence that *A. sediminis* is specifically related to the genus *Brevibacillus* and we are proposing its reclassification as *Brevibacillus sedimenti* nom. nov.

In this study, using genome sequences for 38 *Anoxybacillaceae* species, we have comprehensively examined the evolutionary relationships among these species using multiple independent approaches. These approaches included, the reconstruction of phylogenetic trees based on 1148 core genome proteins and 16S rRNA gene sequences, AAI analyses, as well as analyses of protein sequences from their genomes to identify molecular markers (CSIs), which are specific for different observed clades enabling their robust demarcation in molecular terms. The results from these studies are summarized in the form of a conceptual diagram (based on the core protein tree) shown in Fig. 7.

The results presented here provide consistent and robust evidence demonstrating that the *Anoxybacillus* species are not monophyletic but instead form four distinct clades, which can be reliably distinguished from each other based on their branching in phylogenetic trees and multiple uniquely shared molecular signatures. The species compositions of these four clades is in agreement with the GTDB taxonomy [18]. Of these four clades, the type species of genus *Anoxybacillus*, *A. pushchinoensis*, is a part of the *Anoxybacillus sensu stricto* clade. Hence, in accordance with the International Code of Nomenclature for Prokaryotes [57], this clade represents the genus *Anoxybacillus*. Therefore, we are proposing an emended description of the genus *Anoxybacillus* to limit it only to the species from the *Anoxybacillus sensu stricto* clade. Our results also provide compelling evidence that the *Anoxybacillus_*A, *Anoxybacillus_*B and *Anoxybacillus*_C clades are distinct from the genus *Anoxybacillus* clade*,* and we have identified multiple molecular markers specific for these clades providing trustworthy means for their demarcation. Based upon the presented evidence, we are proposing the transfer of species from *Anoxybacillus_*A, *Anoxybacillus_*B and *Anoxybacillus*_C clades to three novel genera *Anoxybacteroides* gen. nov., *Paranoxybacillus* gen. nov., and *Thermaerobacillus* gen. nov., respectively. Lastly, our studies also provide convincing evidence that the species *A. sediminis* was misclassified into the genus *Anoxybacillus* [13], and it is specifically related to the genus *Brevibacillus*. Hence, we are proposing the transfer of this species to the genus *Brevibacillus* as *Brevibacillus sedimenti* nom. nov.

Besides clarifying the evolutionary relationships among *Anoxybacillus* species, this work has also identified five novel CSIs that are specific for the family *Anoxybacillaceae* and provide robust means for its demarcation in molecular terms. Additionally, three other CSIs described here reliably distinguish members of the genus *Geobacillus* from all other genera. The close relationship of *Geobacillus* to the species from genera *Parageobacillus* and *Saccharococcus* is also strongly supported by our phylogenetic trees and three CSIs, which are uniquely shared by the species from these genera. In our core protein tree (Fig. 1), species from the genera *Parageobacillus* and *Saccharococcus* are separated by very short branches and the distinction between them is not clear based on AAI values (Table S1). Of these species, in the core protein tree (Fig. 1), *P. caldoxylosilyticus* branches with *S. thermophilus* and the GTDB also assigns this species into the genus *Saccharococcus* [18]. The species *P. caldoxylosilyticus* was also originally described as *S. caldoxylosilyticus* [51] but later transferred to *Parageobacillus* [20]. Although our work has not identified any CSI that is uniquely shared by *P. caldoxylosilyticus* and *S. thermophilus*, based on the branching of *P. caldoxylosilyticus* with *S. thermophilus* in our core protein tree and the GTDB tree, we are proposing that this species should be known by its original name, i.e. *Saccharococcus caldoxylosilyticus*, and *Parageobacillus caldoxylosilyticus* should be considered a later heterotypic synonym of this name.

Earlier work on CSIs specific for several other groups of bacteria including many *Bacilli* genera [36,49, 52, 58,60] provide compelling evidence that these molecular characteristics exhibit a high degree of predictive ability to be found in other members of a given genus/clade [53,54, 61, 62]. Based upon the predictive abilities of the CSIs to be found in other species/strain from a specific taxon, we have recently developed a webserver (https://appindels.com/), which based upon the presence of taxon-specific CSIs in a submitted genome, can predict with high accuracy (~100%) its taxonomic affiliation [62]. Thus, the CSIs identified in this work should prove useful in the classification of other uncharacterized or novel *Anoxybacillaceae* species. Additionally, earlier work shows that the genetic changes responsible for them are functionally important for the group of organisms containing them [63,65]. Hence, further studies on understanding the functional significance of these CSIs could lead to the identification of novel biochemical and/or other characteristics of the CSIs-harbouring micro-organisms. Lastly, due to the presence of these CSIs in conserved regions of genes/proteins, their sequences could also be used for the development of novel diagnostic methods for the indicated groups of organisms by *in silico* analyses and commonly used experimental methods [66,69].

The descriptions of the proposed novel and amended genera/family are given below.

## Emended description of the family *Anoxybacillaceae* Chuvochina *et al.* 2024

The family *Anoxybacillaceae* was described by Chuvochina *et al.* [17] based on the branching and relative evolutionary divergence of this species clade in the GTDB tree [18]. The family contains the type genus *Anoxybacillus* and the following genera: *Anoxybacteroides*, *Paranoxybacillus*, *Geobacillus*, *Parageobacillus*, *Saccharococcus*, *Thermaerobacillus*, and *Thermolongibacillus.* Members of this family form a monophyletic clade in phylogenetic trees based on 16S rRNA gene sequences and trees based on large datasets of concatenated proteins. Five CSIs described in this work (Table 2), found in the proteins DNA-binding protein WhiA, SNF2-related protein, Heat-inducible transcriptional repressor HrcA, HslU-HslV peptidase ATPase subunit and Bifunctional (p)ppGpp synthetase/guanosine-3′,5′-bis(diphosphate) 3′-pyrophosphohydrolase, are also generally uniquely found in the species from this family.

The type genus is *Anoxybacillus* Pikuta *et al*. 2000.

## Emended description of the genus Anoxybacillus Pikuta *et al*. 2000

The description of this genus is partially based on that given by Pikuta *et al.* [2]. Species are Gram-stain-positive, rod-shaped, and spore-forming. Spores are located terminally or centrally. Most species are facultatively anaerobic and motile. Some species are anaerobic and non-motile. Species are catalase and oxidase variable. Species have been isolated from manure, mud and water samples from hot springs. The optimal NaCl concentration is between 1.0 and 2.5% and the species tolerate up to 5%. Species are moderately thermophilic with a temperature range for growth of 30–75 °C (optimal, 50–65 °C). Species can grow between pH of 5.0 and 11.5 (optimal between pH 7.2 and 9.7). The genome size ranges from 2.6 to 2.9 Mb and the G+C content varies between 41.1 to 54.0 mol%. Species from this genus form a distinct clade in phylogenetic trees based on concatenated sequences of large numbers of proteins and in trees based on 16S rRNA gene sequences. Additionally, members of this genus can be reliably distinguished from all other *Anoxybacillaceae* genera based on eight CSIs described in this work (Table 1), which in most cases are exclusively found in these species. These CSIs are present in the following proteins: NAD(P)/FAD-dependent oxidoreductase, Cof-type HAD-IIB family hydrolase, intercompartmental signalling factor BofC, alanine racemase, glutamate synthase subunit beta, glyceraldehyde-3-phosphate dehydrogenase, FAD-dependent oxidoreductase and GNAT family *N*-acetyltransferase.

The type species is *Anoxybacillus pushchinoensis* corrig. Pikuta *et al*. 2000.

## Description of *Anoxybacteroides* gen. nov.

*Anoxybacteroides* (A.no.xy.bac.te.ro’i.des. Gr. pref. *an-*, without (here: inseparable prefix); Gr. masc. adj. *oxys*, acid or sour and in combined words indicating oxygen; N.L. masc. n. *bacter*, a rod; L. adj. suff. -*oides*, resembling, similar; from Gr. neut. adj. suff. -*eides*, resembling, similar; from Gr. neut. n. *eîdos*, that which is seen, form, shape, figure; N.L. masc. n. *Anoxybacteroides*, a rod-like organism that lives without oxygen).

Species are facultatively anaerobic, facultatively aerobic, or obligately aerobic rod-shaped and spore-forming bacteria. Spores are located terminally. The members of this genus are mainly motile. Most species are Gram-stain-positive, except for *A. contaminans*, which is described as Gram-stain-variable. Species are catalase-positive and oxidase-variable. Species were isolated from plant byproducts, hot springs, geothermal soils and reservoirs. Members are moderately thermophilic and grow at a temperature ranging from 30 to 67 °C (optimal at 54–61 °C). Growth occurs at pH 5.0–9.0 (optimal at 5.0–6.5) and NaCl concentration up to 5.0%. The genome size ranges from 3.1 to 3.7 Mb and their G+C content varies between 41.7 to 44.4 mol%. Species from this genus form a distinct clade in phylogenetic trees based on concatenated sequences of large numbers of proteins and in trees based on 16S rRNA gene sequences. Additionally, members of this genus can be reliably distinguished from all other *Anoxybacillaceae* genera based on four CSIs described in this work (Table 1), which in most cases are exclusively found in these species. These CSIs are found in the following proteins: nuclease-related domain-containing protein, DUF2268 domain-containing protein, YpmS family protein, and sporulation inhibitor KapD.

The type species is *Anoxybacteroides amylolyticus*.

## Description of *Anoxybacteroides amylolyticus* comb. nov.

*Anoxybacteroides amylolyticus* (a.my.lo.ly’ti.cus. Gr. neut. n. *amylon*, starch; Gr. masc. adj. *lytikos*, able to dissolve; N.L. masc. adj. *amylolyticus*, starch-dissolving).

Basonym: *Anoxybacillus amylolyticus* Poli *et al.* 2006.

The description of this species is as provided by Poli *et al.* [7] for *Anoxybacillus amylolyticus.*

The type strain is ATCC BAA-872^T^=CIP 108338^T^=DSM 15939^T^=MR3C^T^.

## Description of *Anoxybacteroides contaminans* comb. nov.

*Anoxybacteroides contaminans* (con.ta’mi.nans. L. part. adj. *contaminans*, contaminating).

Basonym: *Anoxybacillus contaminans* De Clerck *et al.* 2004.

The description of this species is as provided by De Clerck *et al.* [70] for *Anoxybacillus contaminans*.

The type strain is DSM 15866^T^=LMG 21881^T^.

## Description of *Anoxybacteroides geothermalis* comb. nov.

*Anoxybacteroides geothermalis* (ge.o.ther.ma’lis. Gr. n. *ge-*, earth; N.L. fem. adj. *thermalis*, of thermal properties or origin; N.L. masc. adj. *geothermalis*, from hot earth, from a geothermal site).

Basonym: *Anoxybacillus geothermalis* Filippidou *et al*. 2016.

The description of this species is as provided by Filippidou *et al.* [11] for *Anoxybacillus geothermalis*.

The type strain is ATCC BAA-2555^T^=CCOS 808^T^=GSsed3^T^.

## Description of *Anoxybacteroides rupiensis* comb. nov.

*Anoxybacteroides rupiensis* (ru.pi.en’sis. N.L. masc. adj. *rupiensis*, originating from Rupi Basin, referring to the place of isolation of the type strain).

Basonym: *Anoxybacillus rupiensis* Derekova *et al.* 2008.

The description of this species is as provided by Derekova *et al.* [26] for *Anoxybacillus rupiensis*.

The type strain is DSM 17127^T^=NBIMCC 8387^T^=R270^T^.

## Description of *Anoxybacteroides tepidamans* comb. nov.

*Anoxybacteroides tepidamans* (te.pid.a’mans. L. masc. adj. *tepidus*, moderately warm, lukewarm; L. pres. part. *amans*, loving; N.L. part. adj. *tepidamans*, loving warm conditions).

Basonym: *Geobacillus tepidamans* Schäffer *et al*. 2004.

The description of this species is as provided by Schäffer *et al.* [71] for *Geobacillus tepidamans*.

The type strain is ATCC BAA-942^T^=DSM 16325^T^=GS5-97^T^=R-35643^T^.

## Description of *Anoxybacteroides voinovskiensis* comb. nov.

*Anoxybacteroides voinovskiensis* (voi.novs.ki.en’sis. N.L. masc. adj. *voinovskiensis*, from Voinovskie, referring to the Voinovskie Hot Springs, the place of isolation).

Basonym: *Anoxybacillus voinovskiensis* Yumoto *et al*. 2004.

The description of this species is as provided by Yumoto *et al.* [72] for *Anoxybacillus voinovskiensis*.

The type strain is DSM 17075^T^=JCM 12111^T^=NCIMB 13956^T^=TH13^T^.

## Description of *Paranoxybacillus* gen. nov.

*Paranoxybacillus*(Par.an.o.xy.ba.cil’lus. Gr. prep. *para*, beside, near, next to; N.L. masc. n. *Anoxybacillus*, a bacterial genus; N.L. masc. n. *Paranoxybacillus*, beside *Anoxybacillus*).

Species are Gram-stain-positive, motile, rod-shaped, spore-forming. Spores are located terminally or sub-terminally. These species are facultatively anaerobic or obligately aerobic, catalase-variable and oxidase-positive. Species have been isolated from hot springs and soil samples. The members of this genus are moderately thermophilic, growing at temperatures ranging from 35 to 70 °C (optimal between 55 and 60 °C), a pH of 6.0–9.3 (optimal between pH 7.0 and 8.5) and NaCl concentration up to 4.0%. Their genome size ranges from 3.4 to 3.6 Mb and G+C content varies between 37.8 and 39.2 mol%. Species from this genus form a distinct clade in phylogenetic trees based on concatenated sequences of large numbers of proteins and in trees based on 16S rRNA gene sequences. Members of this genus can also be distinguished from all other *Anoxybacillaceae* genera based on three CSIs described in this work (Table 1), which are generally exclusively found in these species. The CSIs specific to this genus are present in the following proteins: excinuclease ABC subunit UvrC, cytochrome c oxidase subunit II and rod shape-determining protein MreC.

The type species is *Paranoxybacillus vitaminiphilus.*

## Description of *Paranoxybacillus vitaminiphilus* comb. nov.

*Paranoxybacillus vitaminiphilud* (vi.ta.mi.ni’phi.lus. N.L. neut. n. *vitaminum*, vitamin; Gr. masc. adj. *philos*, friend, loving; N.L. masc. adj. *vitaminiphilus*, vitamin-loving, referring to the vitamin requirements).

Basonym: *Anoxybacillus vitaminiphilus* Zhang *et al*. 2013.

The description of this species is as provided by Zhang *et al.* [12] for *Anoxybacillus vitaminiphilus*.

The type strain is CGMCC 1.8979^T^=JCM 16594^T^.

## Description of *Thermaerobacillus* gen. nov.

*Thermaerobacillus* (Therm.a.e.ro.ba.cil’lus. Gr. masc. adj. *thermos*, hot; Gr. masc. n. *aêr*, air; L. masc. n. *bacillus*, a rod; N.L. masc. n. *Thermaerobacillus*, a rod which grows at high temperatures and in the presence of air).

The description of this genus in terms of its growth, morphological, biochemical and chemotaxonomic characteristics is identical to that given by Coorevits *et al.* [5] for *Anoxybacillus caldiproteolyticus.* This species branches distinctly from all other *Anoxybacillaceae* species in phylogenetic trees based on 16S rRNA gene sequences and large datasets of conserved proteins. Members of this genus can also be distinguished from all other *Anoxybacillaceae* genera based on two CSIs described in this work (Table 1), which are generally exclusively found in these species. The CSIs specific to this genus are present in the following proteins: heavy metal translocating P-type ATPase and DUF2225 domain-containing protein.

The type species is *Thermaerobacillus caldiproteolyticus.*

## Description of *Thermaerobacillus caldiproteolyticus* comb. nov.

*Thermaerobacillus caldiproteolyticus* (cal.di.pro.te.o.ly’ti.cus. L. masc. adj. *caldus*, hot; N.L. masc. adj. *proteolyticus*, proteolytic; N.L. masc. adj. *caldiproteolyticus*, hot and protein degrading).

Basonym: *Anoxybacillus caldiproteolyticus* Coorevits *et al.* 2012.

The description of this species is the same as described by Coorevits *et al.* [5] for *Anoxybacillus caldiproteolyticus*.

The type strain is ATCC BAA-818^T^=DSM 15730^T^=LMG 26209^T^=R-35652=SF03^T^.

## Description of *Brevibacillus sedimenti* nom. nov.

*Brevibacillus sedimenti* (se.di.men’ti. L. gen. n. *sedimenti*, of sediment).

Basonym: *Anoxybacillus sediminis* Khan *et al.* 2019 (the name *Brevibacillus sediminis* has already been validly published [73], hence a new epithet has been chosen to avoid homonyms).

The description of this species is the same as described by Khan *et al.* [13] for *Anoxybacillus sediminis.*

The type strain is DSM 103835^T^=KCTC 33884^T^=YIM 73012^T^.

## Other taxonomic considerations (synonyms)

Results presented here show that the *Parageobacillus caldoxylosilyticus* [20], which was originally described as *Saccharococcus caldoxylosilyticus* [51], branches with the type species of the genus *Saccharococcus*, *S. thermophilus* [22]. Hence, we are proposing that this species should be known by its original validly published name i.e. *Saccharococcus caldoxylosilyticus* and *Parageobacillus caldoxylosilyticus* should be considered as a later homotypic synonym of this name.

## supplementary material

10.1099/ijsem.0.006528 Uncited Supplementary Material 1.

## Abbreviations

aa: amino acid
AAI: average amino acid identity
CSI: conserved signature indel
GTDB: genome taxonomy database
ML: maximum-likelihood
SH: Shimodaira–Hasegawa
