# Supplementary material for: Phylogenomic and molecular marker based studies to clarify the evolutionary relationships amongst Anoxybacillus species and demarcation of the family Anoxybacillaceae and some of its constituent genera
Source: Int J Syst Evol Microbiol. 2024 Sep 17;74(9):006528. doi: 10.1099/ijsem.0.006528 (PMC11407518; doi:10.1099/ijsem.0.006528)
Supplement: Uncited Supplementary Material 1. [file ijsem-74-06528-s001.pdf]

Table S1- Pairwise Average Amino acid Identity (AAI) values for Anoxybacillaceae species

	Anoxybacillus ayderensis	Anoxybacillus flavithermus	Anoxybacillus gonensis	Anoxybacillus kestanbolensis	Anoxybacillus mongoliensis	Anoxybacillus pushchinoensis ^{T}	Anoxybacillus suryakundensis	Anoxybacillus tengchongensis	Anoxybacillus thermarum	Thermolongibacillus altinsuensis ^{T}	Anoxybacillus amylolyticus	Anoxybacillus voinovskiensis	Anoxybacillus tepidamans	Anoxybacillus rupiensis	Anoxybacillus caldiproteolyticus	Geobacillus icigianus	Geobacillus jurassicus	Geobacillus kaustophilus
Anoxybacillus ayderensis	1.00	0.93	0.96	0.92	0.92	0.91	0.92	0.92	0.96	0.78	0.73	0.74	0.74	0.71	0.72	0.69	0.69	0.69
Anoxybacillus flavithermus	0.93	1.00	0.93	0.97	0.94	0.91	0.93	0.93	0.92	0.78	0.73	0.73	0.73	0.71	0.72	0.69	0.69	0.69
Anoxybacillus gonensis	0.96	0.93	1.00	0.92	0.92	0.90	0.92	0.92	0.95	0.79	0.73	0.73	0.73	0.71	0.72	0.69	0.69	0.69
Anoxybacillus kestanbolensis	0.92	0.97	0.92	1.00	0.93	0.91	0.93	0.93	0.92	0.78	0.73	0.74	0.73	0.71	0.72	0.69	0.69	0.69
Anoxybacillus mongoliensis	0.92	0.94	0.92	0.93	1.00	0.91	0.96	0.96	0.92	[illegible]	[illegible]	[illegible]	[illegible]	0.71	0.72	0.68	0.69	0.69
Anoxybacillus pushchinoensis ^{T}	0.91	0.91	0.90	0.91	0.91	1.00	0.91	0.90	0.91	0.79	0.73	0.73	0.73	0.71	0.72	0.69	0.70	0.69
Anoxybacillus suryakundensis	0.92	0.93	0.92	0.93	0.96	0.91	1.00	0.97	0.92	0.78	0.73	0.73	0.73	0.71	0.72	0.69	0.69	0.69
Anoxybacillus tengchongensis	0.92	0.93	0.92	0.93	0.96	0.90	0.97	1.00	0.91	0.78	0.73	0.73	0.73	0.71	0.72	0.68	0.69	0.68
Anoxybacillus thermarum	0.96	0.92	0.95	0.92	0.92	0.91	0.92	0.91	1.00	0.78	0.73	[illegible]	[illegible]	0.71	0.72	0.69	0.69	0.69
Thermolongibacillus altinsuensis ^{T}	0.78	0.78	0.79	0.78	0.78	0.79	0.78	0.78	0.78	1.00	0.74	0.74	0.75	0.73	0.76	0.70	0.70	0.70
Anoxybacillus amylolyticus	0.73	0.73	0.73	0.73	0.73	0.73	0.73	0.73	0.73	0.74	1.00	0.93	0.85	0.79	0.79	0.72	0.72	0.72
Anoxybacillus voinovskiensis	0.74	0.73	0.73	0.74	0.74	0.73	0.73	0.73	0.73	0.74	0.93	1.00	0.85	0.79	0.78	[illegible]	[illegible]	0.72
Anoxybacillus tepidamans	0.74	0.73	0.73	0.73	0.73	0.73	0.73	0.73	0.73	0.75	0.85	0.85	1.00	0.81	0.81	0.72	0.73	0.72
Anoxybacillus rupiensis	0.71	0.71	0.71	0.71	0.71	0.71	0.71	0.71	0.71	0.73	0.79	0.79	0.81	1.00	0.78	0.71	0.71	0.71
Anoxybacillus caldiproteolyticus	0.72	0.72	0.72	0.72	0.72	0.72	0.72	0.72	0.72	0.76	0.79	0.78	0.81	0.78	1.00	0.72	[illegible]	[illegible]
Geobacillus icigianus	0.69	0.69	0.69	0.69	0.68	0.69	0.69	0.68	0.69	0.70	0.72	0.72	0.72	0.71	0.72	1.00	0.87	0.88
Geobacillus jurassicus	0.69	0.69	0.69	0.69	0.69	0.70	0.69	0.69	0.69	0.70	0.72	0.72	0.73	0.71	0.73	0.87	1.00	0.92
Geobacillus kaustophilus	0.69	0.69	0.69	0.69	0.69	0.69	0.69	0.68	0.69	0.70	0.72	0.72	0.72	0.71	0.73	0.88	0.92	1.00
Geobacillus proteiniphilus	0.69	0.69	0.69	0.69	0.69	0.70	0.69	0.68	0.69	0.70	0.72	0.72	0.73	0.71	0.73	0.87	0.93	0.96
Geobacillus stearothermophilus ^{T}	0.69	0.69	0.69	0.69	0.69	0.70	0.69	0.69	0.69	0.70	0.72	0.72	0.73	0.71	0.73	0.87	0.89	0.91
Geobacillus subterraneus	0.69	0.69	0.69	0.69	0.69	0.70	0.69	0.69	0.70	0.70	0.72	0.72	0.73	0.71	0.73	0.87	0.89	0.89
Geobacillus thermocatenulatus	0.69	0.69	0.69	0.69	0.69	0.69	0.69	0.69	0.69	0.70	0.72	0.72	0.72	0.71	0.73	0.87	0.92	0.95
Geobacillus thermodenitrificans	0.69	0.69	0.69	0.69	0.69	0.70	0.69	0.69	0.69	0.70	0.72	0.72	0.72	0.71	0.72	0.86	0.87	0.87
Geobacillus thermoleovorans	0.69	0.69	0.69	0.69	0.69	0.70	0.69	0.68	0.69	0.70	0.72	0.72	0.72	0.71	0.73	0.88	0.92	0.98
Geobacillus uzenensis	0.69	0.69	0.69	0.69	0.69	0.70	0.69	0.69	0.69	0.70	0.72	0.72	0.73	0.71	0.73	0.87	0.88	0.89
Geobacillus vulcani	0.69	0.69	0.69	0.69	0.69	0.70	0.69	0.69	0.69	0.70	0.72	0.72	0.72	0.71	0.73	0.90	0.91	0.93
Geobacillus zalihae	0.69	0.69	0.69	0.69	0.69	0.70	0.69	0.68	0.69	0.70	0.72	0.72	0.72	0.71	0.73	0.87	0.92	0.95
Saccharococcus thermophilus ^{T}	0.71	0.71	0.71	0.71	0.71	0.72	0.71	0.71	0.71	0.74	0.77	0.77	0.78	0.76	0.80	0.77	0.78	0.77
Parageobacillus caldoxylosilyticus	0.71	0.71	0.71	0.71	0.70	0.71	0.70	0.70	0.71	0.73	0.76	0.75	0.77	0.75	0.79	0.76	0.77	0.77
Parageobacillus galactosidasius	0.71	0.71	0.71	0.71	0.71	0.72	0.71	0.71	0.71	0.74	0.77	0.76	0.78	0.76	0.80	0.75	0.76	0.76
Parageobacillus thermantarcticus	0.71	0.71	0.71	0.71	0.71	0.72	0.71	0.71	0.71	0.74	0.77	0.76	0.78	0.76	0.80	0.75	0.77	0.77
Parageobacillus thermoglucosidasius ^{T}	0.71	0.71	0.71	0.71	0.71	0.71	0.71	0.70	0.71	0.74	0.76	0.76	0.77	0.75	0.80	0.75	0.76	0.75
Parageobacillus toebii	0.71	0.72	0.71	0.71	0.71	0.72	0.71	0.71	0.71	0.74	0.77	0.76	0.78	0.76	0.80	0.75	0.76	0.76
Anoxybacillus calidus	0.73	0.73	0.73	0.73	0.73	0.73	0.73	0.72	0.73	0.77	0.76	0.76	0.77	0.75	0.80	0.70	0.71	0.70
Anoxybacillus vitaminiphilus	0.73	0.73	0.73	0.73	0.72	0.73	0.73	0.72	0.72	0.77	0.76	0.75	0.78	0.75	0.79	0.70	0.71	0.70
Anoxybacillus sediminis	0.59	0.59	0.59	0.59	0.59	0.59	0.59	0.59	0.59	0.59	0.59	0.58	0.58	0.58	0.58	0.58	0.58	0.58

	Geobacillus proteiniphilus	Geobacillus stearothermophilus ^{T}	Geobacillus subterraneus	Geobacillus thermocatenulatus	Geobacillus thermodenitrificans	Geobacillus thermoleovorans	Geobacillus uzenensis	Geobacillus vulcani	Geobacillus zalihae	Saccharococcus thermophilus ^{T}	Parageobacillus caldoxylosilyticus	Parageobacillus galactosidasius	Parageobacillus thermantarcticus	Parageobacillus thermoglucosidasius ^{T}	Parageobacillus toebii	Anoxybacillus calidus	Anoxybacillus vitaminiphilus	Anoxybacillus sediminis
Anoxybacillus ayderensis	0.69	0.69	0.69	0.69	0.69	0.69	0.69	0.69	0.69	0.71	0.71	0.71	0.71	0.71	0.71	0.73	0.73	0.59
Anoxybacillus flavithermus	0.69	0.69	0.69	0.69	0.69	0.69	0.69	0.69	0.69	0.71	0.71	0.71	0.71	0.71	0.72	0.73	0.73	0.59
Anoxybacillus gonensis	0.69	0.69	0.69	0.69	0.69	0.69	0.69	0.69	0.69	0.71	0.71	0.71	0.71	0.71	0.71	0.73	0.73	0.59
Anoxybacillus kestanbolensis	0.69	0.69	0.69	0.69	0.69	0.69	0.69	0.69	0.69	0.71	0.71	0.71	0.71	0.71	0.71	0.73	0.73	0.59
Anoxybacillus mongoliensis	0.69	0.69	0.69	0.69	0.69	0.69	0.69	0.69	0.69	0.71	0.70	0.71	0.71	0.71	0.71	0.73	0.72	0.59
Anoxybacillus pushchinoensis ^{T}	0.70	0.70	0.70	0.69	0.70	0.70	0.70	0.70	0.70	0.72	0.71	0.72	0.72	0.71	0.72	0.73	0.73	0.59
Anoxybacillus suryakundensis	0.69	0.69	0.69	0.69	0.69	0.69	0.69	0.69	0.69	0.71	0.70	0.71	0.71	0.71	0.71	0.73	0.73	0.59
Anoxybacillus tengchongensis	0.68	0.69	0.69	0.69	0.68	0.69	0.69	0.69	0.68	0.71	0.70	0.71	0.71	0.70	0.71	0.72	0.72	0.59
Anoxybacillus thermarum	0.69	0.69	0.70	0.69	0.69	0.69	0.69	0.69	0.69	0.71	0.71	0.71	0.71	0.71	0.71	0.73	0.72	0.59
Thermolongibacillus altinsuensis ^{T}	0.70	0.70	0.70	0.70	0.70	0.70	0.70	0.70	0.70	0.74	0.73	0.74	0.74	0.74	0.74	0.77	0.77	0.59
Anoxybacillus amylolyticus	0.72	0.72	0.72	0.72	0.72	0.72	0.72	0.72	0.72	0.77	0.76	0.77	0.77	0.76	0.77	0.76	0.76	0.59
Anoxybacillus voinovskiensis	0.72	0.72	0.72	0.72	0.72	0.72	0.72	0.72	0.72	0.77	0.75	0.76	0.76	0.76	0.76	0.76	0.75	0.58
Anoxybacillus tepidamans	0.73	0.73	0.73	0.72	0.72	0.72	0.73	0.72	0.72	0.78	0.77	0.78	0.78	0.77	0.78	0.77	0.78	0.58
Anoxybacillus rupiensis	0.71	0.71	0.71	0.71	0.71	0.71	0.71	0.71	0.71	0.76	0.75	0.76	0.76	0.75	0.76	0.75	0.75	0.58
Anoxybacillus caldiproteolyticus	0.73	0.73	0.73	0.73	0.72	0.73	0.73	0.73	0.73	0.80	0.79	0.80	0.80	0.80	0.80	0.80	0.79	0.58
Geobacillus icigianus	0.87	0.87	0.87	0.87	0.86	0.88	0.87	0.90	0.87	0.77	0.76	0.75	0.75	0.75	0.75	0.70	0.70	0.58
Geobacillus jurassicus	0.93	0.89	0.89	0.92	0.87	0.92	0.88	0.91	0.92	0.78	0.77	0.76	0.77	0.76	0.76	0.71	0.71	0.58
Geobacillus kaustophilus	0.96	0.91	0.89	0.95	0.87	0.98	0.89	0.93	0.95	0.77	0.77	0.76	0.77	0.75	0.76	0.70	0.70	0.58
Geobacillus proteiniphilus	1.00	0.90	0.89	0.94	0.87	0.96	0.88	0.92	0.94	0.78	0.77	0.76	0.77	0.76	0.76	0.71	0.71	0.58
Geobacillus stearothermophilus ^{T}	0.90	1.00	0.89	0.91	0.87	0.91	0.89	0.89	0.92	0.78	0.77	0.76	0.76	0.76	0.76	0.71	0.71	0.59
Geobacillus subterraneus	0.89	0.89	1.00	0.88	0.91	0.89	0.98	0.88	0.88	[illegible]	[illegible]	0.76	0.76	0.75	0.76	0.71	0.71	0.58
Geobacillus thermocatenulatus	0.94	0.91	0.88	1.00	0.87	0.95	0.88	0.92	0.95	0.77	0.77	0.76	0.76	0.75	0.76	0.71	0.70	0.58
Geobacillus thermodenitrificans	0.87	0.87	0.91	0.87	1.00	0.87	0.91	0.87	0.86	0.77	0.77	0.76	0.77	0.75	0.76	0.71	0.71	0.58
Geobacillus thermoleovorans	0.96	0.91	0.89	0.95	0.87	1.00	0.89	0.93	0.95	0.77	0.76	0.76	0.76	0.75	0.76	0.71	0.71	0.58
Geobacillus uzenensis	0.88	0.89	0.98	0.88	0.91	0.89	1.00	0.88	0.88	0.78	0.77	0.76	0.76	0.75	0.76	0.71	0.71	0.59
Geobacillus vulcani	0.92	0.89	0.88	0.92	0.87	0.93	0.88	1.00	0.92	0.77	0.77	0.76	0.76	0.75	0.76	0.71	0.71	0.58
Geobacillus zalihae	0.94	0.92	0.88	0.95	0.86	0.95	0.88	0.92	1.00	0.77	0.76	0.76	0.77	0.75	0.76	0.70	0.71	0.58
Saccharococcus thermophilus ^{T}	0.78	0.78	0.77	0.77	0.77	0.77	0.78	0.77	0.77	1.00	0.92	0.87	[illegible]	[illegible]	0.88	0.76	0.76	0.59
Parageobacillus caldoxylosilyticus	0.77	0.77	0.77	0.77	0.77	0.76	0.77	0.77	0.76	0.92	1.00	0.87	0.86	0.85	0.87	0.75	0.75	0.58
Parageobacillus galactosidasius	0.76	0.76	0.76	0.76	0.76	0.76	0.76	0.76	0.76	0.87	0.87	1.00	0.88	0.88	0.98	0.76	0.76	0.58
Parageobacillus thermantarcticus	0.77	0.76	0.76	0.76	0.77	0.76	0.76	0.76	0.77	0.87	0.86	0.88	1.00	0.92	0.89	[illegible]	[illegible]	[illegible]
Parageobacillus thermoglucosidasius ^{T}	0.76	0.76	0.75	0.75	0.75	0.75	0.75	0.75	0.75	0.86	0.85	0.88	0.92	1.00	0.88	0.76	0.76	0.58
Parageobacillus toebii	0.76	0.76	0.76	0.76	0.76	0.76	0.76	0.76	0.76	0.88	0.87	0.98	0.89	0.88	1.00	0.77	0.76	0.59
Anoxybacillus calidus	0.71	0.71	0.71	0.71	0.71	0.71	0.71	0.71	0.70	0.76	0.75	0.76	0.76	0.76	0.77	1.00	0.90	[illegible]
Anoxybacillus vitaminiphilus	0.71	0.71	0.71	0.70	0.71	0.71	0.71	0.71	0.71	0.76	0.75	0.76	0.76	0.76	0.76	0.90	1.00	[illegible]
Anoxybacillus sediminis	0.58	0.59	0.58	0.58	0.58	0.58	0.59	0.58	0.58	0.59	0.58	0.58	0.59	0.58	0.59	0.58	0.59	1.00

0.58	0.59	0.60	0.61	0.62	0.64	0.65	0.66	0.67	0.68	0.70	0.71	0.72	0.73	0.74	0.76	0.77	0.78	
0.79	0.80	0.82	0.83	0.84	0.85	0.86	0.88	0.89	0.90	0.91	0.92	0.94	0.95	0.96	0.97	0.98	1.00	

Anoxybacillus sensu stricto

g_Thermolongibacillus

g_Anoxybacillus_A

g_Anoxybacillus_C

g_Geobacillus

g_Saccharococcus

g_Parageobacillus

g_Anoxybacillus_B

A. sediminis

Group	Species	Accession	282	Insert	322
Anoxybacillus sensu stricto (9/ 9)	*Anoxybacillus pushchinoensis*	WP_091700255	FQRIVRAIKEEPKKTIKNIVKALL	PL	PERYILFLLKQVAID
	Anoxybacillus mongoliensis	WP_183241919	-------V--Q------T----T-	--	----V----E-AT--
	Anoxybacillus flavithermus	WP_064214292	--H----M---------T----A-	--	----V----E-AT--
	Anoxybacillus tengchongensis	WP_183249150	-------M---------T----TI	S-	----V----E-AT--
	Anoxybacillus suryakundensis	WP_055440185	-------M---------T----T-	S-	----V----E-AT--
	Anoxybacillus kestanbolensis	WP_077429244	-----HTM---------T---GTI	S-	----V----E-AT--
	Anoxybacillus ayderensis	MCL6616549	--H---TM------A-------IM	--	----V----E-TK--
	Anoxybacillus thermarum	WP_043966962	--Q---TM------A-------IM	--	----V----E-TK--
	Anoxybacillus gonensis	WP_035065289	--Q---TM------V----I--II	--	---------E-TK--
Other *Anoxybacillaceae* (0/ >15)	*Anoxybacillus caldiproteolyticus*	WP_194520700	--S--SRL-A----AV--V--GI-		----L----EKNE--
	Anoxybacillus tepidamans	WP_183253169	----ITLM-A----A---VL-G--		----L----EKNG--
	Anoxybacillus calidus	WP_181537612	--E--KLL-A----AV--VL-G--		----L----EKND--
	Anoxybacillus vitaminiphilus	WP_111644444	--E--KLL-A----AV--VL-G--		----L----EKND--
	Anoxybacillus rupiensis	MBS2771474	---V-A---N----A---LL-G--		----L----EKNH--
	Anoxybacillus voinovskiensis	MBB4074024	---LLHL-------AV--VL-G--		----L----E-NG--
	Anoxybacillus amylolyticus	ANB62143	----IHLF-T----AV--VL-G--		----L----EKNE--
	Geobacillus jurassicus	WP_066234347	--HFAKLC------AM-T-A-TV-		----AV---ERAG--
	Geobacillus kaustophilus	WP_044736815	--HFAKLC------AM-T-A-TV-		----AV---ERAG--
	Geobacillus stearothermophilus	KYD21459	--QFAKLC------AM-T-T-TV-		----AV---ERAG--
	Geobacillus thermoleovorans	WP_068895830	--HFAKLC------AM-T-A-TV-		----AV---ERAG--
	Geobacillus zalihae	WP_081212687	--QFAKLC------AM-T-A-TV-		----AV---ERAG--
	Geobacillus thermodenitrificans	WP_029760580	---LAKLC-Q----AV---A-TV-		----AA---ERSG--
	Parageobacillus thermoglucosidasius	WP_064549510	--QLTN-C-T----A---VI-G--		---------ERSN--
	Parageobacillus thermantarcticus	WP_090947688	--Y-AHLC------A---VL-V--		----V----ERSN--
	Parageobacillus caldoxylosilyticus	WP_017434908	--H-AHLC------AV--VL-G--		----V----E-SG--
	Parageobacillus toebii	WP_062755032	--H-AHLC------A---VL-G--		---------E-SH--
	Parageobacillus galactosidasius	WP_089098028	--H-AHLC------A---VL-G--		---------E-SH--
	Saccharococcus thermophilus	NIK15800	--QLAN-C-A----A---V--G--		----V----E-SG--
	Thermolongibacillus altinsuensis	WP_243643117	--S--NML-A----A---VL-G--		----L----EKNG--
Other Bacteria (0/ >40)	*Siminovitchia fortis*	WP_120073221	--K-QK-M------A---SL-G--		----L----E-SD--
	Bacillus alveayuensis	WP_044894456	--E--KLL-A----AV--VL-G--		----L----EKNE-E
	Neobacillus thermocopriae	WP_163251160	--E--KLV-A----NL---L-G--		----L----EKNE--
	Neobacillus niacini	WP_262306360	--E--KLV------S---TL-G--		----L----D-ND--
	Bacillus sinesaloumensis	WP_077619132	--Q-LKLA------A----L-GF-		----L----EKN---
	Bacillus ectoiniformans	WP_204556576	--KLNQIM--H----V--SL-G--		----L----E-AN--
	Neobacillus mesonae	WP_251471371	--E--KLV-A----S---TL-G--		----L----E-TE--
	Priestia abyssalis	WP_078414082	--SMIKML------A----C-G--		----L----E-AD--
	Bacillus timonensis	WP_010678481	--Q-LKLA--D---AL-----GF-		----L----EKNE--
	Bacillus dafuensis	WP_057772204	--E-NKQL------S---LL-GI-		----L----E-SD--
	Fredinandcohnia onubensis	WP_099362226	--Q-LKLV------AL---L-GF-		----L----ERNE--
	Cytobacillus oceanisediminis	WP_243505471	--EVSKK---D---S---LL-GM-		----L----E-NG--
	Neobacillus novalis	WP_066096852	--E---LV-S----S---TL-G--		----L----E-NG--
	Bacillus suaedaesalsae	WP_204201769	--Q--KLL-D--N-S---VL-S--		----L----E-CE--
	Cytobacillus firmus	RBP93791	--DVSKK-------S---LL-GM-		----L----E-NG--
	Aeromonas veronii	WP_258722544	--Q-LKLM------AL---L-GF-		----L----ERNE--
	Mesobacillus campisalis	WP_046523517	--E-SKL--A----AV--VL-G--		----L----E-LD--
	Priestia megaterium	WP_221787867	--KL-KTT------A---VL-GFV		S---L----E-AS--
	Fredinandcohnia humi	WP_057999369	--Q-LKQA-D----AL---L-GV-		----L----EKNE--
	Bacillus dielmonensis	WP_042459827	Y-NL-KLV-S----S---TL-G--		----L----E-SE--
	Sporosarcina globispora	WP_053434049	--DVSKK---D---S---LL-GM-		----L----E-NG--
	Priestia koreensis	WP_251605693	--Q--KLF------AS--VL-GF-		----L----E-AD--
	Caldibacillus hisashii	WP_216406184	--E--SHV--K---AV--VL-G--		----L----E-AE--
	Neobacillus sedimentimangrovi	WP_231314996	--E--KLV-A----NL---L-G--		----L----D-NE--
	Bacillus xiapuensis	WP_100331894	--E-C-LV-NH---SL--CL-SF-		----V----E-AE--
	Litchfieldia salsa	WP_090849287	--S-IKMA------AL--LL-GF-		----L----EKNT--
	Cytobacillus eiseniae	WP_066396926	--E-SKVM-DD---S---LL-GY-		----L----E-NQ--
	Cytobacillus horneckiae	WP_066192052	--EMMKM--D----S----I-GIV		----L----EKND--
	Neobacillus pocheonensis	MCM2531368	--E--KLV-N----I---TL-GI-		----L----E-ND--
	Cytobacillus solani	WP_053477375	L-E-YKL--D----S---LL-GF-		----L----E-CE--
	Bacillus freudenreichii	VEF47220	--KVQKLV------A---AL-G--		---FL--M-E-SE--
	Siminovitchia fordii	WP_018707404	--KLKKTM-D----A---SL-G--		----L----E-SN--
	Cytobacillus gottheilii	WP_066442948	--DT--KM------S---TL-GFI		----L----EKSE--
	Ectobacillus funiculus	WP_129727871	----IQQ--N----G---VL-GHV		----L----EKNN--
	Heyndrickxia sporothermodurans	GIN88165	--QVIKLV------A---SF-G--		----L----ETNRM-
	Cytobacillus depressus	WP_151533632	--E-LSG--A----S---LL-GH-		----L----ERSG--
	Priestia aryabhattai	WP_221817827	--QL-KTT------A---VL-GFV		S---L----E-AD--
	Bacillus pumilus	WP_144557313	--KLH-D-------A---VL-SWM		Q---L----ERNG--
	Bacillus rubiinfantis	WP_042354726	--Q-IKL--S----G---TL-G--		----L----A-NE--

Figure S1: Partial sequence alignment of the NAD(P)/FAD-dependent oxidoreductase protein showing a 2 aa insert (highlighted) in a conserved region that is exclusively shared by all species from the *Anoxybacillus sensu stricto*. This CSI is also not shared by any other species from the family *Anoxybacillaceae* or other bacteria.

Group	Species	Accession	101		147
Anoxybacillus sensu stricto (9/ 9)	*Anoxybacillus pushchinoensis*	WP_091701995	NVRLLHERYSIGNRKKIKNNMIAKTIL		GDPFFYPAQFVDSLSDVLRD
	Anoxybacillus flavithermus	WP_041638874	---------------------------		--------------------
	Anoxybacillus suryakundensis	WP_082435668	---------------------------		--------------------
	Anoxybacillus gonensis	AKS37483	----------------V----------		--------------------
	Anoxybacillus mongoliensis	WP_183240673	---------------------------		--------------------
	Anoxybacillus tengchongensis	WP_183248350	---------------------------		--------------------
	Anoxybacillus thermarum	WP_084221222	----------------V------R---		-------------------N
	Anoxybacillus ayderensis	MCL6617288	----------------V----------		--------------------
	Anoxybacillus kestanbolensis	OOE06365	---------------------------		--------------------
Other *Anoxybacillaceae* (0/ >15)	*Anoxybacillus voinovskiensis*	WP_183185411	-I--M-----LA----V-KQLV---V-	SS	-------T------------
	Anoxybacillus caldiproteolyticus	WP_199426290	-I--M-----------VQKDLV---V-	SS	-------T------G-----
	Anoxybacillus tepidamans	WP_183254815	----M-----L-----VQKQLVV--V-	SS	-------T------------
	Anoxybacillus amylolyticus	WP_066323164	-I--MY----LA----V-KQLV---V-	SS	-------T------------
	Anoxybacillus vitaminiphilus	WP_181502819	-I--M------------QK-LVVQ-V-	ST	----V--T------G-T---
	Anoxybacillus calidus	WP_220129196	-I--M------------QK-LVVQ-V-	ST	----I--T------G-T---
	Geobacillus stearothermophilus	ALA70927	-I--M-----L-----V-K-LVVQ-V-	SS	S------T------G---M-
	Geobacillus thermocatenulatus	WP_025951227	-I--M-----L-----V-K-LVVQ-V-	SS	S------T------G---I-
	Geobacillus thermoleovorans	WP_069304029	-I--M-----L-----V-K-LVVQ-V-	SS	S------T------G---I-
	Geobacillus kaustophilus	WP_044732206	-I--M-----L-----V-K-LVVQ-V-	SS	S------T------G---I-
	Geobacillus subterraneus	WP_172418676	-I--M-----L-----V-R-LVVQ-V-	SS	S------T------G---I-
	Geobacillus vulcani	WP_031405462	-I--M-----L-----V-K-LVVQ-V-	SS	S------T------G---M-
	Geobacillus jurassicus	WP_066231254	-I--M-----L-----V-K-LVVQ-V-	SS	S------T------G---I-
	Parageobacillus thermantarcticus	WP_090948660	-I--M------S----VQK-LV---V-	SS	-------T------G-I-I-
	Parageobacillus thermoglucosidasius	WP_003252568	-I--------------VQK-LV---V-	SS	-------T------G-I-I-
	Saccharococcus thermophilus	WP_208404554	-I--------L-----VQK-LVVR-V-	SS	-------T-----IG---M-
	Thermolongibacillus altinsuensis	WP_132947887	-I--------L------Q---V-----	TY	-------T------G-----
Other Bacteria (0/ >30)	*Bacillus carboniphilus*	WP_226539349	-I-II------------GSQL-SRML-	NS	---L---V---E------M-
	Bacillus bingmayongensis	WP_017153109	-I-IS---F-----EQNTP-L--R-V-	SS	T--L---V------G-T---
	Bacillus weihaiensis	WP_072581923	-I--T---------R-AS-LLS-S--	NT	S-SM---V------G-T---
	Bacillus suaedaesalsae	WP_204203150	TI-I------L---VNLN--IV--AVF	NS	---L---M---E--G-T---
	Bacillus cereus group	WP_016113279	-I-IS---F-----ERNTP-L--R-V-	SS	S--L---V------G-----
	Metabacillus litoralis	WP_146949530	-I--T------------AS-LLS-S--	NT	S-SM---V------G-T---
	Metabacillus crassostreae	WP_204954051	-I--T---------R-AS-LLS-S--	NT	S-SM---V------G-T---
	Bacillus pseudomycoides	WP_097849873	-I-IS---F-----ERNTP-L--R-V-	SS	T--L---V------G-----
	Bacillus alveayuensis	WP_200894644	-I--M-----------MQK-L-VR-A-	ST	----M--T------G-T---
	Metabacillus iocasae	WP_205185894	HI-I----------V-LQ--IV---V-	KT	-E-L---L------G-T---
	Streptococcus pneumoniae	CJG00450	H--I----F-----V-LP--IV--SV-	KT	-E-L---V------G-T---
	Priestia megaterium	RBN38239	H--I----F-----V-LP--IV--SV-	KT	-E-L---V------G-T---
	Priestia flexa	WP_251385466	HI-I----F-----V-LP--IV---M-	KT	-E-L---V------G-T---
	Priestia aryabhattai	WP_193420826	H--I----F-----V-LP--IV--SV-	KT	-E-L---V------G-T---
	Bacillus solimangrovi	WP_069717015	HI---N-------QI-R---L----V-	GT	---L---I--------N---
	Metabacillus schmidteae	WP_102228296	-I--T---F-------VAS-LLS-S--	NT	S-SM---V------G-T---
	Bacillus kexueae	WP_243385939	---II---F--------QT-LMS--L-	NT	A--L---I---E--G-T---
	Pseudomonas aeruginosa	KAA5620576	-I--M-----L-----V-K-LVVQ-V-	SS	S------T------G---I-
	Bacillus mesophilus	WP_163177753	-I--------L---VHLQ---V-RAVF	VT	---L---M---E--G-S---
	Bacillus cereus	PEE90943	---IS---F-----ERNTP-L--R-V-	SS	A--L---V------G-A---
	Metabacillus niabensis	WP_226529799	-I--T---F-------VSP-LLS-S--	NT	S-SM---V------G-T---
	Metabacillus halosaccharovorans	MBU7595031	-I--T---F-------VSP-LLS-S--	NT	S-SM---V------G-T---
	Ectobacillus funiculus	WP_129726592	-I-IC---F-L---RSLAS-L----V-	SS	A--V---V---E--G-T---
	Bacillus licheniformis	SPU04239	-I-I---K--------TNS-LLG--MI	HP	S--I---V--------M-M-
	Bacillus paralicheniformis	WP_145685695	-I-I---K--------TNS-LLG--MI	HP	S--I---V--------M-M-
	Bacillus mycoides	WP_215569865	---IS---------ERNTP-L--R-V-	SS	A--L---V------G-A---
	Aeribacillus pallidus	WP_094244621	-I-II---F--------SS-LVS-ML-	NS	S--L---I---E--A-A-I-
	Aeribacillus composti	WP_144597096	-I-II---F--------SS-LVS-ML-	NS	S--L---I---E--A-A-I-
	Peribacillus frigoritolerans	TDL80789	---IV---F-L-----VSS-IVG--L-	SP	T--L---V------GEN---
	Metabacillus idriensis	WP_191562443	---IV---F-L-----VSS-IVG--L-	SP	T--L---V------GEN---
	Bacillus thuringiensis	WP_153064387	---IS---F-----ERNTP-L--R-V-	SS	A--L---V------G-A---
	Bacillus nitratireducens	WP_242399637	---IS---------ERNTP-L--R-V-	SS	A--L---V------G-A---
	Bacillus toyonensis	WP_100063534	---IS---F-----ERNTP-L--R-V-	SS	A--L---V------G-A---
	Bacillus altitudinis	WP_235701704	-------KF-------TQSQLMG--LI	HP	S--I---V---E----M-M-

Figure S2: Partial sequence alignment of the Cof-type HAD-IIB family hydrolase protein showing a 2 aa deletion (highlighted) in a conserved region that is exclusively shared by species from the *Anoxybacillus sensu stricto*. This CSI is also not shared by any other species from the family *Anoxybacillaceae* or other bacteria.

Group	Species	Accession	59		96
Anoxybacillus sensu stricto (9/ 9)	*Anoxybacillus pushchinoensis*	WP_244149206	LVDMDDEQIVFQRYVDDLSPLTKA		GYFGLTKDGTLSIF
	Anoxybacillus suryakundensis	WP_032100194	--N---------------------		--------------
	Anoxybacillus tengchongensis	WP_246349006	--N---------------------		--------------
	Anoxybacillus mongoliensis	WP_246346224	-IN---------------------		--------------
	Anoxybacillus flavithermus	WP_230456161	-IN---------------------		--------------
	Anoxybacillus thermarum	WP_043965287	-IN---------------------		------E-------
	Anoxybacillus gonensis	WP_035067197	-IN---------------------		------E-------
	Anoxybacillus kestanbolensis	OOE05709	-IN---------------------		------E-------
	Anoxybacillus ayderensis	MCL6616183	-IN---------------------		------E-------
Other *Anoxybacillaceae* (0/ >15)	*Anoxybacillus calidus*	WP_246326842	------------KP-N-I---S--	N	--------------
	Anoxybacillus caldiproteolyticus	MBA2873631	------------KTIN-I---L--	N	-------N------
	Anoxybacillus tepidamans	WP_183253862	---L--S-----KK-N-I---L--	N	--------------
	Anoxybacillus voinovskiensis	WP_183183628	---L--K-----KTIN-I---A--	N	--------------
	Anoxybacillus amylolyticus	WP_066326434	---L--K-----KAIN-I---A--	N	--------------
	Anoxybacillus vitaminiphilus	WP_245934798	------------KS-N-I-A-S--	N	--------------
	Geobacillus subterraneus	WP_168368834	-ITL--RT---RKA-N-I---L--	N	----I-D-------
	Geobacillus kaustophilus	WP_044730718	--TL--QT---RKTIN-I---L-T	N	----I-D-------
	Geobacillus jurassicus	WP_066228720	--TL--QT---RKTIN-I---L-T	N	----I-D-------
	Geobacillus stearothermophilus	WP_121678076	-INL--RT-A-RKT-N-I---L-T	N	----I-D-------
	Geobacillus thermoleovorans	WP_069304420	--TL--QT---RKTIN-I---L-T	N	----I-D-------
	Parageobacillus thermoglucosidasius	WP_013876535	--N----TL--RKM---I---L--	N	----I-E-------
	Parageobacillus thermantarcticus	SFA38686	--N----TL--RKT-N-I---L-S	N	----I-E-------
	Parageobacillus toebii	WP_062677159	--N---QT---RKT-N-I---L--	N	----I-E-------
	Parageobacillus caldoxylosilyticus	MBB3850900	-IN---QT-I-RKT-N-I---L--	N	----I-E-------
	Saccharococcus thermophilus	WP_166910507	-IN---RT---RKT-N-I---L--	N	----I-E-------
	Thermolongibacillus altinsuensis	TCL51957	--N---------KEMN-I---A--	N	----I-E-------
Other Bacteria (0/ >50)	*Bacillus alveayuensis*	WP_044894833	------------KV-N-I---S--	N	--------------
	Sutcliffiella halmapala	WP_078379550	VI-L-E--V---Q----I---L-T	N	-----SG--V-T--
	Bacillus timonensis	WP_010678270	--HQ-E--V---QRI--I---L-T	N	------E--I----
	Margalitia camelliae	WP_101354185	--E---A-L---KQ---I---L-S	N	----ISN-------
	Metabacillus fastidiosus	WP_082799969	II-Q-E------KQ---I---L--	N	----IAN----T--
	Rossellomorea marisflavi	WP_148795575	-I--SE------KQM--I---L-T	N	------EE-V----
	Sutcliffiella rhizosphaerae	WP_230499407	VI-L-E--V---Q-I--I---L-S	N	----ISN--I-T-Y
	Bacillus tianshenii	WP_204416082	VI-LNE------Q-I--I---L-T	N	-----SN--V-T-Y
	Cytobacillus luteolus	WP_193535578	--HQ-E------QQI--I---L-S	N	----I-D--I-T-Y
	Fredinandcohnia onubensis	WP_099362042	--HQ--T-V---QRI--I---L-T	N	------E--I----
	Cytobacillus suaedae	QOR65670	--HQ-E------QQI--I---L-S	N	----I-EE-I-T-Y
	Sutcliffiella horikoshii	WP_148987757	VI-L-G--V---Q-I--I---L-S	N	----I-D--I-T-Y
	Rossellomorea aquimaris	WP_231893407	-I--TE-E-I--KR---I---L--	N	----ISEN-V----
	Metabacillus lacus	WP_246188267	--HQ-E------QK---I---L-S	N	----I-EN-I----
	Bacillus sinesaloumensis	WP_077621034	--HQ-ED-V---QHI--I---L-T	N	------E--I-T--
	Fredinandcohnia humi	WP_057998634	--HQ-ED-V---QR---I---L-T	N	------E--I----
	Bacillus norwichensis	WP_191816320	-----E-RLL-EKK---I---L-M	N	----I-D-------
	Siminovitchia terrae	WP_120116224	-----E-RLL-EKK---I---L-M	N	----I-D-------
	Heyndrickxia sporothermodurans	WP_108070388	I-Q--E----L-KKIN-I---L--	N	----I-D---I---
	Siminovitchia fortis	WP_120072758	-----EDRLI-KKK---I---L-M	N	----I-N-------
	Falsibacillus albus	WP_121678772	----E-GKVI---K-N-I---L--	N	----VS-----A--
	Priestia taiwanensis	WP_188388969	---Y-----TL-K-I--I--II-T	S	-----GD----K--
	Margalitia shackletonii	WP_055739151	--E--ED-L---TQ---I---L-S	N	----ISS-------
	Bacillus kexueae	WP_243386192	-ITQNEDE-I--K-I--I---L--	N	----IQE-------
	Siminovitchia acidinfaciens	WP_126048450	-----EDRLL-EKKI--I---L-M	N	----I-E-------
	Bacillus freudenreichii	VEF47489	-----EDRLL-EKKI--I---L-M	N	----I-E-------
	Sutcliffiella deserti	WP_223702884	VI-L-E--V---QQI--I---L-T	N	-----SN--V-T-Y
	Heyndrickxia vini	WP_202779929	I-Q--E----L-KKIN-I---L--	N	----ISD---I---
	Bacillus suaedaesalsae	MBM6619726	-I-Q-E--M---KNI--I---L--	N	----I-EN-V-T--
	Anaerobacillus isosaccharinicu	MBA5585348	-I-Q-EA-V---QR---I---L-I	N	----ISQE---N-Y
	Caldibacillus lycopersici	WP_263073233	---INADHV-LKK----I---L--	N	------EN------
	Cytobacillus horneckiae	WP_066191734	-I--NK--V--R-HM--I---L--	N	----I-DE-V-T--
	Bacillus enclensis	WP_098151194	-I--TE-EVI--KQ---I---L-T	N	----ISEN-V----
	Litchfieldia alkalitelluris	WP_078545381	MIHS-E--V---QH---I---L-S	N	----ISE--V-T--
	Caldibacillus thermoamylovoran	WP_051989113	--HV-GNR---KK----I---L-S	N	-F--V-D-------
	Cytobacillus firmus	WP_174750003	-----IDYM--R-K---I---L--	N	----V-E----T--
	Sutcliffiella cohnii	WP_066420849	--TQ-E--V---MHI--I---L-S	N	----I-EE-I-T--
	Bacillus fonticola	WP_170007795	-QE--QGH----KR-N-I---L--	N	----V--E------
	Metabacillus indicus	WP_051865408	---Q--N--I--KE---I---L--	N	-----SSE-VF---
	Bacillus haikouensis	WP_172252372	-I--TE-EVI--KQ---I---L-T	N	----VSEN-V----
	Siminovitchia fordii	WP_018708255	-----EDRLI-EKKI--I---L-M	N	----I-D-----L-
	Rossellomorea arthrocnemi	WP_201713617	-M--TE------KQM--I---L-T	N	----ISDN-V----
	Bacillus mesophilus	WP_163179203	-I-Q-EQL----KQI--I---L--	N	------SN---T--
	Cytobacillus oceanisediminis	WP_217032818	-----MDY---R-KM--I---L--	N	----V-E----T--

Figure S3: Partial sequence alignment of the intercompartmental signaling factor BofC protein showing a 1 aa deletion (highlighted) in a conserved region that is exclusively shared by species from the *Anoxybacillus sensu stricto*. This CSI is also not shared by any other species from the family *Anoxybacillaceae* or other bacteria.

Group	Species	Accession	59		100
Anoxybacillus sensu stricto (9/ 9)	*Anoxybacillus pushchinoensis*	WP_091704058	GATRLAVAFLDEALALRKKGI	PL	DVPILVLGATNPQYAPLAA
	Anoxybacillus tengchongensis	WP_183250224	---H----------------L	--	N---I-------S------
	Anoxybacillus mongoliensis	WP_183244181	---------------------	--	A---I-------S------
	Anoxybacillus flavithermus	WP_003398487	---------------------	--	-------------------
	Anoxybacillus thermarum	WP_043964544	---------------------	S-	-------------------
	Anoxybacillus kestanbolensis	WP_077429780	---------------------	S-	-------------------
	Anoxybacillus ayderensis	WP_085789150	---------------------	SS	-------------------
	Anoxybacillus gonensis	WP_009362369	--------------------V	S-	---------------S---
	Anoxybacillus suryakundensis	CUA81142	---------------------	--	A---I-------S------
Other *Anoxybacillaceae* (0/ >15)	*Anoxybacillus amylolyticus*	WP_066328121	--SH-------------Q--M		HA--------R-EDVD---
	Anoxybacillus caldiproteolyticus	WP_199426772	--SY----------------M		EA-------VR-E-INI--
	Anoxybacillus tepidamans	WP_027410261	--SH----------------V		TA-------VR-EDVE---
	Anoxybacillus voinovskiensis	WP_183185897	--SH-------------Q---		HA--------R-EDID---
	Anoxybacillus rupiensis	WP_240371897	--SY----------------V		GA-------VR-EDIN---
	Geobacillus subterraneus	WP_033842622	--SY----------------L		AA-------SR-EDVS---
	Geobacillus vulcani	WP_031406037	--S------------------		-A-------SR-EDVA---
	Geobacillus kaustophilus	WP_044730361	--S------------------		-A-------SR-EDVA---
	Geobacillus zalihae	WP_081133139	--S------------------		-A-------SR-EDVA---
	Geobacillus thermodenitrificans	WP_029761552	--SY----I--------Q---		NA-------SR-ADVE---
	Geobacillus jurassicus	WP_066229468	--S----------I-------		---------SR-KDVA---
	Geobacillus stearothermophilus	1EPV_A	--S--------------E---		EA-------SR-AD-A---
	Parageobacillus toebii	WP_062678764	--SY---------I------V		TA-------CR-SDINI--
	Parageobacillus caldoxylosilyticus	KYD04496	--SY-------------E---		TA-------SR-SDVH---
	Parageobacillus thermoglucosidasius	WP_202614122	--SY--------------G--		-A-------SR-SDINI--
	Parageobacillus thermantarcticus	WP_090951467	--SY--------------N--		-A-------SR-SDINV--
	Saccharococcus thermophilus	NIK13518	--SY-----------------		TA-------SR-NDVH---
	Thermolongibacillus altinsuensis	WP_132949424	----V---------S------		---------VR-EDVV--S
Other Bacteria (1/ >50)	*Jeotgalibacillus campisalis*	WP_041056361	---H-----I----F---Q--		QA--V----SR-SD-E---
	Paenibacillus physcomitrellae	WP_229752571	--SY-------------D---		TA-------AR-ED-----
	Jeotgalibacillus proteolyticus	WP_104057877	---H----------F--RQ--		TA--V----SR--N-G---
	Peribacillus psychrosaccharolyticus	BAA31255	--GY-----------------		TA-------SR-ED-QI--
	Lentilactobacillus parakefiri	PAK87641	---GFC--I-----Q--RA--		TL-V----I-D-E------
	Bacillus methanolicus	WP_150915668	--SY-----------------		ND--------RAE-INI-G
	Lentilactobacillus parakefiri	WP_174659022	---GFC--I-----Q--RA--		TL-V----I-D-E------
	Robertmurraya korlensis	WP_251523310	---Y---------I-------		IA-------SR-EDI-V-V
	Bacillus kexueae	WP_243388347	--------------------V		T-----M--VR-SDVTI--
	Bacillus tepidiphilus	WP_225434225	--DY---------I---E---		EA--------R-ED-T---
	Robertmurraya siralis	WP_137744398	--SY-----------------		ND--------RAE-INI-G
	Neobacillus novalis	WP_066095270	--NY-----M---I---N---		NA--------R-ED--V--
	Peribacillus butanolivorans	WP_236695029	--DF-S---------------		SA-------SR-ES-G---
	Cytobacillus gottheilii	WP_257391512	--EY----A----F----S--		TA----M--AR-E------
	Mesobacillus zeae	WP_119113332	--SM-----------------		QA------PSR-E--AI--
	Neobacillus sedimentimangrovi	MCD4840301	---Y---------I----N--		NA-------SR--DCQI--
	Robertmurraya massiliosenegalensis	WP_026073566	--SY-----------------		-T--------RAE-INI-G
	Salimicrobium jeotgali	WP_102336383	--E-----L-----R---A--		T-----M-Y-R-ED-----
	Salimicrobium humidisoli	WP_095821840	--E-----L-----R---A--		T-----M-Y-R-ED-----
	Bacillus oleivorans	WP_097160031	--SMV---I----I-------		KA-------SR-ED-----
	Peribacillus butanolivorans	WP_252433436	--DF-S---------------		SA-------SR-ES-G---
	Peribacillus acanthi	WP_108671736	--DY---------IS------		EA--------R-SDSSV--
	Peribacillus deserti	WP_101645356	--DY----------H--N--L		-C-------SR-ED-S---
	Bacillus dafuensis	WP_057775731	--SY----I-----S------		TA-------CR-EH-GI--
	Peribacillus saganii	WP_117327550	--EM----I------------		EA-------IR-ED-----
	Neobacillus cucumis	MBI0580912	---H-----M-----------		TA-------SRAEDVQM--
	Bacillus yapensis	WP_126410048	--SS-------------Q---		-A--------RADHVNV--
	Bacillus rubiinfantis	WP_042357643	--VY-----M---I-------		TA-------SR-ED-GT--
	Robertmurraya kyonggiensis	WP_136833863	--SS-------------Q---		-A--------RADHVNV--
	Peribacillus muralis	WP_064462225	--DF-S--------S------		TA-------SR-ES-A---
	Weizmannia ginsengihumi	WP_025731228	---M-----------------		EA------TSR-ED-AI--
	Bacillus sinesaloumensis	WP_077620342	---Y-----I---IS--RG--		NE-------SR-TDVKI--
	Domibacillus aminovorans	WP_018395449	--KG----------S---A--		TA-------SR-NT-GI--
	Neobacillus niacini	WP_251639517	---Y-----M---I---N--V		LA--------R-ED----V
	Bacillus cihuensis	WP_028390573	--DY-------------R---		LA--------R-ES-KI-S
	Peribacillus huizhouensis	WP_182504038	--DY-------------R---		LA--------R-ES-KI-S
	Neobacillus thermocopriae	QAV25344	---------------------	S-	-------------------

Figure S4: Partial sequence alignment of the alanine racemase protein showing a 2 aa insert (highlighted) in a conserved region that is generally exclusively shared by species from *Anoxybacillus sensu stricto*. This CSI is also not shared by most other species from the family *Anoxybacillaceae* or other bacteria. One exception in a distantly related species is present.

Group	Species	Accession	263		306
Anoxybacillus sensu stricto (9/ 9)	*Anoxybacillus pushchinoensis*	WP_091700721	VHFAMDYLTMTTKWLLTGEK		DETFIDAKHKHVIVIGGGDTGADC
	Anoxybacillus flavithermus	WP_192952994	--------------------		------------------------
	Anoxybacillus mongoliensis	WP_183242234	--L------V----------		------------------------
	Anoxybacillus suryakundensis	WP_055441606	I-L------V----------		------------------------
	Anoxybacillus thermarum	WP_043966862	---------Q----------		--A---------------------
	Anoxybacillus tengchongensis	WP_183248210	I-L------V--------K-		E-----------------------
	Anoxybacillus gonensis	WP_009361416	---------R----------		E-------N---------------
	Anoxybacillus ayderensis	MCL6617554	---------R----------		E-------N---------------
	Anoxybacillus kestanbolensis	WP_077428509	--------------------		--------Y---------------
Other *Anoxybacillaceae* (0/ 8)	*Anoxybacillus voinovskiensis*	WP_183182892	--L------SV--S--DSHF	M	--N-----D---------------
	Anoxybacillus tepidamans	WP_183255284	I--------VV--S--DSNF	K	-GN-----D---------------
	Anoxybacillus rupiensis	WP_240371654	I--------L---S--DSNF	T	-DGL---RD---------------
	Anoxybacillus caldiproteolytic	WP_181556790	---------GV--S--DSNF	A	-GN-----D---------------
	Geobacillus stearothermophilus	KZM57387	---------GV--S--DSNF	A	-GQ-----D---------------
	Geobacillus thermoleovorans	WP_014195609	---------GV--S--DSNF	A	-GQ-----D---------------
	Parageobacillus thermoglucosidasius	WP_003251364	---------GV--S--DSNF	A	-GN-----D---------------
	Parageobacillus toebii	WP_062679379	---------GV--S--DSNF	A	-GG-----D---------------
Other Bacteria (4/ >30)	*Aeribacillus composti*	WP_144596811	---------E---S--NSNF	A	-KK-----D---------------
	Aeribacillus pallidus	WP_063388146	---------E---S--NSNF	A	-KK-----D---------------
	Bacillus pumilus	WP_111912763	--L------LA--SM-DSGF	K	-KN-----G-D-------------
	Bacillus altitudinis	WP_217967074	I-L------LA--SM-DSGF	K	-KN-----G-D-------------
	Bacillus safensis	WP_251219279	I-L------LA--SM-DSGF	K	-KN-----G-D-------------
	Bacillus xiamenensis	WP_008357296	I-L------LA--SM-DSGF	K	-KN-----G-D-------------
	Bacillus zhangzhouensis	WP_264009909	I-L------LA--SM-DSGF	K	-KN-----G-D-------------
	Bacillus australimaris	WP_060697018	I-L------LA--SM-DSGF	K	-KN-----G-D-------------
	Sediminibacillus albus	WP_093210479	---------AS--N--DAPL	K	--E-----G-D-------------
	Sediminibacillus massiliensis	WP_077621835	---------VS--S--DSGF	Q	-GNY----G-D-------------
	Bacillus atrophaeus	WP_094232153	--L------LA--SY-DSNF	K	-KR-----G-D-------------
	Priestia megaterium	TYR80808	I--------T---S--DSNF	K	-GQ-----G-D-------------
	Psychrobacillus psychrodurans	SFM51770	I-L------T---S--DSNF	K	-DQ--NT-G-D-------------
	Peribacillus deserti	WP_101641867	---------T---S--DS-S	K	-GQ---VQG-D-------------
	Bacillus alveayuensis	WP_044895352	---------AA--S--DSNF	E	-GN----ND---------------
	Paenisporosarcina quisquiliarum	SEM06362	I-L------T---S--DSNF	K	-DQ--NT-G-D-------------
	Psychrobacillus lasiicapitis	WP_142537125	--L------S---S--DSNF	K	-GL---T-G-D-------------
	Robertmurraya korlensis	WP_251518779	I-L------LS--S--DSNF	E	--N---VEG-D-------------
	Peribacillus saganii	WP_117325288	---------T---S--DSDF	Q	-GQ---T-G-D-------------
	Bacillus tequilensis	WP_024715464	--Y------LA--SY-DSNF	K	-KK-----G-D-------------
	Bacillus subtilis	WP_119996711	--Y------LA--SY-DSNF	K	-KK-----G-D-------------
	Bacillus licheniformis	WP_075747526	--Y------LA--SY-DSNF	K	-KK-----G-D-------------
	Bacillus cabrialesii	WP_213401433	--Y------LA--SY-DSNF	K	-KK-----G-D-------------
	Bacillus amyloliquefaciens	WP_065521167	--Y------LA--SY-DSNF	K	-KQ-----G-D-------------
	Bacillus velezensis	WP_083058766	--Y------LA--SY-DSNF	K	-KQ-----G-D-------------
	Bacillus nakamurai	WP_254490882	--Y------LA--SY-DSNF	K	-KQ-----G-D-------------
	Bacillus stercoris	WP_227098629	--Y------LA--SY-DSNF	K	-KQ-----G-D-------------
	Escherichia coli	MXF68432	I-L------LA--SM-DSGF	K	-KN-----G-D-------------
	Fictibacillus nanhaiensis	MBN3555450	-----N---QA--R--NI-V		S-P----EG---------------
	Fictibacillus phosphorivorans	WP_153238397	-----N---QA--R--NI-V		S-PL---EG---------------
	Trichococcus collinsii	WP_086987524	--M---F--AN-QNI-A--P		Q-SK-S--D------------T--

Figure S5: Partial sequence alignment of the glutamate synthase subunit beta showing a 1 aa deletion (highlighted) in a conserved region that is generally exclusively shared by species from the *Anoxybacillus sensu stricto*. This CSI is also not shared by most other species from the family *Anoxybacillaceae* or other bacteria. A few exceptions in distantly related species are present.

Group	Species	Accession	117		156
Anoxybacillus sensu stricto (8/ 8)	*Anoxybacillus pushchinoensis*	WP_091702457	VILTAPGKDEDVTIVMGVNEHMLSKE		HIIISNASCTTNCL
	Anoxybacillus flavithermus	WP_003397847	--------------------------		--------------
	Anoxybacillus gonensis	WP_009361826	------------------------P-		--------------
	Anoxybacillus mongoliensis	WP_183242069	------------------------P-		-V------------
	Anoxybacillus suryakundensis	WP_055440070	------------------------P-		-VV-----------
	Anoxybacillus tengchongensis	WP_032100347	------------------------P-		-VV-----------
	Anoxybacillus thermarum	WP_043967981	---------------I--------P-		--------------
	Anoxybacillus ayderensis	WP_021093797	---------------I--------P-		--------------
Other *Anoxybacillaceae* (0/ >15)	*Anoxybacillus caldiproteolyticus*	WP_181554682	--------N--------------DV-	Q	-F------------
	Anoxybacillus tepidamans	WP_027410193	--------N--I--------E--DVD	Q	-F------------
	Anoxybacillus rupiensis	WP_212387794	--------N--I--------E--DIA	Q	-F------------
	Anoxybacillus vitaminiphilus	WP_111644527	-V------N--I--------E--DV-	N	-F------------
	Anoxybacillus amylolyticus	WP_066327588	--------N-----------E--DID	N	-F------------
	Anoxybacillus calidus	WP_181537532	--------N--I--------E--DID	H	-F------------
	Anoxybacillus voinovskiensis	WP_183185263	--------N-----------E--DID	N	-F------------
	Geobacillus zalihae	WP_060788424	--------N------V-------DID	R	-F------------
	Geobacillus jurassicus	WP_066226837	--------N------V-------DID	R	-F------------
	Geobacillus kaustophilus	WP_044736378	--------N------V-------DID	R	-F------------
	Geobacillus stearothermophilus	WP_033008535	--------N------V----Q--DID	R	-F------------
	Parageobacillus thermantarcticus	WP_090947611	--------N------V-------DID	R	-F------------
	Parageobacillus toebii	WP_062753168	--------N------V-----V-DID	Q	-F------------
	Parageobacillus caldoxylosilyticus	WP_017437239	--------N------V-----V-DI-	K	-Y------------
	Parageobacillus thermoglucosidasius	WP_013400248	--------N------V-------DID	K	-L------------
	Saccharococcus thermophilus	WP_166910677	--------N------V-----V-DID	Q	-F------------
	Thermolongibacillus altinsuensis	WP_132947551	--------N------I-----V-DID	Q	-F------------
Other Bacteria (3/ >30)	*Pseudoneobacillus rhizosphaerae*	WP_230497106	--I------------------VFDVK	E	-HV-----------
	Bacillus alveayuensis	WP_044894535	--------N-----------G--DV-	K	-F------------
	Bacillus renqingensis	WP_199419460	---------------I----D--DI-	E	-E------------
	Neobacillus paridis	MBL4951160	--------N-----------E--NV-	E	-DV-----------
	Bacillus tianshenii	WP_204416274	--------------------EA-DLN	Q	-YV-----------
	Bacillus velezensis	WP_063636774	--------N-----------EQFNSD	E	-V------------
	Sutcliffiella horikoshii	WP_088019099	-V------N-----------EV-NMN	E	-FV-----------
	Effusibacillus pohliae	WP_018131164	--I--------I---------LYDP-	K	-SV-----------
	Metabacillus halosaccharovorans	WP_251684901	-----------I--------QNYNAN	E	--------------
	Neobacillus cucumis	WP_205177954	--------------------D--DIT	Q	-E------------
	Neobacillus fumarioli	WP_066370817	--------N-----------E--NI-	Q	-D------------
	Bacillus atrophaeus	WP_106270775	--------N-----------DQFDA-	E	-V------------
	Metabacillus niabensis	WP_226526262	-----------I--------ENYHAN	E	--------------
	Bacillus massiliglaciei	WP_110927188	---S----E-----------DV-DIN	E	-F------------
	Bacillus subtilis	AOL98752	--------N-----------DQFDA-	R	-V------------
	Bacillus canaveralius	WP_101578342	--------N-----------YS-DV-	K	-F------------
	Bacillus yapensis	WP_126409628	--------N------------D-NIN	E	-DV-----------
	Sutcliffiella rhizosphaerae	WP_230499516	--------N-----------EV-DLN	D	-FV-----------
	Tumebacillus flagellatus	WP_038090052	--I--------I-------D-LYDE-	K	-DV-----------
	Robertmurraya massiliosenegalensis	WP_019154804	--------N------------D-NL-	K	-D------------
	Bacillus kexueae	WP_243386085	--------E-----------DIYNEN	E	-V------------
	Bacillus nakamurai	WP_061523064	--------N-----------EQYNP-	E	-V------------
	Sutcliffiella halmapala	WP_078379447	-------------------DEV-DLD	K	-YV-----------
	Bacillus amyloliquefaciens	WP_061581273	--------N-----------EQFNPD	E	-V------------
	Neobacillus kokaensis	WP_191269907	--------N-----------EV-NI-	E	-E------------
	Cytobacillus luteolus	WP_193535731	--------N--I---------E-DT-	V	-N------------
	Cytobacillus oceanisediminis	WP_110066252	--------N-----------SA-NIK	E	-DV-----------
	Roseburia faecis	MCB6949648	--------N-----------SD-NI-	K	-H------------
	Neobacillus vireti	WP_024028573	--------N----------DE--DIS	E	-D------------
	Neobacillus novalis	WP_066090724	--------N----------DE--DIS	E	-E------------
	Neobacillus endophyticus	WP_173058726	--------N-----------E--DI-	K	-D------------
	Clostridium putrefaciens	WP_115642266	-----------I--------ED-NIK	T	-T------------
	Bacillus halotolerans	WP_233403583	--------N-----------DQFDA-	R	-V------------
	Metabacillus flavus	WP_211559734	-V---------------------KE-		-Q------------
	Metabacillus mangrovi	WP_155111426	--------N--------------KS-		-Q------------
	Bacillus oleivorans	WP_097159015	--------N-----------N--HPD		-E------------

Figure S6: Partial sequence alignment of the glyceraldehyde-3-phosphate dehydrogenase protein showing a 1 aa deletion (highlighted) in a conserved region that is generally exclusively shared by species from the *Anoxybacillus sensu stricto*. Note that the homologue for one species within this clade is missing. This CSI is also not shared by most other species from the family *Anoxybacillaceae* or other bacteria. A few exceptions in distantly related species are present.

Group	Species	Accession	58		89
	Anoxybacillus pushchinoensis	WP_091704556	WYALAKGGAAFYPYLIEQLQAYG		HQTGYKRVG
Anoxybacillus sensu stricto (9/ 9)	*Anoxybacillus flavithermus*	WP_003398180	-----------------------		---------
	Anoxybacillus mongoliensis	WP_183243585	-------------Q---------		-E-------
	Anoxybacillus suryakundensis	WP_055441372	-------------Q---------		---------
	Anoxybacillus tengchongensis	WP_183248833	-------------Q---------		---------
	Anoxybacillus ayderensis	WP_085789081	-------------H------T--		-E-------
	Anoxybacillus thermarum	WP_043965332	-------------H------T--		---------
	Anoxybacillus gonensis	WP_009362696	-------------H------T--		---------
	Anoxybacillus kestanbolensis	OOE04323	-------------C------T--		YE---E---
Other *Anoxybacillaceae* (0/ >15)	*Anoxybacillus tepidamans*	WP_027409670	--R------K---S---E--S--	E	TE---E---
	Anoxybacillus calidus	WP_181536917	--R------R---T---E-KS--	E	TE---E---
	Anoxybacillus caldiproteolyticus	WP_181556760	--R------K---T---E-KS--	E	TE---A---
	Anoxybacillus vitaminiphilus	WP_111645737	--R------R---A---E-KS--	E	TE---E-I-
	Anoxybacillus rupiensis	WP_212387970	--R--R---K---S--QE--S--	E	TE---E---
	Geobacillus jurassicus	WP_066234090	--R------K---S---E-ES--	E	TE---A---
	Geobacillus thermoleovorans	UPT60955	--R------K---S---E-ES--	E	TE---A---
	Geobacillus proteiniphilus	WP_074044711	--R------K---S---E-ES--	E	TE---A---
	Geobacillus thermodenitrificans	WP_099232840	--R------K---S---E--S--	E	TE---A---
	Geobacillus stearothermophilus	WP_193443834	--R---N--K---S---E---L-	E	TD---A---
	Geobacillus icigianus	WP_033021276	--R---N------S---E--S--	E	TE---A---
	Geobacillus zalihae	WP_060787973	--R---N--K---S---E---L-	E	TD---A---
	Geobacillus kaustophilus	WP_042382363	--R---N--K---S---E---L-	E	TD---A---
	Parageobacillus toebii	WP_062755360	--R------R---A---E-KF--	E	TE---E---
	Parageobacillus thermoglucosidasius	WP_064550735	--R------K---A---E-KS--	E	TE---A---
	Parageobacillus galactosidasius	WP_089097499	--R------R---A---E-KF--	E	TE---E---
	Parageobacillus caldoxylosilyticus	BDG36325	--R------K---T---E-KS--	E	TE---E---
	Parageobacillus thermantarcticus	WP_090951035	--R------R---T---E-KT--	E	TE---E---
	Parageobacillus toebii	KYD24772	--R------R---A--KE-KF--	E	TE---E---
	Thermolongibacillus altinsuens	WP_132949710	--R------R---V---E-KS--	E	TE---E---
Other Bacteria (2/ >50)	*Bacillus methanolicus*	WP_003348301	--Q-V----RY--D--D--E-D-	D	KE-------
	Halobacillus litoralis	WP_224872968	--R-V----RY--E--Q--E-D-	E	-E----K--
	Metabacillus litoralis	WP_251529414	--Q-V----RY--T--D--E-D-	E	TE-------
	Weizmannia coagulans	WP_017552785	--R---N--K---S---E---L-	E	TD---A---
	Neobacillus bataviensis	WP_007085606	--Q---S--SY--E--A--E-D-	E	ID-------
	Bacillus alveayuensis	WP_044895374	--R-V----R---T---E-KS--	E	TD---E---
	Bacillus thermotolerans	WP_040036794	----------Y--S---E-KRD-	E	TE---E---
	Thalassobacillus pellis	WP_205093463	--R------RY--S-----EED-	E	TD---R---
	Rossellomorea vietnamensis	WP_060671534	--R------KY--SI----E-D-	E	TE-------
	Metabacillus iocasae	WP_205187695	--Q-V----RY--A-------D-	E	QE-------
	Halobacillus trueperi	WP_258870972	--R-V----RY--E--R--E-E-	E	TR---R---
	Alteribacillus persepolensis	WP_091273672	--EM--R------E--S---ED-	E	QNV---K--
	Terribacillus aidingensis	WP_245864812	--R------SY--Q---E-RQD-	E	EE---A---
	Neobacillus piezotolerans	WP_115451047	----------Y--T--R--EED-	E	KE---S---
	Bacillus haikouensis	WP_172248896	--R------KY--Q--KE-ETL-	E	IE---R---
	Metabacillus sediminilitoris	WP_136355988	--Q-V----RY--E-V---K-D-	E	TD-------
	Mesobacillus subterraneus	WP_251616954	--F---N------S--D--EED-	E	KE---Q---
	Salirhabdus euzebyi	WP_174497267	--E-V----KY--T--K--E-D-	E	MD----Q--
	Halobacillus salinus	WP_135328284	--R-V----K---E--AE-EEA-	E	AD-------
	Peribacillus alkalitolerans	WP_163099908	--Q------KY--T--KE-ESV-	E	SD---DQ--
	Oceanobacillus neutriphilus	WP_188732834	---V-----LY--E-----KKD-	E	TD----K--
	Bacillus kexueae	WP_243388052	--R-V----R---T-----KED-	E	EE-------
	Halobacillus dabanensis	WP_075038012	--R-V----RY--E--R--EEA-	E	-E---R---
	Rossellomorea marisflavi	WP_053428851	--K-------Y--E---M-KED-	E	-D-------
	Edaphobacillus lindanitolerans	WP_076758342	-------------Q--AS-EQD-	E	TE---R---
	Fictibacillus nanhaiensis	WP_222463800	--Q------RY--E--A--E-D-	E	TD-------
	Bhargavaea beijingensis	WP_092098112	-------------E--RS-EQD-	E	TE---R---
	Halobacillus kuroshimensis	WP_206936206	--R-V----RY--E--Q--E-D-	E	-E----K--
	Bhargavaea massiliensis	WP_213423102	-------------E--RS-ELD-	E	TE---R---
	Fictibacillus barbaricus	WP_188404050	--Q------SY--E--R--E-D-	E	TE-------
	Neobacillus cucumis	WP_205181186	--Q------RY--E--A--E-D-	E	TD-------
	Sporosarcina luteola	MBB4823830	--S---A-----HE--PE-E-A-		ME---RK--
	Neobacillus thermocopriae	QAV26629	-------------C------T--		YE---E---

Figure S7: Partial sequence alignment of the FAD-dependent oxidoreductase protein showing a 1 aa deletion (highlighted) in a conserved region that is generally exclusively shared by species from the *Anoxybacillus sensu stricto*. This CSI is also not shared by most other species from the family *Anoxybacillaceae* or other bacteria. A few exceptions in distantly related species are present.

Group	Species	Accession	78		109
Anoxybacillus sensu stricto (9/ 9)	*Anoxybacillus pushchinoensis*	WP_091705020	RGRGIGKQMMEAIEQYAK		QHVTKVKLNAQTHA
	Anoxybacillus mongoliensis	WP_183240317	-------HI---------		---S----------
	Anoxybacillus tengchongensis	WP_183249382	-------RI---------		---S----------
	Anoxybacillus suryakundensis	WP_055440377	-------RI---------		---S----------
	Anoxybacillus flavithermus	WP_003395319	------------------		--------------
	Anoxybacillus ayderensis	MCL6617481	-------LI--------S		--------------
	Anoxybacillus thermarum	WP_043964679	-------LI--------S		---K----------
	Anoxybacillus gonensis	WP_035065061	----L--RI--------T		K-------------
	Anoxybacillus kestanbolensis	WP_252506478	-R--L--RI--------T		K-------------
Other *Anoxybacillaceae* (0/ >15)	*Anoxybacillus caldiproteolyticus*	WP_181555829	-N--A-RLI--S---F--	E	NGIS-I--------
	Anoxybacillus tepidamans	WP_183256193	-NK-A-RLI--R---F--	E	HG---A--------
	Anoxybacillus vitaminiphilus	WP_111646398	-S--A-RLI--S---F--	E	NGIA-T--------
	Anoxybacillus calidus	WP_181538413	-N--A-RLI--S---F-R	E	NGIA-T--------
	Geobacillus icigianus	WP_033018798	----A-RMV------L-T	S	KGAK----------
	Geobacillus stearothermophilus	WP_160268948	----V-RMV---L--L--	T	KGAK----------
	Geobacillus kaustophilus	WP_042379698	----A-RMV------L--	T	KGAKTA--------
	Geobacillus jurassicus	WP_066232127	----A-RMV------L--	T	KGAKT---------
	Geobacillus thermodenitrificans	WP_029761545	--C-V-QMV-A----L--	T	KGAK----------
	Parageobacillus thermantarcticus	WP_090948218	-D--A---L--T--MF--	E	-GIR----------
	Parageobacillus thermoglucosidasius	WP_003252273	----A---L--T--KF--	E	-GIR----------
	Parageobacillus caldoxylosilyticus	WP_042411098	----A-RLI--T---F--	K	-G-P----------
	Parageobacillus toebii	WP_062753716	--S-A--LI------F--	K	-G-P----------
	Saccharococcus thermophilus	WP_166911361	----A--LI-----RF--	Q	-G-S-L--------
	Thermolongibacillus altinsuensis	WP_132947733	-Q-----LI--K---F-R	E	-GIQ-A--------
Other Bacteria (2/ >30)	*Bacillus alveayuensis*	WP_044748635	-N--A-RLI--S---F-R	E	NGIA-T--------
	Margalitia shackletonii	WP_055740754	--K-A--II------H-S	E	-GL-RL--------
	Heyndrickxia sporothermodurans	GIN83954	--K-A--LI-D--H-F-T	D	-NIL-L--------
	Heyndrickxia vini	WP_202776986	--K-A--IV-D------A	Q	-D-HVL--------
	Bacillus ectoiniformans	WP_204552571	--T-A-R-------S---	S	HSLPA-------Q-
	Caldibacillus lycopersici	WP_263071556	-KY----LI--Q-IA---	E	N-FS-L--------
	Bacillus tepidiphilus	WP_153124995	-KK-A--II-D---E--A	K	-DLK-L--------
	Bacillus subtilis	CUB57374	-KK----II-D-L-A---	E	NSLS----H-----
	Ureibacillus chungkukjangi	WP_107934992	--KK--LLI--E--K---	Q	LG---L-----SY-
	Bacillus cihuensis	WP_028393108	--N-A-QTI-N---D---	M	-DLS-L--D---Q-
	Salicibibacter cibarius	WP_200124028	--T----EI-Q-L-D-VA	S	-GAGS-L-------
	Bacillus kwashiorkori	WP_062351946	--N-A--LL--KMI---Q	E	VNFS-----S----
	Bacillus cereus	WP_076869245	-KK----IV-D-L-A---	E	NSLS----H-----
	Sutterella wadsworthensis	WP_165649655	----V-RVL---VLN---	K	EGLQT-R-------
	Bacillus thuringiensis	WP_060629973	-KK----IV-D-L-T---	E	HSLP-L--H-----
	Bacillus wiedmannii	WP_098887725	-KK----IV-D-L-A--N	E	HSLS-L--H-----
	Falsibacillus albus	WP_121679105	--N-A--LI-DK--E---	D	GS-EYL--------
	Bacillus anthracis	PFU91635	-KK----IV-D-L-A---	E	NSIS-L--H-----
	Stenotrophomonas maltophilia	WP_164088160	-KK----IV-D-L-A---	E	NSLS-L--H-----
	Salicibibacter cibi	WP_200085129	--T-V--EI-Q-L-D-VA	S	-GAES-I-------
	Bacillus smithii	WP_048622908	--S-V--AI-----E--R	S	-N-PAL-----V--
	Rossellomorea aquimaris	WP_224877575	--K-A-REI-K-------	T	-SISTL------Y-
	Peribacillus deserti	MBM7693029	-QK-A--AI-DG--E---	Q	-EIS-L--------
	Bacillus enclensis	WP_058298724	--K-A-REI-Q------A	G	RSIS-L------Y-
	Bacillus pakistanensis	MBM7586561	--T-A--RI-D---E---	Q	-AIPSL------Q-
	Bacillus xiapuensis	WP_100330613	--K-S-RLI-----E---	E	HG-PA---G--I--
	Mesobacillus maritimus	WP_251426175	--T-A--AI--K------	S	KG-GAL--------
	Rossellomorea arthrocnemi	WP_201715244	--K-A-REL-L---E---	Q	-PLSQL------Y-
	Bacillus haikouensis	WP_172254755	--K-A-REI-L-----SA	E	RAI--L------Y-
	Mesobacillus persicus	WP_090746840	--T-A--AI--K---H-Q	T	-G-SAL--------
	Bacillus marasmi	WP_147533264	--T-A--LI-DR-----H	D	-GISSL--------
	Rossellomorea vietnamensis	WP_261751362	--K-A-REL-L---E---	R	-PLSQL------Y-
	Evansella tamaricis	MBU9710250	-KK-V-ESL-AKM-DV--		---DVM--------
	Neobacillus thermocopriae	QAV26120	----L--RI--------T		K-------------

Figure S8: Partial sequence alignment of the GNAT family N-acetyltransferase protein showing a 1 aa deletion (highlighted) in a conserved region that is generally exclusively shared by species from *Anoxybacillus sensu stricto*. This CSI is also not shared by most other species from the family *Anoxybacillaceae* or other bacteria. A few exceptions in distantly related species are present.

Group	Species	Accession	272		312
Anoxybacillus sensu stricto (9/ 9)	*Anoxybacillus pushchinoensis*	WP_208601848	IPVYQTYGMTETASQVATLSPEA		RHKHGSAGKPLFHMNIRI
	Anoxybacillus tengchongensis	WP_183250083	-------------------P-D-		---------------S---
	Anoxybacillus flavithermus	WP_041637796	-------------------P-D-		---------------G---
	Anoxybacillus suryakundensis	WP_055440172	-------------------P-D-		---------------S---
	Anoxybacillus mongoliensis	WP_183241846	-------------------P-D-		---------------S---
	Anoxybacillus kestanbolensis	WP_252506745	-------------------P-D-		---------------G---
	Anoxybacillus ayderensis	MCL6616240	---------------------D-		----------------V--
	Anoxybacillus thermarum	WP_043966584	---------------------D-		----------------V--
	Anoxybacillus gonensis	WP_009361641	---------------------D-		---Q------------V--
Thermolongibacillus (1/ 1)	*Thermolongibacillus altinsuensis*	TCL46519	----------------V--P--Y		LS-R--------PVQ---
Other *Anoxybacillaceae* (0/ >8)	*Anoxybacillus vitaminiphilus*	WP_111644349	---------------IV--A--Y	S	FT-L--------PAQ---
	Anoxybacillus amylolyticus	WP_148660444	---------------IV--A--Y	S	LS-L--------PAQL--
	Anoxybacillus calidus	WP_181537664	---------------IV--A--Y	S	FT-L--------PAQ---
	Anoxybacillus tepidamans	WP_183256389	---------------M---A--Y	S	IT-L--------PVQL--
	Anoxybacillus caldiproteolyticus	WP_181554818	---------------IV--A--Y	S	IT-L--------PAQ---
	Anoxybacillus voinovskiensis	WP_183185651	---------------MV--A--Y	S	LS-L--------PAQL--
	Geobacillus icigianus	WP_033018310	---------------IV--A--Y	S	LT-L--------PAQL--
	Parageobacillus toebii	WP_062677556	---------------I---A--Y	S	LT-L--------PSQL--
	Parageobacillus galactosidasius	WP_081188758	---------------I---A--Y	S	LT-L--------PSQL--
Other Bacteria (1/ >80)	*Sporolactobacillus pectinivorans*	WP_239984803	--------L------MV--P--F	M	FS-L---------GE---
	Sporolactobacillus shoreae	WP_135349231	--------L------MV--P--F	M	FS-L---------GE---
	Peribacillus acanthi	WP_108672242	------------S--IV--P--Y	S	LA-L--------PCS-K-
	Bacillus mesophilus	WP_163179427	---F-----------IV--A--D	S	LN-L---------SQ-K-
	Metabacillus lacus	WP_154307585	--I---------S--I---P--D	C	FT-L--------PCS-S-
	Sporolactobacillus inulinus	WP_010025990	M-I------S-----AV--P--Y	M	-K-S--------PLQ---
	Domibacillus robiginosus	WP_156153447	------------S--IV--A--D	A	FR-I--------PCA---
	Bacillus pakistanensis	WP_205176181	---------------IV--P--Y	S	IS-L--------PCE-E-
	Bacillus smithii	WP_003352705	---F--------S--IV--P--Y	S	LS-L--------PAQ---
	Acinetobacter baumannii	MPU18009	------------S--IC---ADY	M	LT-V--------QCQL--
	Jeotgalibacillus soli	WP_041090901	---F-----------F------D	S	-R-I--------TSE-S-
	Sporolactobacillus terrae	WP_255304659	M-I------S-----A---P--Y	M	-E-A--------PLQLQ-
	Caldibacillus lycopersici	WP_263073538	---F-----------IV--T--D	S	LA-L--------FCQV--
	Klebsiella pneumoniae	MBL3250417	------------S--IC---ADY	M	LT-V--------QCQL--
	Sutcliffiella horikoshii	WP_251434221	------------C--IV----DF	A	LS-L--------PCQL--
	Niallia taxi	WP_144458476	----YS------S--IV--P--D	A	AR-I--------PAQLK-
	Cytobacillus horneckiae	WP_241198897	--I------------I------D	S	LQRV--------PSQLK-
	Peribacillus alkalitolerans	WP_240338395	------------S--IV-----Y	S	LS-L--------PCS-K-
	Bacillales bacterium	MBE3570226	---F--------S--IV--P--Y	S	LS-L--V-----PAQ---
	Bacillus coahuilensis	WP_059283348	---------------IV--A--D	S	MR-L--------PCEVK-
	Peribacillus simplex	WP_061465336	------------S--IV--A--D	S	MA-I--------PSQL--
	Bacillus firmis	URM34921	------------S--IV--P--Y	S	FS-L--------PSQLK-
	Sporolactobacillus laevolacticus	WP_023510064	M-I------S-----A---P--Y	M	LD-A--------PIQ---
	Metabacillus litoralis	WP_146950798	---------------I---A--Y	C	LE-L--------SCD-KV
	Metabacillus bambusae	WP_207979892	------------S---V--T--H	S	MK-L--------SCQ-K-
	Pseudoneobacillus rhizosphaerae	WP_230497226	V---------------V-----D	S	LA-L--------PVQLK-
	Sporolactobacillus kofuensis	WP_253052910	M-I------S-----SV--P--S	M	LE-A--------P-Q---
	Niallia circulans	WP_185765588	----YS------S--IV--P--D	A	AR-I--------PAQLK-
	Compostibacillus humi	WP_188392798	---F-S------S--I-----TD	A	LR-L--------PAQL--
	Bacillus marasmi	WP_147532707	---F-----------IV-----D	S	MR-L--------PAQ-K-
	Bacillus andreraoultii	WP_082195199	---F----L---S--IV--A--D	S	IR-L--------YSQ---
	Neobacillus cucumis	WP_251572985	---F-S------S--IV-----D	S	F--L--------PSQL--
	Bacillus weihaiensis	WP_199445811	---------------IV-----Y	C	LK-L--------QCEV--
	Sporolactobacillus putidus	GGL46098	---F----L------T---P--Y	M	FT-I--------PAE---
	Bacillus mesophilum	WP_151576172	------------S---------Y	S	LT-L--------PAQLK-
	Novibacillus thermophilus	WP_077721390	---F----L------I---P--Y	M	AD-S--------PSEL--
	Bacillus norwichensis	WP_191815938	---F--------S--I------D	S	LR-L--------PSQLK-
	Lederbergia citri	WP_233713343	---F-----------IV-----D	S	FA-L--------PAQ-K-
	Bacillus salacetis	WP_119546190	------------S--IV-----D	S	LE-L--------PCELK-
	Cytobacillus gottheilii	WP_214475529	------------S---------Y	S	LT-L--------PAQMK-
	Aquibacillus koreensis	WP_259868882	V-----------S--IV----SD	A	LR-L--------PAQLK-
	Siminovitchia terrae	WP_120118731	---F--------S--I------D	S	LR-L--------PSQLK-
	Bacillus carboniphilus	WP_226539081	------------C--I------S	S	MV-L--------PCQ-K-
	Sporolactobacillus vineae	WP_033403886	V-------L------CV--P--Y	M	TS-I--------PLK---
	Bacillus xiapuensis	WP_100332092	---F-----------IV--P--Y	S	LT-L--------PSQ-K-
	Aquibacillus kalidii	WP_186576440	------------S--IV----RD	A	LD-L--------PAQLK-
	Neobacillus thermocopriae	QAV27607	-------------------P-D-		------------------

Figure S9: Partial sequence alignment of the o-succinylbenzoate-CoA ligase protein showing a 1 aa deletion (highlighted) in a conserved region that is generally exclusively shared by species from the *Anoxybacillus*-*Thermolongibacillus* clade. This CSI is also not shared by most other species from the family *Anoxybacillaceae* or other bacteria. One exception in a distantly related species is present.

			301		351
Anoxybacillus sensu stricto (9/ 9)	*Anoxybacillus pushchinoensis*	WP_091702529	QSLFYENFIARHYSFWKRYYSLLQQYAPH		FAHISLDDFYRAINESKPSLIR
	Anoxybacillus suryakundensis	WP_055441284	------------------H----------		--DV------------------
	Anoxybacillus mongoliensis	WP_183243401	------------------H----------		--DV------------------
	Anoxybacillus flavithermus	WP_012574974	-----------------------------		-VDV------------------
	Anoxybacillus tengchongensis	WP_183247900	-----------------------------		-VDV------------------
	Anoxybacillus ayderensis	WP_085789463	-----------------------------		-L-V------------------
	Anoxybacillus gonensis	WP_035066361	--------------------R--------		-S-V------------------
	Anoxybacillus thermarum	WP_043966361	--------------------R--------		-S-V------------------
	Anoxybacillus kestanbolensis	OOE04500	-----------------------------		-VDV------------------
Thermolongibacillus (1/ 1)	*Thermolongibacillus altinsuensis*	WP_132948306	---------G----------D-FK-----		--NV------------------
Other *Anoxybacillaceae* (0/ >15)	*Anoxybacillus voinovskiensis*	WP_183184280	---------G----Y---N-P-------E	Q	--NV-----------V------
	Anoxybacillus tepidamans	WP_183250844	---------G---CY---N-P-------E	Q	--NV---E-------A------
	Anoxybacillus caldiproteolyticus	WP_194520395	---------G----Y---N-P-------E	Q	--NVP--E-------A------
	Anoxybacillus vitaminiphilus	WP_111645712	---------G---AY---N-R--KE---E	Q	--NV---E-------A------
	Anoxybacillus calidus	WP_181536114	---------G---AY---N-R--KE---E	Q	-HTV---E-------A------
	Anoxybacillus amylolyticus	WP_066324548	---------G-------GI-P-F-----E	Q	--NV-----------V------
	Geobacillus stearothermophilus	WP_193443921	----F--MVG--------H--R-S----S	Q	--GV---A-------A------
	Geobacillus kaustophilus	WP_156133441	----F--M-G---A----H-PR------T	Q	--DV---A-------A------
	Geobacillus thermoleovorans	WP_156773249	----F--M-G---A----H-PR------T	Q	--DV---A-------A------
	Geobacillus thermocatenulatus	AST00926	----F--M-G---A----H-PR------T	Q	--DV---A-------A------
	Geobacillus zalihae	WP_157074351	----F--M-G---A----H-PR-L----T	Q	--DV---A-------A------
	Geobacillus vulcani	WP_031409830	----F--M-G---A----L-PR------A	Q	--GV---A-------A------
	Geobacillus thermodenitrificans	WP_029760865	----L--M-G--P-----H-PR------E	Q	--DV---A-------A------
	Geobacillus kaustophilus	KJE28647	----F--M-G---A----H-PR------T	Q	--DV---A-------A------
	Geobacillus jurassicus	WP_066229427	----F--M-G---A----H-PR------A	Q	--GVP--A-------A------
	Geobacillus icigianus	WP_033021384	----F--M-G---A----N-PR------A	Q	--GV---A-------A------
	Parageobacillus caldoxylosilyticus	WP_017437376	--------------Y---N-AR------E	Q	-SDV---A-------A------
	Parageobacillus toebii	WP_062677378	--------------Y---N-PR------E	Q	-SGV---A-------V------
	Parageobacillus thermoglucosidasius	UOE74955	-------------AY---N-PR------D	Q	-SGVP--A-------A------
	Parageobacillus thermantarcticus	WP_090948418	-------------AY---N-PR--R---D	Q	-SGVP--A-------A------
	Saccharococcus thermophilus	WP_166909357	-------------LY---N--R-R----E	Q	-SGV---A-------A------
Other Bacteria (1/ >30)	*Bacillus methanolicus*	WP_248891686	--------VG-------KN-D--KK--NG	Q	-DDVE-----------------
	Bacillus carboniphilus	WP_226538500	----F----G---G---KN-Q-FKE---K	A	-TDV----Y-K-----------
	Bacillus alveayuensis	WP_044748264	---------G---AY---N-R--KE---E	Q	-N-V---E--------------
	Bacillus tepidiphilus	WP_153122508	----F----G-N-----KH-E--KK---E	Q	-S-V------------------
	Sporosarcina globispora	WP_236694125	--------VG--F----KN-E--KE--SG	Q	-NDV-IE---------------
	Peribacillus simplex	WP_061144426	----F----G-N-----NK----KEF--G	Q	-DDVT------G----------
	Cytobacillus solani	WP_053476744	--------VG-------KN-D--KE-SAG	Q	-DEVG-----------------
	Cytobacillus praedii	WP_131236269	--------VG-------KN-D--KE-SEG	Q	-DQV------------------
	Peribacillus cavernae	WP_126864768	----F---VG-N-----KQ-D--K----G	Q	-D-VE-----------------
	Cytobacillus oceanisediminis	WP_110064168	--------VG-------KN-Q--KE--SG	Q	-DEV---G--------------
	Peribacillus butanolivorans	WP_190271278	----F----G-N-----NK-P--KE---D	Q	--DVK------G-------F--
	Cytobacillus firmus	WP_258743521	--------VG--F-----N-N--KE--SG	Q	-DDVP--A--------------
	Bacillus marinisedimentorum	WP_066174063	----W---VS-NH-----H-TE--KV-SP	Q	-D-V------------R-----
	Bacillus massiliigabonensis	WP_102274808	--------VG-N-----KN-A--KE-SEG	Q	-DEV------------------
	Priestia abyssalis	WP_078414677	----F-K--GKN------N-DV-K--T-G	Q	-DDV--------V---------
	Priestia koreensis	WP_242175001	----F-K--GKN------N-DV-K----G	Q	-DDVA------GV---------
	Cytobacillus eiseniae	WP_066395315	--------VG--P----KN-D--KE--SG	Q	-NDV--E---------------
	Bacillus firmis	URM34344	--------VG--R----KN-D--KE--SG	Q	-DDV-I-A--------------
	Mesobacillus jeotgali	WP_102263355	--------VG-N-----HN-----E-SDG	Q	-SGVEV------V---------
	Peribacillus asahii	WP_252290383	----F----G-NKH--EKQ---------E	Q	-TGV--EG--------------
	Bacillus salacetis	WP_119547154	--------VG-N-----KN----KE-GNG	Q	-DSVE--E--------------
	Sutcliffiella horikoshii	WP_226683528	--------VG--EN----H-ES--R--GG	K	-DD---EE--L-----------
	Rossellomorea vietnamensis	WP_148938560	--------VG-N----EKN----KE-GEG	Q	-DEVG-E---------------
	Mesobacillus subterraneus	WP_251486349	--------VG-NF----HN-------SAG	Q	-SEVEVEE--------------
	Mesobacillus selenatarsenatis	WP_041964547	--------VG-N-----HN-----E-SEG	Q	-SGVEVE-----V---------
	Sinobaca qinghaiensis	WP_120194046	----W---VG--PK--EKN-E---T---D	Q	-TGV--H--------A------
	Sutcliffiella cohnii	WP_066414286	--------VG-N------H-NK-NE--TG	A	-QNV------G---VA------
	Peribacillus saganii	WP_117326970	----F----G-NF---QKE-D--KK-TGD	Q	--NV-VE---------------
	Peribacillus acanthi	WP_108671173	----F----G-N-----KNFG--NK--KE	Q	-KDV--E-Y-------------
	Priestia filamentosa	WP_019392542	----F------N-H--QKN--A------E	Q	-KNVG-K----------L----
	Robertmurraya korlensis	WP_066049464	--------VG-NL----GNFN--KK---T	Q	-QDVTI-E--------------
	Peribacillus muralis	WP_064463657	----F----G-NH----NKF---KEF--D	Q	-NDVT------G-------F--
	Aeribacillus pallidus	RZI53101	-------L-G-N-H------NQ-KT---N	Q	-TGV--N--------V------
	Rossellomorea arthrocnemi	WP_201713271	--------VG-DH----KN----KE-STG	Q	-DSVD-----------------
	Peribacillus frigoritolerans	WP_251540732	----F----G-NH----NKF---KE---E	Q	-NDVT--E---G-------F--
	Aeribacillus composti	WP_199757332	-------L-G-N-H------NQ-KT---N	Q	-TGV--N--------V------
	Peribacillus castrilensis	MCD1159998	----F----G-NH----NKF---KE---E	Q	-NDVT--E---G-------F--
	Mesobacillus campisalis	KKK37704	--------VG-N-----KN-L---E-TDG	Q	---VE-E-Y-------------
	Cytobacillus kochii	WP_095372811	--------VS--QG--EHNFE--N----D	Q	-DR-T--E--------------
	Neobacillus thermocopriae	QAV26813	-----------------------------		-VD-------------------

Figure S10: Partial sequence alignment of the carboxypeptidase M32 protein showing a 1 aa deletion (highlighted) in a conserved region that is generally exclusively shared by species from the *Anoxybacillus-Thermolongibacillus* clade. This CSI is also not shared by most other species from the family *Anoxybacillaceae* or other bacteria. One exception in a distantly related species is present.

Group	Species	Accession	66		111
Anoxybacillus sensu stricto (9/ 9)	*Anoxybacillus pushchinoensis*	WP_091702745	HTKAVRRGAPNTFIVTDMPFMSY		STLNDALTHAKAIMQQSGANAVK
	Anoxybacillus flavithermus	WP_041638242	----------H------------		--V----Q---------------
	Anoxybacillus ayderensis	MCL6617471	-----------------------		--V----Q---------------
	Anoxybacillus gonensis	WP_035066985	-----------------------		--V----Q---------------
	Anoxybacillus thermarum	WP_043968230	-----------------------		--V----Q---------------
	Anoxybacillus suryakundensis	WP_055440627	-----------------------		---S---Q---------------
	Anoxybacillus tengchongensis	WP_183246667	-----------------------		---S---Q---------------
	Anoxybacillus mongoliensis	WP_183243132	-----------------------		---P---K---------------
	Anoxybacillus kestanbolensis	OOE04696	----------H------------		--V----Q---------------
Thermolongibacillus (1/ 1)	*Thermolongibacillus altinsuensis*	WP_132947112	-----------------------		YSREE--KN--------------
Other *Anoxybacillaceae* (0/ >15)	*Anoxybacillus caldiproteolyticus*	WP_181555925	-----------------------	H	LSME---RN-QR-----------
	Anoxybacillus tepidamans	WP_027408551	---------A-------------	H	LSKE---RN-QR-----------
	Anoxybacillus voinovskiensis	WP_183183359	-----------------------	H	LSKE---RN--R---EAN-----
	Anoxybacillus vitaminiphilus	WP_111643993	----------D--VI--------	H	ISREE--RN-QR---EA------
	Anoxybacillus amylolyticus	WP_066325756	-----------------------	H	LSKE---RN--RL--EANT----
	Anoxybacillus calidus	WP_181535816	-----------------------	H	ISKEE--RN-QR---ET------
	Geobacillus thermodenitrificans	WP_008879587	-----------------------	H	ASKEE--QN-RR-----------
	Geobacillus stearothermophilus	WP_095859559	-------------V---------	H	ASKEE--QN-RR-----------
	Geobacillus proteiniphilus	WP_074043318	-----------------------	H	ASKEE--QN-RR---E-------
	Geobacillus kaustophilus	WP_044731034	-----------------------	H	ASKEE--QN-RR---E-------
	Geobacillus jurassicus	WP_066229191	----A------------------	H	ASKEE--QN-RR-----------
	Geobacillus vulcani	WP_031408740	-----------------------	H	ASKEE--QN-RR---E-------
	Geobacillus thermoleovorans	WP_190304862	-----------------------	H	ASKEE--QN-RR---E-------
	Parageobacillus toebii	WP_062678516	-----------------------	H	LSMEE--RN-QR---E-------
	Parageobacillus thermoglucosidasius	WP_003249639	-----------------------	H	LSMEE--RN-QR-----------
	Parageobacillus thermantarcticus	WP_090948788	-----------------------	H	LSMEE--RN-QR-----------
	Parageobacillus caldoxylosilyticus	WP_026078514	-----------------------	H	LSKEE--RN-QR-----------
	Parageobacillus galactosidasius	WP_089097842	-----------------------	H	LSMEE--RN-QR---E-------
	Saccharococcus thermophilus	WP_208404504	-----------------------	H	LSREE--RN-QR-----------
Other Bacteria (3/ >30)	*Bacillus alveayuensis*	WP_044748082	-------------VI--------	H	ISKEE--RN-QR---ET------
	Listeria riparia	WP_036101215	--------------------L--	H	GSVEETVR--RE-I-ET----L-
	Listeria booriae	WP_185615595	----------D---------L--	H	GSMEETIR--RE-I-ET----L-
	Fictibacillus phosphorivorans	WP_066395610	-----K---------------T-	H	GSIDET-NN-RR-L-E---S---
	Fictibacillus norfolkensis	WP_191754084	-----K---------------T-	H	GSIDET-NN-RR-L-E---S---
	Fictibacillus halophilus	WP_198767610	-----K---------------T-	H	GSIDET-NN-RR-L-E---S---
	Listeria ivanovii	WP_014093243	-----K-------V-------T-	H	GSIDETIK--RQ-I-E---H---
	Bacillus carboniphilus	WP_226538524	-----K-----------------	H	ISKE-S-KN-T--K-----S---
	Fictibacillus nanhaiensis	WP_222461306	-----K---------------T-	H	GSIDET-KN-RR---E---S---
	Fictibacillus barbaricus	WP_188402071	-----K---------------T-	H	GSIDET-RN-RR-L-E---S---
	Listeria seeligeri	WP_185468323	-----K-------V-------T-	H	GSVDETIQ--RR-I-E---H---
	Alkalihalobacillus macyae	WP_048309265	-----K-----------L--LT-	H	-SF-ET-S--GRLL-EA--H---
	Bacillus coahuilensis	WP_010173148	-S---------------------	H	L-REKT-ST-AK-I-E-------
	Sutcliffiella rhizosphaerae	WP_230503342	-----K----S------------	H	LSMENT--N--K-V-E---H-L-
	Paenibacillus azotifigens	WP_240059762	-----K---KD--V---------	H	CS-ES--KN-A--V-E---D-L-
	Listeria weihenstephanensis	WP_036063198	-----K--------------L--	H	GSVEETIRN-RD-I-E-----L-
	Bacillus subtilis	WP_029726777	-----K-----------------	H	LSKE-T-KN-A--V-E---D-L-
	Bacillus halotolerans	WP_253295285	-----K-----------------	H	LSKE-T-KN-A--V-E---D-L-
	Bacillus rugosus	WP_166851407	-----K-----------------	H	LSKE-T-KN-T--V-E---D-L-
	Bacillus stercoris	WP_243572299	-----K-----------------	H	LSKE-T-KN-A--V-E---D-L-
	Bacillus mojavensis	WP_194382863	-----K-----------------	H	LSKE-T-KN-A--V-E---D-L-
	Bacillus cabrialesii	WP_215797420	-----K-----------------	H	LSKE-T-KN-A--V-E---D-L-
	Bacillus cereus	AUZ26855	-----K-----------------	H	LSKE-T-KN-A--V-E---D-L-
	Bacillus tequilensis	WP_174227224	-----K-----------------	H	LSKE-T-KN-A--V-E---D-L-
	Bacillus spizizenii	WP_019714756	-----K-----------------	H	LSKE-T-KN-A--V-E---D-L-
	Bacillus haikouensis	WP_172252476	-----K---------------T-	H	LSRE-T-KA-AR-V-E-------
	Bacillus vallismortis	WP_252685141	-----K-----------------	H	LSKE-T-KN-A--V-K---D-L-
	Sutcliffiella horikoshii	WP_088018532	-----K----S--V---------	H	LSMENT--N--R-V-E---H-L-
	Listeria rocourtiae	WP_185342678	--------------------L--	H	GSVEETIRN-RQ-I-ET----L-
	Priestia taiwanensis	WP_188386732	-S-------KE------------	H	LSKE-T-RN-RR---E-------
	Peptococcaceae bacterium	HHV64168	-------------V-V-L-YL--		A-PQ---RN-GCLI--G--D---
	Desulfitobacterium metallireducens	WP_006715565	----------E--V-V-------		P-VDI--SN-GRLI-EG--D---
	Neobacillus thermocopriae	QAV26981	----------H------------		--V----Q---------------

Figure S11: Partial sequence alignment of the 3-methyl-2-oxobutanoate hydroxymethyltransferase protein showing a 1 aa deletion (highlighted) in a conserved region that is generally exclusively shared by species from the *Anoxybacillus-Thermolongibacillus* clade. This CSI is also not shared by most other species from the family *Anoxybacillaceae* or other bacteria. A few exceptions in distantly related species are present.

			8		49
Anoxybacillus sensu stricto (9/ 9)	*Anoxybacillus pushchinoensis*	WP_091700886	NPTKLIFGRGQIEQLKKELAPYE		RILMVYGGGSIKKNGVYDD
	Anoxybacillus tengchongensis	WP_183249873	--------K----------TA--		H--I---------------
	Anoxybacillus suryakundensis	WP_055441426	--------K----------TA--		H--I--------------E
	Anoxybacillus mongoliensis	WP_183243987	-------------------PA--		H--I---------------
	Anoxybacillus ayderensis	WP_021093612	-------------H-----HS--		H--I---------------
	Anoxybacillus thermarum	WP_043965048	-------------H-----HS--		H--I---------------
	Anoxybacillus flavithermus	WP_012575910	--------K--------------		-------------------
	Anoxybacillus kestanbolensis	WP_252506928	-----------------------		-------------------
	Anoxybacillus gonensis	WP_035067397	-------------H-----HS--		---I---------------
Thermolongibacillus (1/ 1)	*Thermolongibacillus altinsuensis*	WP_132949001	--------K--L------IPL-G		K--L--------R--L--E
Other *Anoxybacillaceae* (0/ >10)	*Anoxybacillus vitaminiphilus*	WP_111645585	--------K-------Q--PK-G	Q	KV-L--------R--L--Q
	Anoxybacillus rupiensis	WP_183187208	--------K------RQ-IPKDA	K	N--I--------R--L--E
	Anoxybacillus tepidamans	WP_183253106	--------K-------Q-VPQ-G	K	-V-L--------R--L--E
	Anoxybacillus caldiproteolyticus	WP_181556388	--------K-------Q--PQ-G	K	KV-F--------R--L--E
	Geobacillus stearothermophilus	WP_049624800	--------K------RQ-VTR-G	K	KV-L--------R--L--E
	Geobacillus jurassicus	WP_066227907	--------K--L----E-VPR-G	K	KV-L--------R--L--E
	Parageobacillus thermantarcticus	WP_090951829	--------K-------E-IPR-G	K	KV-L--------R--L--E
	Parageobacillus caldoxylosilyticus	WP_017434835	--------K----------PR-G	K	KV-L--------R--L--E
	Parageobacillus thermoglucosidasius	WP_003248362	--------K-------E-IPR-G	K	K--L--------R--L--E
	Parageobacillus toebii	WP_062677753	--------K-------E-IPR-G	K	K--L--------R--L--E
	Parageobacillus galactosidasius	WP_089098056	--------K-------E-IPR-G	K	K--L--------R--L--E
	Saccharococcus thermophilus	WP_166908117	--------K----E-----PR-G	K	KV-L--------R--L--E
Other Bacteria (5/ >30)	*Paenibacillus lutrae*	WP_157332018	-----------L---P---SA-G	S	KV-L--------R-----Q
	Oceanobacillus polygoni	WP_149475227	--------K--L-K-AD-IR--G	K	-V-I-----------I---
	Bacillus massiliglaciei	WP_110928661	--------K-------E-IPA-G	K	---L-----------L--T
	Bacillus licheniformis	WP_176277397	--------K--L---R---KV-G	K	NV-L-----------L---
	Bacillus amyloliquefaciens	KJD52335	--------K--L---R---KV-G	K	NV-I-----------L--E
	Bacillus mycoides	ARJ23284	--------K--L----T-IPQFG	K	KV-L--------R--I--N
	Bacillus subtilis	WP_160832166	--------K--L---R--FKR-G	K	NV-L--------R--L--Q
	Bhargavaea beijingensis	WP_092097774	--------E----T-----SQ-G	K	KV-L--------R------
	Bacillus safensis	BBP91927	-----M--K--L-G--S---R-G	K	-V-L-----------L--N
	Bacillus atrophaeus	WP_239814465	--------K-----------Q-G	K	NV-L--------R--L--Q
	Paenibacillus chitinolyticus	WP_042226031	--------K--LQ--P---TA-G	K	-V-L--------RS-L--Q
	Bacillus paralicheniformis	WP_075754021	--------K--L---R---KV-G	K	NV-L-----------L--E
	Bacillus nakamurai	WP_254490771	--------K----------KQ-G	K	NV-L--------R--L--Q
	Bacillus haynesii	WP_182069200	--------K--L---R---KV-G	K	NV-L-----------L--E
	Bacillus cereus	TKJ00133	--------K--L----T-IPQFG	K	KV-L--------R--I--N
	Bacillus alveayuensis	WP_044747390	--------K-------E-IPK-G	K	KV-L--------R--L--E
	Bacillus vallismortis	WP_252683626	--------K----------KQ-G	K	NV----------R--L--Q
	Paenisporosarcina antarctica	WP_134209284	--V-----K--L--I-E--PT-G	K	KV-I-----------L--E
	Mesobacillus persicus	WP_090742923	--------K--L------VPK-G	K	K--L--------R--L--E
	Bacillus tequilensis	WP_024713007	--------K------R---KQ-G	K	NV-L--------R--L--Q
	Bacillus cabrialesii	WP_213401333	--------K------R---KQ-G	K	NV-L--------R--L--Q
	Pseudalkalibacillus berkeleyi	WP_236335291	--------K----A-E--IPQ-G	K	NV-V-----------L--Q
	Robertmurraya kyonggiensis	WP_136833848	--------K--L----T--PQ-G	K	KV-L--------R--L--E
	Bacillus velezensis	WP_101562384	--------K---D------KQ-G	K	NV-L--------R--L--Q
	Bacillus rugosus	WP_166848974	---R----K------R---KQ-G	K	NV-L--------R--L--Q
	Peribacillus cavernae	WP_126866752	--------K--V----A-IPQ-G	K	---L-----------L--Q
	Bacillus siamensis	PIK31549	--------K---D------KQ-G	K	NV-L--------R--L--Q
	Bhargavaea cecembensis	WP_008301155	--------E----A--N--PV-G	K	KV-L--------R------
	Lysinibacillus halotolerans	WP_122972204	--VR-H--K-------Q--PQ-G	H	NV-V-----------L--E
	Bacillus taeanensis	WP_113804052	--------K--L-S-QE--VH-G	E	N--V--------R--L--E
	Bacillus mojavensis	WP_010331655	--------K--L---R---KQ-G	K	NV-L--------R--L--Q
	Bacillus halotolerans	WP_101860036	--------K--L---R---KQ-G	K	NV-L--------R--L--Q
	Bacillus wiedmannii	WP_098208819	--------K--L----T-IPQFG	E	KV-L--------R--I--N
	Streptomyces harenosi	WP_164398151	--------K---D------KQ-G	K	NV-L--------R--L--Q
	Bacillus yapensis	WP_126405226	--------K--L----T--PQ-G	K	KV-L--------R--L--E
	Paenibacillus aquistagni	WP_169027067	-----M--T---AK--E-----Q		-V-L--------R--L--Q
	Pontibacillus halophilus	WP_026800957	-----Y--K--V---QQ-IPENA		NV-L--------R------
	Gottfriedia luciferensis	WP_069034476	-----W--KN-----AN-VQN-N		---I---------------
	Gottfriedia acidiceleris	WP_129689065	-----W--KN-----AN-VQE-N		---V---------------
	Arthrobacter citreus	QKE72967	-----W--KN-----AN-VRD-N		---V--------S------

Figure S12: Partial sequence alignment of the iron-containing alcohol dehydrogenase protein showing a 1 aa deletion (highlighted) in a conserved region that is generally exclusively shared by species from the *Anoxybacillus-Thermolongibacillus* clade. This CSI is also not shared by most other species from the family *Anoxybacillaceae* or other bacteria. A few exceptions in distantly related species are present.

Group	Species	Accession	176		213
Anoxybacillus_A (4/ 4)	*Anoxybacillus tepidamans*	MBB5324444	ILLSPTATWCVTFAEGE		IIVGSSDRFWTEMTREEEKKL
	Anoxybacillus voinovskiensis	WP_183184335	--V--------------		------E------GK-G----
	Anoxybacillus amylolyticus	WP_066327754	--V--------------		------E------GKGG----
	Anoxybacillus rupiensis	WP_221212020	----------I-L----		-M---AE---K-IAG-N----
Unnamed *Anoxybacillus* strains	*Anoxybacillus*	WP_159720278	----------I-L----		-M---AE---K-IAG-N----
	Anoxybacillus sp.	MCL6587605	--V--------------		------E------GK-G----
	Anoxybacillus sp. P3H1B	WP_066150113	----------I-L----		-M---AE---K-IAG-N----
	Anoxybacillus sp. UARK-01	WP_080862046	----------I-L----		-M---AE---K-IAG-N----
	Anoxybacillus sp. J5B_2022	WP_268851368	-----------------		-----A--------KDG----
	unclassified Anoxybacillus	WP_044745886	----------I-L----		-M---AE---K-IAG-N----
Other *Anoxybacillaceae* (0/ >15)	*Anoxybacillus caldiproteolyticus*	WP_181554759	------M---M------	KDN	--I--PE-----V-NRG--RM
	Anoxybacillus vitaminiphilus	WP_111644451	-----IM---I-----K	EDN	--I--HE-----L-NGK---I
	Anoxybacillus calidus	MBA2871794	-----VM---I-LV--K	EDN	--I--HE-----L-NSQ---I
	Anoxybacillus pushchinoensis	WP_091700271	--I---T---IA-T-EQ	SNN	-----A-------VGGI-R-I
	Anoxybacillus flavithermus	WP_241737738	--I---T----A-TQER	KNN	-----AE-----IVDNV-R-I
	Anoxybacillus suryakundensis	WP_055440134	--I---T---IA-T-ER	KNN	-----AE-----IFDDA-R-I
	Anoxybacillus mongoliensis	MBB5355115	--I---T---IA-T-ER	KNN	-----AE-----IVDDV-R-I
	Anoxybacillus ayderensis	MCL6616857	--I---T---IA-T-ER	KNN	-----AE------VDDV-R-I
	Anoxybacillus tengchongensis	MBB6177118	--I---T---IA-T-ER	KNN	-----AE-----IFDDV-R-I
	Anoxybacillus thermarum	WP_043966921	--I---T---IA-T-ER	KNN	-----AE------IDDV-R-I
	Anoxybacillus kestanbolensis	WP_237812526	--I---T----A-T-ER	KNN	-----AE-----IVDDV-R-I
	Geobacillus stearothermophilus	ALA69764	MM-T-S-L--IA-V--R	PDN	-VMA-TG---V-QAGTN-TR-
	Geobacillus kaustophilus	WP_011232302	MM-T-S-L--IA-V--R	PDN	-VMA-TG---V-QAGTN-TR-
	Geobacillus thermoleovorans	AMV12008	MM-T-STL--IA-V--R	PDN	-VMA-TG---V-QAGTN-TR-
	Geobacillus zalihae	WP_081133063	MM-T-S-L--IA-V--R	PDN	-VMA-TG---V-QAGTN-TR-
	Geobacillus proteiniphilus	OKO91838	MM-T-S-L--IA-V--R	PDN	-VMA-TG---V-QAGTN-TR-
	Parageobacillus thermoglucosidasius	WP_125009330	------T---I-----A	KDN	--IA------A-IING----M
	Parageobacillus galactosidasius	WP_081188742	------T---I------	KDN	--IA--------I-KR----K
	Parageobacillus toebii	WP_205424502	------T---I------	KDN	--IA--------I-KR----K
	Parageobacillus thermantarcticus	WP_090947680	------T---I-----A	KDN	--IA--------IINN----M
	Parageobacillus caldoxylosilyticus	WP_017434915	L-----T---I--V---	KDN	--IA----------KRG-RRR
	Saccharococcus thermophilus	NIK15792	L-----T---L--V---	ADN	--IA-A--------KKG---R
	Thermolongibacillus altinsuensis	WP_132947480	--I---T---L--V--R	KDN	-----A-------INDQ-R-M
Other Bacteria (0/ >50)	*Cytobacillus luteolus*	WP_193535889	--I---T---I-IL---	RES	VFL--KE---I-RNGDK---V
	Bacillus timonensis	MCA1032401	------T---I-MF--V	ENT	VYL--KE---I-K-N-S---V
	Bacillus alveayuensis	WP_044894462	-----IM---I-IV--K	EDH	--I--HE---I-V-NGK---I
	Cytobacillus suaedae	QOR65883	-MI---T---I-IL---	RES	VFL--KE---L-RNGDK-R-V
	Neobacillus thermocopriae	QAV27565	--I---T----A-T-ER	KNN	-----AE-----IVDDV-R-I
	Bacillus mesophilus	MBM7660480	-MI--IE---I-LV--K	EDC	VML--K----I-KHTN----V
	Priestia abyssalis	WP_078414078	--I---E---ISLL--K	EDS	VFQY-KE---N-LIGKTQR-I
	Jeotgalibacillus malaysiensis	WP_039810502	L-FT--GL--ISML-EG	EGT	VF--TTE---KVKQG-S-HT-
	Rossellomorea aquimaris	MCA1056846	-MIT---I--LS-L-F-	DGT	AYI--GE---LKKWGDT---V
	Jeotgalibacillus salarius	WP_134382999	L-FT--GL--MS-LDEG	DDT	VF---AE---KVKKG-S-HT-
	Litchfieldia salsa	WP_090849269	--IT-I--L-IS-L---	ENS	VFM--KE---I-KNGK----V
	Bacillus pinisoli	WP_272495711	-MI---E---I-ML--K	EDS	VFL--KEK----KHTN----I
	Mesobacillus foraminis	WP_214891838	--IT---A--I--L-D-	TDA	VYI--KEH--NKK-GDQ-V--
	Falsibacillus pallidus	WP_211318537	MVA----I--L--L--N	QDA	VYI--KEH--EKRDGKDSS-T
	Ureibacillus terrenus	WP_141602821	------ECL-I-LV-A-	EDA	FY--DGE----KKIGKRDV-I
	Jeotgalibacillus campisalis	WP_041060203	LVFT--EL--I-LL--A	DQS	AYI--M-----VKAG-Q--RM
	Metabacillus iocasae	WP_205188864	--IG--E---ISMI--R	SGS	VFQY-KE---N-RIG--K--V
	Fredinandcohnia onubensis	WP_099351942	-MI---T--LI-IL---	ENS	VFI-TN----I-RKGKHD--V
	Rossellomorea vietnamensis	WP_034765621	V-IT--GI--L--L-F-	EGT	AYI--GE---VKKWGDR-E-V
	Niallia taxi	WP_251663830	--VT---V--I-VL-A-	DSA	VF---NEK--VKKYNSK---I
	Calidifontibacillus oryziterra	WP_017756163	-----VT-Y-ISLL-S-	DMS	VYLTDRG-Y-F-GSG-N---R
	Falsibacillus albus	WP_121678578	--IT--TL--L-LL-HK	AGD	VFI--KEH--EKRSGKDSS-I
	Ureibacillus galli	WP_191707558	---T--ECL-I-LV-Q-	NQA	VF--N------KKVGKTS--M
	Metabacillus halosaccharovorans	WP_216774739	--IGT-EIY-I-IV-QQ	NDT	VFI--KEN--LARKG-DQ--F
	Bacillus haikouensis	WP_172253863	--IT--GV--LS-L-F-	DGT	AYI--G----VKKWGDT-TRV
	Metabacillus schmidteae	WP_175639936	--IGT-DIY-I-IV-QQ	NDT	VFI--KEK--LARKG-DH--I
	Lysinibacillus halotolerans	WP_122972946	---T--ECL-I-LV-Q-	EQA	VF--N------KKIGKTS--M
	Halalkalibacter akibai	WP_035662979	--I---EIY-I-LLD-S	EHS	VFEA--E---I-FVNQTR--R
	Heyndrickxia sporothermodurans	MBL5833408	L-IT--EV--II-L-E-	NEA	VYL--KE---IKRANRN-S-I
	Bacillus caldolyticus	WP_119877872	MM-T-S-L--IA-V--R	PDN	-VMA-TG---V-QAGTN-TR-
	Mesobacillus zeae	WP_119113930	---T---V--IKLL-D-	GDA	VFI--KEH--PLKRAR--T-I
	Rossellomorea arthrocnemi	WP_201715924	--IT--GI--L--L-F-	EGT	AYI--GE---LKKWGDM-A-V
	Priestia flexa	WP_210609312	--IT--E-I-IAMV--K	NGS	VFQY-KEN----LIGKDK--V
	Jeotgalibacillus aurantiacus	WP_264476752	L-FT--DL--ISIL-EA	NDA	VF---M----KVKHGDK-RS-
	Lysinibacillus endophyticus	WP_121212655	-I-T--ECI-I-VV-ND	NMA	VY--N-E-----KVGKVD--I
	Sporosarcina ureae	WP_029052832	---A--TCY-I-VL-D-	RMA	AYI--GE---SKRFG---V-V
	Solibacillus merdavium	WP_191705199	-I-T--ECL-I-LL-Q-	EQA	VFIAN-E----KKVGKN----
	Lysinibacillus composti	WP_124762568	-I-T--DCL-I-LV-Y-	NQA	VYL-NGE----KKVGKTD--V
	Metabacillus endolithicus	WP_247345170	--IGT-EIY-L-IV-QL	PET	VFI--KEK---ARRG-A-R--
	Heyndrickxia vini	WP_202779611	L-IT--EI--IV-L-E-	NEA	VFL--KE---SKRANGN-S-I
	Cytobacillus depressus	WP_151533625	-IVT--GV--IS-L-E-	DLA	VFI--KE---EKRGNGA---V
	Alkalihalobacillus nanhaiisediminis	WP_144449945	--I---EII---II--Q	EHS	VFEA--------YID-TR--R

Figure S13: Partial sequence alignment of the nuclease-related domain-containing protein showing a 3 aa deletion (highlighted) in a conserved region that is exclusively shared by species from the *Anoxybacillus_A* clade. This CSI is also not shared by most other species from the family *Anoxybacillaceae* or other bacteria.

Group	Species	Accession	112		160
Anoxybacillus_A (4/ 4)	*Anoxybacillus tepidamans*	WP_027408930	LFLFLLPHHQLKEIEAVLTHEYNHVCRLAKQPKKE		NTLLDAIVLEGLAE
	Anoxybacillus voinovskiensis	WP_183184705	---------ET---A-------S-----D--R-NI		Y----G-M------
	Anoxybacillus amylolyticus	WP_066323275	---------ET---A-------S-----N-----T		Y----G-I------
	Anoxybacillus rupiensis	WP_066148281	-------Q-SW------------------------		YP----VI------
Unnamed *Anoxybacillus* strains	*Anoxybacillus sp.*	MCL6585265	---------ET---A-------S-----D--R-NI		Y----G-M------
	Anoxybacillus sp. J5B_2022	WP_268852098	---------ET---A------------E----E-		Y----G-I------
	unclassified Anoxybacillus	WP_044745728	-------Q-SW------------------------		YP----VI------
Other *Anoxybacillaceae* (0/ >15)	*Anoxybacillus caldiproteolyticus*	WP_233414563	---------TE---K--F-------W-F--HE-QS	DD	Y------I------
	Anoxybacillus calidus	WP_181537908	---------TK--M--LI----------RRMV---	ED	Y----V--------
	Anoxybacillus vitaminiphilus	WP_111646010	---------TK--M--LII---------RRMA---	ED	Y-----------S-
	Anoxybacillus ayderensis	WP_085788322	--------VSNEH-Y-L-I---------HAD--D-	RD	Y----R--M-----
	Anoxybacillus gonensis	WP_009362189	--------VSNEH-Y-L-I---------HAD--D-	RD	Y----R--M-----
	Anoxybacillus flavithermus	WP_064214248	--------VSDEH-Y-L-I---------HVD--D-	RE	Y----R--M-----
	Anoxybacillus thermarum	WP_043964567	--------VSNEHVY-L-I---------HAD--E-	RE	Y----R--M-----
	Geobacillus stearothermophilus	WP_049624650	--F----D-GDE--A-LVA---------KQL-NEG	ED	A-----V-M-----
	Geobacillus subterraneus	WP_172418620	--F----D-RDE--A-LVV---------KRL-NDG	RD	A-----V-M-----
	Geobacillus jurassicus	WP_066231746	--F----D-RDE--A-LVA---------KQL-NEG	ED	A-----V-M-----
	Geobacillus vulcani	WP_031405101	--F----D-RDE--A-LVA---------KQL-NDG	QD	A-----V-M-----
	Geobacillus kaustophilus	WP_044732067	--F----D-CDE--A-LVV---------KQL-NEG	ED	A-----V-M-----
	Geobacillus thermoleovorans	WP_138188005	--F-F--D-GDE--A-LVA---------KQL-NEG	ED	A-----V-M-----
	Parageobacillus thermoglucosidasius	WP_064552469	--I------TH-----LI----------KQ-K-EN	ED	T------I------
	Parageobacillus galactosidasius	WP_089096954	-LI------TD-----LI----------K--K-ED	EG	V-------------
	Parageobacillus thermantarcticus	WP_090948273	--I------TD-----LI----------K--K-EN	ED	T-------------
	Parageobacillus toebii	WP_062679117	-LI------TD-----LI----------K--K-ED	EG	V-------------
	Parageobacillus caldoxylosilyticus	WP_042411074	---------TN-----LV----------N--K--Y	EK	M-----VI------
	Saccharococcus thermophilus	NIK16143	-I-------TD-----LM----------N--K--Y	EE	M-----V-------
	Thermolongibacillus altinsuensis	WP_132947786	----F----TKE--G-------------HYD--D-	KD	Y-II-S--------
Other Bacteria (0/ >50)	*Bacillus alveayuensis*	WP_044748686	---------TK------I------------LT---	ED	Y------I------
	Sporosarcina koreensis	WP_060207247	----IS--NTEI--G-L-----------NSF--R-	DE	Y----T-I------
	Bacillus timonensis	THE11244	----FS--NTQS--K-------------K-LQ-N-	AN	F----TVI---I--
	Metabacillus lacus	MRX73600	-----S-DCNK--VS--F----H-------DR-D-	EK	Y-I--TMI------
	Fredinandcohnia onubensis	WP_257215808	----FS--NSHD--K-------------K-YK-N-	AD	L-F--TVI---I--
	Oceanobacillus chungangensis	WP_115750769	I---IS-ENKQ---N-L-----------MRYD---	KE	YV---T-IM-----
	Mesobacillus subterraneus	WP_246017639	M----T-VEDE-----LFV---H----MQA-KRNP	EE	Y----S-I------
	Schinkia azotoformans	WP_035192544	-----TDQNTE---R---------------FR-S-	DQ	YV---T-I------
	Bacillus methanolicus	WP_237712819	M----T-LKKE--L-SIIV---H----MNR-K-DT	EN	Y----S--------
	Oceanobacillus zhaokaii	WP_114916178	I---IS-ENKQE--Q-LF----------MQYE-E-	KD	YV---T-I------
	Fredinandcohnia humi	WP_235812961	----FSS-NTDS--K-------------K-YK-N-	AE	F----TVI------
	Sporosarcina luteola	WP_147058129	----IS-QNTER-MK-I-----------S-YE-P-	DA	YV---S-I------
	Mesobacillus jeotgali	WP_257391791	M-I--T-LDDM-DL--LFV---H----MQA-K-SP	DE	Y----S-I------
	Cytobacillus gottheilii	WP_257391469	I----A-AQD---L--LFV---H----MNAIK--I	SE	Y----S--------
	Bacillus sinesaloumensis	WP_234987152	----VSK-NTQS--K-------------KYYK-A-	AN	Y-I--TVI------
	Cytobacillus praedii	WP_057763862	-----P-LEDK-----L-I---H-----NR-K-QI	LD	C----S--------
	Bacillus canaveralius	WP_101577748	V----S-GDDI-----LFV---H----MH-IK-PL	EK	Y----S-IM-----
	Neobacillus pocheonensis	MCM2533698	M----SQIDDT--L--L-V---H-----S-LN-RM	EE	Y----S-II-----
	Mesobacillus selenatarsenatis	WP_084135507	M----T-INDE--L--LFV---H----MQA-K-NP	AE	Y----S-I------
	Neobacillus dielmonensis	WP_042455275	M----SD-NDE-----L-V---H-----Q-ST--Y	EH	Y----S-II-----
	Robertmurraya massiliosenegalensis	WP_019153247	I----S-LEDI-----LFV---H----IN-HR--M	KE	Y----S-I------
	Calidifontibacillus erzurumens	WP_173732133	----IS-ENTDD--R-L-------------YN-N-	DN	YV---TVIM-----
	Oceanobacillus limi	WP_090871922	----ISGENTKN--R-LF----------T-NK---	KD	YV---SVI------
	Ornithinibacillus caprae	WP_155671189	-----SEDNSV---Q-LF----------SVYS-P-	ED	YV---T-I------
	Cytobacillus solani	WP_075209114	----IP-LEDE-----LIV---H-T---NR-K-HI	KD	F----S--------
	Terribacillus halophilus	WP_077306416	----VSAKNTEE--K-IF----------NNFE-R-	EE	Y----T--------
	Oceanobacillus massiliensis	WP_010651267	-----S-ENHDR--I-L----------ME-YK-E-	SD	Y-F--S-I---M--
	Bacillus dafuensis	WP_228460305	M---IS-FDDD--L--LFV---H-----HHKK-NS	EA	F----S-I------
	Virgibacillus oceani	WP_188456787	-----SANNTED--R-LF------I---SNYK-D-	KD	Y--I---I------
	Bacillus wudalianchiensis	OCA87480	----ISE-NTEE--K-L-----H-I---E-YK-D-	KD	F----T-IM-----
	Oceanobacillus caeni	WP_060667454	----ISKDNTE---R-LF----------SRYS---	ED	YV---T-I------
	Neobacillus niacini	WP_251631663	M----SSRITS--L--LFV---H-I---N--S-RF	EE	Y----S-II-----
	Caldibacillus thermoamylovorans	WP_041846528	-----SDQNSDN--R-L-V-----------NG-Q-	EN	YV---S-I------
	Radiobacillus deserti	WP_143896837	-----P-QVET-KLQ-------H-I-----LK-Q-	EE	FH---T-IA-----
	Virgibacillus halodenitrificans	WP_189779249	----VSENNTE--LQ-L-----------S-VDR--	KD	F----T-I------
	Aquibacillus halophilus	WP_153737075	-----SEDNSE--VK-MF----------S-FK-T-	VD	YV---T-I------
	Peribacillus saganii	WP_117327198	----IN-GIPE--L--LFV---H-----N-SK-R-	ED	F-----M-------
	Caldibacillus hisashii	WP_216407003	-----SDQNSDN--R-L-V-----------NG-Q-	EN	YV---S-I------
	Lentibacillus jeotgali	WP_010530324	----VSETNTE---R-L-------I---S-YS---	AE	Y----TVI------
	Neobacillus cucumis	MBI0579736	M----S-LDDS--L--LFV---H-----S-LN--M	NQ	Y----S-II-----
	Ornithinibacillus halophilus	WP_072889064	----IGD-LDKN--K-L-------I---HYYQ-NA	EE	F----T-I------
	Litchfieldia salsa	WP_090851194	-----SDTNSA--VK--F-------Y--S-NK-N-	RK	YH---T-I------
	Cytobacillus oceanisediminis	WP_110067581	-----T-DIDE--L--LFV---H-S--INR-K-NL	EE	Y----S-I------
	Radiobacillus kanasensis	WP_231094858	-----P-ELKT-KLQ---------I---QQLG-P-	ES	F----T-----I--
	Oceanobacillus arenosus	WP_115773531	V---VS-DNSGT--K-L-----------KHYD---	QD	YV---TVI------
	Neobacillus massiliamazoniensis	WP_245640467	M----SGVED-------FV--FH-----R-IN-NM	NE	Y----S-II-----
	Terribacillus saccharophilus	SEN49847	----IS-ENTED--K-IF----------NRFD-S-	EK	Y----T-I---I--
	Lentibacillus kapialis	WP_188633793	V---ISDMNTE---C-L-------I---S-YN---	SE	Y----TVI------

Figure S14: Partial sequence alignment of the DUF2268 domain-containing protein showing a 2 aa deletion (highlighted) in a conserved region that is exclusively shared by species from the *Anoxybacillus_A* clade. This CSI is also not shared by any other species from the family *Anoxybacillaceae* or other bacteria.

Group	Species	Accession	72		124
Anoxybacillus_A (3/ 4)	*Anoxybacillus tepidamans*	WP_183255419	YRVWISDRVHLASKFLIFGREVDVTVSFVPKVV		NGDLELGDPDLSLGELRIPV
	Anoxybacillus voinovskiensis	WP_183182738	----VN---YI--EIEL---NIPL-M--L-N--		---V--R-------A-H--A
	Anoxybacillus amylolyticus	WP_066325058	----VN---YI--EIEL---NIPL-M--L-N--		---I--L-------G-H--A
	Anoxybacillus rupiensis	WP_240371611	-----D---YV--QVPL-S-N--L-------I-	K	G-N---R--------WQL--
Unnamed *Anoxybacillus* strains	*Anoxybacillus sp. J5B_2022*	WP_268851326	---------YV--QIEV---N-PL----L-N--		------R-----------A
Other *Anoxybacillaceae* (0/ >15)	*Anoxybacillus vitaminiphilus*	WP_111645417	-D-QLT-----K--IPL------FL-T-E-Q--	D	----K-VH-EMM-----L--
	Anoxybacillus calidus	WP_181538105	-DIQLT-----K--IPL------FL-T-E----	D	--N-K-VN-EMT-----L--
	Anoxybacillus caldiproteolyticus	WP_194520226	----L----YV---LPV---D--L------N--	Q	G-N---R---M-----QL--
	Anoxybacillus flavithermus	WP_241735364	-N-QLA--LYIT-AIP---KP--L-MA-D---D	E	--HVV-AH-TMTF-Q--L--
	Geobacillus stearothermophilus	WP_095859376	----LA---YVS-EIP-L--S-ELV-------I	K	G--V--TE-TI---DWKL--
	Geobacillus proteiniphilus	WP_074043642	----LA---YVS-EIP-L--P-ELV--------	K	G-NV--TE-TI-F-DWKL--
	Geobacillus jurassicus	WP_066230730	----LA---YVS-EIP-L--P-ELV--------	K	G-NV--TE-TI---DWKL--
	Geobacillus thermoleovorans	UPT59463	----LA---YVS-EIP-L--S-ELV-------I	K	G-NV--TE-TI---DWKL--
	Geobacillus thermodenitrificans	AB067028	-D--LA---YVS-EIP----D-ELV---M----	K	G-NV--AE-TI--DDWKL--
	Geobacillus kaustophilus	WP_044737042	----LA---YVS-EIP-L--P-ELF--------	K	G-NV--TE-TI---DWKL--
	Geobacillus vulcani	WP_031409425	-D--LA---YVS-EIP-L--P-ELV--------	K	G-NV--TE-TI---DWKL--
	Geobacillus icigianus	WP_033020796	-H--LA---YVS-DIP-L--R-ELV--------	Q	G-NV--TE-VIL--DWKL--
	Geobacillus thermocatenulatus	WP_025950660	-D--LA---YVS-EIP-L--S-ELV--------	K	G-NV--TE-TI---DWKL--
	Parageobacillus thermoglucosidasius	WP_256835160	----LD---YV--ELPV---N-AL---------	K	G--I--QH-EIL-DDWKL--
	Parageobacillus galactosidasius	WP_081188690	----LD---YV---LPV---KM-LV---I----	K	G-NVV-ES-EI---DWEL--
	Parageobacillus caldoxylosilyticus	WP_244404804	----LD---YV---LPV-D--L-LV---I----	K	G-NV--QR-EI----WEL--
	Parageobacillus toebii	WP_062678648	----LD---YV---LPV----M-LV---I----	K	G-NVV-ES-EI---DWEL--
	Parageobacillus thermantarcticus	WP_090947771	----LD---YV---LPV---D-AL---------	K	G--V--QH-EIL--DWKL--
	Saccharococcus thermophilus	WP_166908886	----LN---YV---LSV---K--FV---I----	K	G-NV--QS-QI---DWQL-I
	Thermolongibacillus altinsuensis	WP_132948807	-EIQLA-Q-YIK-QIPV-EK-M-L--T-I-N-T	E	Q-NI--SN-TI---Q-KL--
Other Bacteria (2/ >10)	*Bacillus alveayuensis*	WP_052659353	-D-RLT---Q-K--IPL-R----LL-T-E---A	G	--N---VH-EMT-----L--
	Bacillus timonensis	MCA1030373	---ELTNQ-N-IGTIVA-NK-I-IVMA-E-V-D	K	Y-N-I-KQESI---Q--L--
	Listeria innocua	EHF3642776	-K-FVANN-NFTAEAK---EP-ELRLK-S----	D	--NI--SLT-M-V-A-PL--
	Listeria monocytogenes	EAG8744912	-K-FVANN-NFTAEAK---EP-ELRLK-L----	D	--NI--SLT-M-V-A-PL--
	Listeria marthii	MBF2478376	-K-FVA-N-NFTAEAK---EP-ELHLK-S----	D	--NV--TLK-M-A-A-PL--
	Neobacillus piezotolerans	WP_115452431	-Q-QVN-E-E-YGTVPF-S--LNMKLT---EAL	E	----L-KQKSI-V-Q--L--
	Listeria farberi	MBC1375520	-K-FVANN-NFTAEAK---EP-ELRLK-S----	D	--NV--TLK-M-V-A-PL--
	Bacillus renqingensis	WP_199417718	---LLA-E-E-YG-IPV-SE-INMKLT-E--AL	K	----V-RQKSM-----PL-I
	Neobacillus thermocopriae	WP_163251514	---LLG-D-E-YGSLPV-SQ--NMKLT----AL	E	----V-IQKSM-I-N-PL-I
	Mesobacillus subterraneus	WP_251480632	-Q-LLT-E-E-YGTLPL-SQ-LELRLT-E-QAL	K	----I-NQREI-V-K--L--
	Sutcliffiella halmapala	WP_078380299	-E-LLT---E-RGEMTV-E-DIPL-MT-I-E-Q	E	---VN-KQDSM-I-R-QV--
	Bacillus tianshenii	WP_204414427	-E-KLT---E-RGEMTV-E-DIPL-MT-I-Q-Q	E	---VN-KQDSM-I-R-QV--
	Aeribacillus pallidus	WP_094245662	-W-E-T-DIK-YGSL-A---DL-MVM--E-S-Q		Q-NVI-KVKS--V-R-SV--
	Aeribacillus composti NRS-1633	WP_063389682	-W-E-T-DIK-YGSL-A---DL-MVM--E-S-Q		Q-NVI-KVKS--V-R-SV--

Figure S15: Partial sequence alignment of the YpmS family protein showing a 1 aa deletion (highlighted) in a conserved region that is generally exclusively shared by most species from the *Anoxybacillus_A* clade, except for *A. rupiensis.* This CSI is also not shared by most other species from the family *Anoxybacillaceae* or other bacteria. A few exceptions in distantly related species are present.

Group	Species	Accession	67		109
Anoxybacillus_A (3/ 4)	*Anoxybacillus tepidamans*	MBB5324401	FLNITQEQIDQGISFTELIRWLKEYDRLRP		TVITWGSMDMKVL
	Anoxybacillus voinovskiensis	WP_183185908	--K---A---E---LA--VAR-QQ--A---		-------------
	Anoxybacillus amylolyticus	WP_066322895	--K---A---E---LA--VER--Q--A---		-------------
	Anoxybacillus rupiensis	WP_183187209	----A---V------E--VH--Q-----C-	N	------N------
Unnamed *Anoxybacillus* strains	*Anoxybacillus sp.*	MCL6585174	--K---A---E---LA--VAR-QQ--A---		-------------
	Anoxybacillus sp. J5B_2022	WP_268852380	--K---T---E---LM--VDR--Q--Q---		-------------
Other *Anoxybacillaceae* (0/ >10)	*Anoxybacillus calidus*	MBA2871517	--------V-K---LQ--VGR--K--S-Y-	S	------N------
	Anoxybacillus vitaminiphilus	WP_111645572	--------V-K----E--VG--R---S-Y-	S	------N------
	Anoxybacillus caldiproteolyticus	WP_181556398	--------V-E-------VAL-------C-	S	--V---N------
	Geobacillus stearothermophilus	ALA69858	----------R-M--Y--VSL-RQ---D--	T	--M----------
	Geobacillus thermocatenulatus	ASS98361	----------S-M--Y--VSL-RQ---E--	T	I-----G------
	Geobacillus thermodenitrificans	WP_029761678	----------S-M--Y--VSL-RQ---E--	T	--V---N------
	Geobacillus kaustophilus	WP_044736265	----------R-M--Y--VSL-RQ---E--	T	-------------
	Geobacillus subterraneus	WP_033843165	----------S----Y--VTL-RQ---E--	T	--V----------
	Geobacillus jurassicus	WP_066227931	----------R-M--Y--VSL-RQ---E--	T	-------------
	Parageobacillus thermoglucosidasius	OAT72556	---------N--M--H--VAL-RK---SL-	S	--V----------
	Parageobacillus caldoxylosilyticus	WP_017434826	--K------------Y--VTL-SK---A--	S	--V----------
	Parageobacillus thermantarcticus	WP_244151206	------------M--H--VAL-HK---SG-	I	--V----------
	Saccharococcus thermophilus	WP_166908098	--K------------Y--VTL-NK---T--	S	--V----------
Other Bacteria (1/ >30)	*Bacillus alveayuensis*	WP_044747381	--------V-K---LE--VG------S-Y-	S	------N------
	Peribacillus saganii	WP_117326233	--------VEK--T-Q--VF--S---K-QS	S	I-V---N------
	Peribacillus frigoritolerans	WP_260358701	------D-V------E--VNL--KI-EKH-	T	-IV---N------
	Priestia taiwanensis	WP_188389521	----E--DV------S--VTL-RQ--KQMN	S	--V---N------
	Ectobacillus panaciterrae	WP_028401389	--H----E-ES----S---AR-AK-EEV--	S	------N------
	Sporosarcina globispora	WP_053433873	--H-S---V----D-L--VKKM-DMN-N--	C	-IV---N------
	Peribacillus simplex	PCD07190	------D-V------E--VAL--KI-EQH-	T	-IV---N------
	Cytobacillus firmus	WP_061794251	--H-S---V----D-L--VKKM-DMN-N--	C	-IV---N------
	Bacillus alkalicellulosilyticus	WP_078552250	--HMS---V-S----E--VM--D--QS-G-	T	--V---NQ--R--
	Bacillus cereus	WP_249712307	--G-K--VV-K----S--VEK-A--EKRCK	P	--V---N------
	Bacillus albus	WP_166704700	--G-K--AV-K----L--VEK-A--EKRCK	P	-IV---N------
	Bacillus anthracis	KOS24031	--G-K--VV-K----S--VEK-A--EKRCK	P	-IV---N------
	Staphylococcus aureus	WP_113608253	--G-K--VV-K----P--VKK-S--ETKCK	P	-IV---N------
	Bacillus pacificus	WP_229130156	--G-K--VV-K----S--VEK-A--EKRCK	P	-IV---N------
	Bacillus paranthracis	WP_262734897	--G-K--VV-K----S--VEK-A--EKRCK	P	-IV---N------
	Bacillus thuringiensis	WP_060851618	--G-K--VV-K----S--VKK-A--ETKCK	P	-IV---N------
	Cytobacillus oceanisediminis	WP_071156702	--H-----V----D-L--VKKMIDMN-N--	C	-IV---N------
	Pseudoneobacillus rhizosphaerae	WP_230497932	------K-V-H--A-QQ-VQK-T--QQ-GQ	T	--V---N------
	Bacillus luti	WP_071712646	--G-K--VV-K----P--VEK-A--EKRCK	P	-IV---N------
	Bacillus nitratireducens	WP_212088355	--G-K--VV-K----H--VEK-A--EKRCK	P	-IV---N------
	Metabacillus crassostreae	WP_204955334	--Q----R--N--T-N---KV-EN-NYTQ-	V	------N------
	Bacillus toyonensis	WP_100063987	--G-K--VV-K----P---DK-A--EKRCK	P	-IV---N------
	Bacillus mobilis	WP_144564093	--G-K--VV-K----P--VEK-A--EKRCK	P	-IV---N------
	Rhodococcus qingshengii	TDL73955	--H-S-Q-V------Y----K-D-FNNQYE	T	-IV---N------
	Neobacillus niacini	WP_221823254	--H-S-Q-V------Y----K-G-FNNQYE	T	-IV---N------
	Bacillus wiedmannii	WP_262767413	--G-K--VV-K----P--VEK-A--E-RCK	P	-IV---N------
	Cytobacillus eiseniae	WP_066397252	--H-----V------L--VKK---LSDNQS	C	-I----N---R--
	Peribacillus butanolivorans	WP_053348742	------N-V-R----E--VSL--KI-EQA-	S	-IV---N------
	Metabacillus fastidiosus	WP_082799998	--H-S--KV-E-MD-H---DL--K--KGM-	N	------N------
	Metabacillus litoralis	WP_146948447	--Q-S--K--N--T-N---KV-EN-NYTQ-	V	------N------
	Peribacillus muralis	WP_064464312	------D-V-R----E--VSL-RKI-EQH-	T	-IV---N------
	Priestia abyssalis	WP_078414169	----Q-N-V-N---LE---HR-ED-E--AA	S	-I----N------
	Vibrio vulnificus	TDL87586	------D-V-R----E--VSL--KI-EQH-	T	-IV---N------
	Klebsiella pneumoniae	MBL2179632	--G-K--VV-K----P--VKK-A--ETKCK	P	-IV---N------
	Mesobacillus foraminis	WP_214897653	--------V-T----Q--VNR-GRMGQ-PA		SI----N------

Figure S16: Partial sequence alignment of the sporulation inhibitor KapD protein showing a 1 aa deletion (highlighted) in a conserved region that is generally exclusively shared by most species from the *Anoxybacillus_A* clade, except for *A. rupiensis.* This CSI is also not shared by most other species from the family *Anoxybacillaceae* or other bacteria. An exception is present in a distantly related species.

Group	Species	Accession	99		136
Anoxybacillus_B (2/ 2)	*Anoxybacillus vitaminiphilus*	WP_111644581	YPFIKITAEEHPRLIITRKV	N	KKDNGKYFGPYPNVQAA
	Anoxybacillus calidus	WP_181537500	--------------------	-	---K-------------
Other *Anoxybacillaceae* (0/ >15)	*Anoxybacillus caldiproteolyticus*	WP_194520690	--------------------		---H-R-----------
	Anoxybacillus tepidamans	WP_183253510	--------------------		Q--R-------------
	Anoxybacillus voinovskiensis	WP_183183542	---L----------------		Q--R-------------
	Anoxybacillus amylolyticus	ANB61900	---L----------------		Q--R-------------
	Anoxybacillus rupiensis	WP_212387812	--------------------		---R-------------
	Anoxybacillus flavithermus	KYD13978	---------K----V-----		---G-------------
	Geobacillus stearothermophilus	WP_095859750	---------Q----L-----		---G-------------
	Geobacillus thermocatenulatus	WP_025949416	---------Q----L-----		---G-------------
	Geobacillus kaustophilus	WP_044730646	---------K----L-----		---G-------------
	Geobacillus zalihae	WP_081212858	---------K----L-----		---G-------------
	Geobacillus thermodenitrificans	AB067949	---------K----L-----		---G-------------
	Geobacillus thermoleovorans	UPT60230	---------K----L-----		---G-------------
	Geobacillus jurassicus	WP_066232725	---------K----L-----		---G-------------
	Geobacillus vulcani	WP_031407858	---------K----L-----		---G-------------
	Geobacillus subterraneus	WP_168368866	---------T----L-----		---G-------------
	Geobacillus icigianus	WP_033020366	---------T----L-----		---G-------------
	Parageobacillus thermoglucosidasius	MBY6269291	---------K----V-----		---G-------------
	Parageobacillus thermantarcticus	WP_090947577	---------K----------		---G-------------
	Parageobacillus caldoxylosilyticus	WP_017435457	---------K----L-----		---G-------------
	Parageobacillus toebii	WP_062677860	---------K----------		---G--------H----
	Parageobacillus galactosidasius	WP_089097993	---------K----------		---G--------H----
	Thermolongibacillus altinsuensis	WP_132947582	---------T----V-----		---K-------------
	Saccharococcus thermophilus	WP_166910615	---------Q----------		---G--------H----
Other Bacteria (0/ >50)	*Bacillus alveayuensis*	WP_044894571	--------------------		---K-------------
	Mesobacillus maritimus	WP_221875280	-----L-S-R----------		-----------------
	Mesobacillus zeae	RID83214	-----L---R----------		---S-------------
	Priestia abyssalis	WP_078413968	-------G-K----------		-------------A---
	Bacillus sinesaloumensis	WP_077619008	---------K----L-----		---K-------------
	Mesobacillus harenae	WP_174729696	-----L---R--------R-		---S-------------
	Fredinandcohnia onubensis	WP_099352059	---------K----L-----		---K-------------
	Bacillus timonensis	WP_010678349	---------K----L-----		---K-------------
	Mesobacillus campisalis	WP_046523400	-----L---R----L-----		---S-------------
	Sutcliffiella horikoshii	WP_088019047	---------K----L-----		Q--K-------------
	Mesobacillus persicus	WP_090741469	-----L-S-R----------		---S-------------
	Mesobacillus subterraneus	WP_125479425	-----L---R--K-------		---S-------------
	Mesobacillus jeotgali	WP_079509966	-----L---R--K-------		---S-------------
	Bacillus taeanensis	WP_113804244	--Y-----------V-----		---K---------A---
	Mesobacillus selenatarsenatis	WP_167832759	-----L---R--K-------		---S-------------
	Bacillus tianshenii	WP_204416220	---------K----L-----		T--K-------------
	Mesobacillus boroniphilus	WP_213368967	-----L---R--K-------		---S-------------
	Metabacillus dongyingensis	WP_223437073	---------R-----V--N-		-----------------
	Metabacillus idriensis	WP_070876363	---------R-----V--N-		-----------------
	Peribacillus frigoritolerans	TDL82072	---------R-----V--N-		-----------------
	Sutcliffiella halmapala	WP_078379480	---------R----L-----		A--K-------------
	Cytobacillus firmus	WP_197248322	-----L---R-----T----		---R-------------
	Sutcliffiella cohnii	WP_066415446	---------R----------		---K-------A-----
	Jeotgalibacillus soli	WP_041090554	---L-----R----L-----		N--K---------A---
	Cytobacillus oceanisediminis	WP_144543517	-----L---R-----T----		---K-------------
	Bacillus firmis	URM33890	-----L---R-----T----		---R-------------
	Jeotgalibacillus terrae	WP_204729040	-----V---R----L-----		---K---------A---
	Jeotgalibacillus salarius	WP_134381603	-----V---R----L-----		---K---------A---
	Jeotgalibacillus malaysiensis	WP_039810203	-----V---R----L-----		---K---------A---
	Cytobacillus solani	WP_053477225	-----L---R-----T----		---K-------------
	Priestia megaterium	TYR82218	-------S-K----V----I		---K-------------
	Weizmannia ginsengihumi	WP_025727764	--Y--L-N-R----------		-----------------
	Cytobacillus eiseniae	WP_066396527	-----L---R-----T----		---K-------------
	Sporosarcina globispora	WP_053434182	-----L---R-----T----		---R-------------
	Bacillus massiliigabonensis	WP_102274339	-----L---R-----T----		---K-------------
	Niallia nealsonii	WP_101175748	-----L---K-----T----		---K-------------
	Caldibacillus lycopersici	WP_263073374	-----L---R----------		---K----------H--
	Priestia aryabhattai	OZT12173	-------S-K----V----I		---K-------------
	Fredinandcohnia humi	WP_057999496	---------K----L-----		---K-------------
	Bacillus marasmi	WP_147532560	-----L---R-----T----		---K-------------
	Priestia flexa	WP_210609396	-------S-K----V----I		---K-------------
	Jeotgalibacillus alimentarius	WP_041120967	-----V---R----L-----		-R-K---------A---
	Neobacillus notoginsengisoli	WP_118920796	-----L---R--K-------		-R-K-------------
	Neobacillus fumarioli	WP_066365917	-----L---R----------		---K----------G--
	Sutcliffiella rhizosphaerae	WP_230499480	---------K----L-----		A--K-------------

Figure S17: Partial sequence alignment of the excinuclease ABC subunit UvrC protein showing a 1 aa insert (highlighted) in a conserved region that is exclusively shared by species from the *Anoxybacillus_B* clade. This CSI is also not shared by any other species from the family *Anoxybacillaceae* or other bacteria.

Group	Species	Accession	178		223
Anoxybacillus_B (2/ 2)	*Anoxybacillus vitaminiphilus*	WP_111643591	KHSFWIPAVGGKMDTNV	E	ESKNQFWLEFDQEDAEKAGGIFYGKCAE
	Anoxybacillus calidus	WP_181536579	----------------I	-	D-----------K---------------
Other *Anoxybacillaceae* (0/ >25)	*Anoxybacillus voinovskiensis*	WP_183182953	----------------T		DN----------KATD------------
	Anoxybacillus amylolyticus	WP_066323492	----------------T		DN----------KATD------------
	Anoxybacillus caldiproteolyticus	WP_181555054	----------------T		-N----------KA-D------------
	Anoxybacillus tepidamans	WP_027408264	----------------T		DN----------KA-D------------
	Anoxybacillus pushchinoensis	WP_091703192	--------A-------T		DNT---F-T--EKR--E--RL-------
	Anoxybacillus suryakundensis	WP_055440207	-----V----------T		DNT---F-T--DKR--E--RL-------
	Anoxybacillus ayderensis	MCL6617620	----------------T		DNT---F-T--DKR--E--RL-------
	Anoxybacillus flavithermus	WP_004891270	----------------T		DNT---F-T--EKR--E--RL-------
	Anoxybacillus mongoliensis	WP_183241759	----------------T		DNT---F-T--DKR--E--RL-------
	Anoxybacillus gonensis	WP_009373477	----------------T		DNT---F-T--DKR--E--RL-------
	Anoxybacillus kestanbolensis	WP_077429522	--------A-------T		DNT---F-T--EKR--E--RL-------
	Anoxybacillus tengchongensis	WP_183247212	-----V----------T		DNT---F-T--DKR--E--RL-------
	Anoxybacillus thermarum	WP_043968524	----------------T		DNT---F-T--DKR--E--RL-------
	Geobacillus stearothermophilus	WP_063330100	----------------T		DN------V----ATD------------
	Geobacillus jurassicus	WP_066233365	----------------T		DN------V---KATD----V-------
	Geobacillus subterraneus	WP_168368342	----------------T		DN------V----ATD----V-------
	Geobacillus kaustophilus	WP_011230582	----------------T		DN------V---KATD----V-------
	Geobacillus thermocatenulatus	WP_089113955	----------------T		DN------V---KATD----V-------
	Geobacillus thermoleovorans	UPT58851	----------------T		DN------V---KATD----V-------
	Geobacillus icigianus	WP_033022144	----------------T		DN------V---KATD------------
	Parageobacillus thermoglucosidasius	WP_003252000	----------------T		DN----------K---------------
	Parageobacillus caldoxylosilyticus	WP_017434663	----------------T		-N----------KA-D----V-------
	Parageobacillus thermantarcticus	WP_090948933	------------I-A-N		DN----------K-----N---------
	Parageobacillus galactosidasius	WP_089097032	--------I-------I		-N----------KK-D---N--------
	Parageobacillus toebii	WP_062678290	-----V----------T		-N----------KA--------------
	Saccharococcus thermophilus	WP_166911055	--------A-------I		-N----------KA-D------------
	Thermolongibacillus altinsuensis	WP_132948015	----------------T		DN---------SKR--E-----------
Other Bacteria (0/ >50)	*Bacillus alveayuensis*	WP_044893893	----------------T		-N--------------------------
	Priestia abyssalis	WP_078409350	----------------T		DVV--------SKK--E-----------
	Mesobacillus jeotgali	WP_079508307	-------S--------T		DNV-K------SKG-DE---L-------
	Bacillus methanolicus	WP_248893370	----------------T		-NV-K------PKK-NE--NL-------
	Bacillus carboniphilus	WP_226538464	-------SA-------T		-NE---------QK--E--E--------
	Mesobacillus subterraneus	WP_226600427	-------SA-------T		DNI-K------SKG-DE---L-------
	Priestia koreensis	WP_053399780	-------S----I----		-ND-K---V--SKK--------------
	Mesobacillus persicus	WP_090747605	-------P--------T		DNV-K-------TK-DE-----------
	Neobacillus cucumis	WP_205179490	----------------T		DNV-K------NKR-NE--NL-------
	Priestia megaterium	WP_168242676	------------L---T		-NI-K------DKK-NE--NL-------
	Bacillus acidicola	WP_066264871	--------I--------		DNV-K------SKK-QE--NL-------
	Mesobacillus boroniphilus	WP_213366684	-------S--------T		DNI-K------SKG-DE---L-------
	Bacillus timonensis	MCA1029928	----------------T		-NT-K------SKATAE---L-------
	Heyndrickxia oleronia	WP_058002516	-----------------		DNI-K---K--SKK--E--NL-------
	Litchfieldia salsa	WP_090850534	-------S--------T		DNV-K-----S--GTD--N-L-------
	Peribacillus alkalitolerans	WP_163101370	----------------T		DGV-K------SQKS-EV-NL-------
	Robertmurraya kyonggiensis	WP_136829235	----------------T		DNI-K------SKG--A--EL-------
	Mesobacillus maritimus	WP_221873963	-------P--------T		DNV-K------PAK-DE-----------
	Neobacillus niacini	WP_034674188	-----V------L---T		-NV-K------SAK--E--DL-------
	Bacillus yapensis	WP_126408485	----------------T		DNI-K------SKG--A--EL-------
	Mesobacillus selenatarsenatis	WP_041965549	-------SA-------T		DNI-K------SKG-DE---L-------
	Bacillus sinesaloumensis	WP_077617692	----------------T		DNV-K-----NS-KT-E---L-------
	Bacillus tepidiphilus	WP_153124295	-----V----------T		DNV--------SKK-QE-NNL-------
	Bacillus canaveralius	WP_101576485	------------I---T		-NV-K------EKA-DE--NL-------
	Neobacillus thermocopriae	QAV26354	--------A-------T		DNT---F-T--EKR--E--RL-------
	Mesobacillus foraminis	WP_214894759	----------------T		-NV-K------GKASDE--SL-------
	Neobacillus bataviensis	WP_144562597	------------I---T		DNA-K------DKR-NE--NL-------
	Litchfieldia alkalitelluris	WP_078547368	-------S--------T		DNV-K-F------GTD----L-------
	Neobacillus endophyticus	WP_173065860	--A-----------V-T		DNV-K------EKK-DE--NL-------
	Neobacillus drentensis	WP_235743065	------------I---T		DNA-K------DKR-NE--NL-------
	Peribacillus glennii	WP_117322768	--------A--------		-GE--------SKK--NVKDV-------
	Bacillus testis	WP_050614654	-----L--A--------		DGI---Y----SKK-DE--N--------
	Weizmannia acidiproducens	WP_235801527	-------S----L-A-T		DNI-K----I--KK--E--NV-------
	Neobacillus pocheonensis	MCM2533530	--A-----------V-T		DNI-K------NTK-NE--NL-------
	Cytobacillus kochii	WP_095369654	--------A---L---T		-NT-K------SKK-DEV-N--------
	Bacillus mediterraneensis	WP_071459319	--------A-------T		DNI-K---K--SKS----ENV-------
	Neobacillus rhizosphaerae	WP_248733718	------------L---T		DNI-K------DKR-NE--NL-------
	Robertmurraya massiliosenegalensis	WP_019152926	-------SI-------T		DNV-K---N--S-G-----EL-------
	Pseudoneobacillus rhizosphaerae	WP_230496904	------------L---T		DNI-K---K--SDKS-E---L-------
	Neobacillus soli	WP_066066395	------------L---T		-NI-K------DKR-ND--NL-------
	Robertmurraya siralis	WP_095313168	-------S--------T		DNI-K---K--SKG----NEL-------

Figure S18: Partial sequence alignment of the cytochrome c oxidase subunit II protein showing a 1 aa insert (highlighted) in a conserved region that is exclusively shared by species from the *Anoxybacillus_B* clade. This CSI is also not shared by any other species from the family *Anoxybacillaceae* or other bacteria.

Group	Species	Accession	140		174
Anoxybacillus_B (2/ 2)	*Anoxybacillus vitaminiphilus*	WP_111644636	GKRHGVTKDMAVITP	S	RALVGKVQHVSPFTATIQL
	Anoxybacillus calidus	WP_181537441	-E------G------	-	K------------------
Other *Anoxybacillaceae* (0/ >15)	*Anoxybacillus pushchinoensis*	WP_091702175	-SQ---E-N------		QG-I---RS----S--V--
	Anoxybacillus flavithermus	WP_099668865	-SQ---K--------		QG-I---RS----S--V--
	Anoxybacillus suryakundensis	WP_055441820	-SQ---K--------		QG-I---RS----S--V--
	Anoxybacillus thermarum	WP_043965226	-SQ---K--------		QG-I---RS----S--V--
	Anoxybacillus salavatliensis	WP_257637212	-SQ---K--------		QG-I---RS----S--V--
	Anoxybacillus ayderensis	WP_085788067	-SQ---E--------		QG-I---RS----S--V--
	Anoxybacillus mongoliensis	WP_183241220	-SQ---K--------		QG-I---RS----S--V--
	Anoxybacillus gonensis	WP_009361734	-SQ---K--------		QG-I---RS----S--V--
	Anoxybacillus tengchongensis	WP_032100213	-SQ---K--------		QG-I---RS----S--V--
	Anoxybacillus kestanbolensis	WP_077428030	-SQ---E-N------		QG-I---RS----S--V--
	Geobacillus stearothermophilus	WP_049624756	-EQ---K--------		AG-------A-Q--S-V--
	Geobacillus kaustophilus	WP_044730701	-EQ---K--------		AG-------A-Q--S-V--
	Geobacillus zalihae	WP_081212832	-EQ---K--------		AG-------A-Q--S-V--
	Geobacillus jurassicus	WP_066232031	-AQ---K--------		AG-------A-Q--S-V--
	Parageobacillus thermoglucosidasius	WP_003248884	-EQ---K--------		EG-------A-Q--S-V--
	Parageobacillus thermantarcticus	WP_090947531	-EQ---K--------		EG-------A-Q--S-V--
	Parageobacillus caldoxylosilyticus	WP_244380214	-EQ---K--------		EG-------A-Q--S-V--
	Parageobacillus toebii	WP_143415124	-QQ---K--------		EG-------AAQ--S----
	Thermolongibacillus altinsuensis	WP_132947636	-EQ---K--------		-G-----RS----SSNV--
Other Bacteria (4/ 80)	*Anaerobacillus alkalilacustris*	WP_071311134	-EQ---KR------S		KG-----K---Q--S----
	Anaerobacillus alkalidiazotrophicus	WP_071389989	-EQ---KT------S		KG-----K---Q--S----
	Salsuginibacillus kocurii	WP_018921617	-SE--IER----V--		QG-I-------Q--S-V--
	Fredinandcohnia humi	WP_057998620	------KV------S		KG-I---K-ANE--S----
	Evansella cellulosilytica	WP_013489616	-TQ--IE-----M-S		QG-I---NQ--Q-------
	Mesobacillus foraminis	WP_214892069	--S---Q-N---M-S		-G-I---KSTT---S-V--
	Anaerobacillus alkaliphilus	WP_129079760	-EQD-IER------S		KG-----R---Q--S-V--
	Sutcliffiella deserti	WP_223702923	--QD--E-----V-A		GG-I---K-AN---S-V--
	Bacillus mesophilus	WP_163179221	-SN---EIN-----S		EG-I----Y-----S-V--
	Mesobacillus campisalis	WP_046526210	--S---E-N---M-S		-G-I---KSTT---S-V--
	Mesobacillus subterraneus	WP_251479334	--S---E-N-----A		-G-I---KSTT--SS-V--
	Bacillus timonensis	WP_136377632	-T----KV------S		KG-I---K-ANS--S----
	Anaerobacillus isosaccharinicus	WP_071316126	-EQD-IER------S		KG-----M---Q--S-V--
	Bacillus tianshenii	WP_224838369	--QD--V-------A		GG-I---K-AN---SSV--
	Fredinandcohnia onubensis	WP_099362057	-T----EV------S		KG-I---K-ANT--S----
	Anaerobacillus arseniciselenatis	WP_071314031	-EQ---EV------S		KG-----NN--Q--S-V--
	Aeribacillus pallidus	WP_066249582	-EQ--IK-------A		KG-I---KNT-E--S-V--
	Sutcliffiella halmapala	WP_078379529	--QD--E-------A		GG-I---K-AN---SSV--
	Mesobacillus persicus	WP_090741255	-SS---E-N---M-A		-G-I---KSTT---S----
	Bacillus pinisoli	WP_246940844	-SN--IEAN-----S		EG-I--I-D-----S-V--
	Evansella caseinilytica	WP_090889299	-RQ--IEEG-----S		KG-I--IKQ--Q----V--
	Alteribacter natronophilus	WP_138810923	-A---IEE------S		KG-I---RD--QL-S-V--
	Schinkia azotoformans	WP_003331807	-EQ---K-N-----S		EGM---IKQ--Q--S-V--
	Bacillus suaedaesalsae	WP_204205228	-SN---AEN-----S		EGFI---KF-----S----
	Alteribacter lacisalsi	WP_110516318	-A---IEE------S		KG-I---RD--QL-S-V--
	Mesobacillus maritimus	WP_221875336	-SS---E-N---M-A		-G-I---KSTT---S----
	Calidifontibacillus erzurumens	WP_173730821	-EQ---K-N-----S		EGMI--IKQ--Q--S-V--
	Mesobacillus jeotgali	WP_102263731	-AS---E-N-----A		KG-I---KSTT--S--V--
	Metabacillus lacus	WP_154306546	-SQ---EA------S		-GM--R-KS--S-VS-V--
	Mesobacillus harenae	WP_174729853	-QAN-IES------S		KG-I---KNTT-----V--
	Priestia abyssalis	WP_078413917	--V---EE------A		QG-I--IK-A-Q--S-V--
	Peribacillus muralis	WP_241577427	---N--E-N-----S		NG-----KSATD----V--
	Natronobacillus azotifigens	WP_268779167	--Q---E-------G		KGMI--I-ST-Q--S-VL-
	Bacillus mediterraneensis	WP_071460269	--SD--K-N---M-S		-G-I--IKSTT-----V--
	Bacillus alveayuensis	WP_044892906	-EL---E-------A		KG-I---KST-Q--S----
	Bacillus sinesaloumensis	WP_077618952	-T----KV------S		KG-I---KQANS--S----
	Peribacillus simplex	WP_061464252	---N--E-N-----S		KG-----KTATD---SV--
	Peribacillus frigoritolerans	WP_252267275	---N--E-N-----S		NG-----KSATD---SV--
	Brevibacillus migulae	WP_134686068	-LQ--IK-------S		-G-I-RIES--N-SS-VE-
	Bacillus sp. O3113	WP_141433625	-TT---E------SS	N	GS-I---KNSQ-Y-S-V--
	Bacillus manliponensis	WP_034642484	-AQ---E-----VAA	N	GG-I---KS--Q-KS-VE-
	Bacillus paramycoides	WP_265621927	-AQQ-IK--------	-	QG---R-KS--Q--SSVE-
	Alkalihalobacillus pseudalcaliphilus	WP_047990356	-EQ---ERN----DS	K	GG-I----S--E-SSY---

Figure S19: Partial sequence alignment of the rod shape-determining protein MreC showing a 1 aa insert (highlighted) in a conserved region that is generally exclusively shared by species from the *Anoxybacillus_B* clade. This CSI is also not shared by most other species from the family *Anoxybacillaceae* or other bacteria. A few exceptions in distantly related species are present.

Group	Species	Accession	165		207
Anoxybacillus_C	*Anoxybacillus caldiproteolyticus* DSM 15730	WP_181556046	LSLPLLYTMVGHF		GLPAPHFLMNPWVQFVLATPVQFYIGWPFY
	Anoxybacillus caldiproteolyticus 1A02591	WP_199426146	-------------		------------------------------
	Anoxybacillus caldiproteolyticus U458	WP_194520463	-------------		------------------------------
	Anoxybacillus calidus	WP_181536015	----------A-S	PWDL	---M--IF----F--I---L----------
Other *Anoxybacillaceae* (0/ >20)	*Anoxybacillus flavithermus*	MCG5027017	---------LA-M	PFDI	---M--L-----F-LL----------G---
	Anoxybacillus gonensis	WP_257637437	----------A-M	PFHI	---M--L-----F-LL----------G---
	Anoxybacillus ayderensis	MED0685337	----------A-M	PFHI	---M--L-----F-LL----------G---
	Anoxybacillus voinovskiensis	WP_183184909	----------A-M	PFDI	---M--L-----F-LL----------G---
	Anoxybacillus suryakundensis	WP_055441594	----------A-M	PFHI	---M--L-----F-LL----------A---
	Anoxybacillus tepidamans	WP_027409729	----------A-M	PFRI	---M--I-----F-LL----------G---
	Anoxybacillus thermarum	KIQ93185	---------LA-M	PFDI	---M--L-----F-LL----------G---
	Anoxybacillus vitaminiphilus	WP_111645935	---------IA-M	PFDI	---M--I-----F-LL----------G---
	Anoxybacillus rupiensis	WP_275191965	---------IA-M	PFDM	---M--I-----F-LL----------G---
	Anoxybacillus amylolyticus	ANB59957	---------LA-M	PFDI	---M--L-----F-LL----------G---
	Anoxybacillus kestanbolensis	WP_077428961	---------LA-M	PFDI	---M--L-----F-LL----------G---
	Anoxybacillus pushchinoensis	WP_091704857	---------IA-M	PFHI	---M-KL-----F--L----------G---
	Geobacillus stearothermophilus	KZM56131	---------LA-M	PFDI	---M--W-----F-LL----------G---
	Geobacillus kaustophilus	WP_307895682	---------LA-M	PFDI	---M--W-----F-LL----------G---
	Geobacillus subterraneus	WP_063166559	---------LA-M	PFDI	---M--L-----F-LL----------G---
	Geobacillus thermodenitrificans	WP_029760786	F--------IA-L	PFDL	---M--G-------LL----------G---
	Geobacillus thermoleovorans	WP_081130908	---------LA-M	PFDI	---M--W-----F-LL----------G---
	Parageobacillus thermantarcticus	WP_090949758	---------IA-M	PFNI	---M--L-----F-LL----------G---
	Parageobacillus yumthangensis	PDM40742	---------IA-M	PFDI	---M--M-----F-LL----------G---
	Parageobacillus caldoxylosilyticus	WP_042410200	---------IA-L	PFDL	---V-DW-------LLF---------G---
	Parageobacillus thermoglucosidasius	WP_013876861	---------IA-L	PFDL	---M-DW-------LLF---------G---
	Saccharococcus thermophilus	WP_166909130	---------IA-L	PFDL	---V-NW-------LLF-------V-G---
Unamed *Anoxybacillaceae* strains (0/ >20)	*Geobacillus genomosp. 3*	WP_020959825	---------IA-L	PFGL	---M--W-------LL----------G---
	Geobacillus sp. 46C-IIa	WP_081207412	---------IA-L	PLDL	---M--G-------LL----------G---
	unclassified Anoxybacillus	WP_237810821	----------A-M	PFHI	---M--L-----F-LL----------G---
	Anoxybacillus sp. EFIL	WP_171334978	----------A-M	PFHI	---M--L-----F-LL----------G---
	Anoxybacillus sp. MB8	WP_163151684	----------A-M	PFHI	---M--L-----F-LL----------G---
	Anoxybacillus sp. CHMUD	WP_171700024	---------IA-M	PFDI	---M--L-----F-LL----------G---
	Geobacillus sp. BMUD	WP_171660101	---------IA-M	PFDI	---M--M-----F-LL----------G---
	Parageobacillus sp. SY1	TXK91291	---------IA-M	PFDI	---M--M-----F-LL----------G---
	Geobacillus sp. 12AMOR1	AKM18181	---------LA-M	PFDI	---M--W-----F-LL----------G---
	Geobacillus sp. WSUCF-018B	WP_100664258	---------LA-M	PFDI	---M--W-----F-LL----------G---
	Geobacillus	WP_068895755	---------LA-M	PFAI	---M--L-----F-LL----------G---
	Anoxybacillus sp. CHMUD	WP_171700889	---------LA-M	PFDI	---M--L-----F-LL----------G---
	Geobacillus sp. LEMMJ02	WP_144329733	---------LA-M	PFDI	---M--L-----F-LL----------G---
	Geobacillus sp. FJAT-46040	WP_096225644	---------LA-M	PFDI	---M--L-----F-LL----------G---
	Anoxybacillus sp. J5B_2022	WP_268852854	---------LA-M	PFDI	---M--L-----F-LL----------G---
	Geobacillus sp. TFV-3	WP_160156868	---------LA-M	PFDI	---M--L-----F-LL----------G---
	Parageobacillus sp. SY1	TXK91800	---------IA-L	PFDL	---M-AW-------LL----------G---
	Geobacillus sp. E263	WP_143415483	---------IA-L	PFDL	---M-AW-------LL----------G---
	Parageobacillus sp. G301	WP_285753160	---------IA-L	PFDL	---M-AW-------LL----------G---
	Anoxybacillus sp. ST70	WP_019417822	----------A-M	PFHI	---M-NL-----F-LL----------G---

Figure S20: Partial sequence alignment of the heavy metal translocating P-type ATPase protein showing a 4 aa deletion (highlighted) in a conserved region that is exclusively shared by species from the *Anoxybacillus*_C clade. This CSI is also not shared by any other species from the family *Anoxybacillaceae* or other bacteria.

Group	Species	Accession	63		116
Anoxybacillus_C	*Anoxybacillus caldiproteolyticus DSM 15730*	WP_181556420	FAVTEEFSSYFPPSTLDAIQKNICQAWKGG	I	QYSGKRTFQEAVNTYKLGIYSAT
	Anoxybacillus caldiproteolyticus U458	WP_194519162	-------------S----------------	-	-----------------------
	Anoxybacillus caldiproteolyticus 1A02591	WP_199426596	------------------------------	-	----------------F------
Other *Anoxybacillaceae* (0/ >20)	*Anoxybacillus tepidamans*	WP_221277336	-S-----------N--E--------L---N		N----------------------
	Anoxybacillus vitaminiphilus	WP_111645554	--------T-------ET---K--EQ-S-F		D-G--------------------
	Anoxybacillus amylolyticus	WP_066322877	-S-----------NS-E---T------N-K		N-N-------TI--------C--
	Anoxybacillus rupiensis	WP_249218696	-S---D--A----Q-FE---QH---L-S-H		N-----------S----------
	Anoxybacillus voinovskiensis	WP_183185925	-S-----------NA-E---T------S-K		N-GE----------------C--
	Anoxybacillus calidus	WP_181537309	-SA-----T-------ET---K--EP-S-F		D-G-Q------------------
	Anoxybacillus ayderensis	WP_181519634	--A-----T----Q--E---Q---AN-R-K		N---A---A--ID----------
	Anoxybacillus thermarum	WP_043965014	--A-----T----R--E---Q---AN-R-K		N---A---A--ID----------
	Anoxybacillus tengchongensis	WP_183249849	--A-----T----Q--E---Q---AN-R-K		N-G-A---A--ID----------
	Thermolongibacillus altinsuensis	WP_132949023	--A-----P----T--ET-E-Q--AQ-S-K		N-G--------T-----------
	Anoxybacillus flavithermus	WP_006322170	--A-----T----Q--ET--Q---AN-R-K		N-G-A---A--ID----------
	Anoxybacillus mongoliensis	WP_183243956	--A-----T----Q--ET--Q---AN-H-K		N-G-A---A--ID----------
	Anoxybacillus kestanbolensis	WP_252506445	--A-----T----Q--ET--Q---AN-R-K		N-GRA---A--ID----------
	Anoxybacillus suryakundensis	WP_055441435	--A-----T----R--ET-EQHVRAN-R-K		N-G-A---A--ID----------
	Anoxybacillus pushchinoensis	WP_091700925	--E-----T----R--ET-EQHVRAN-R-K		N-G-V---RD--D-------C--
	Anoxybacillus tepidamans	WP_232223981	--I----------G-RE---Q--RTY-N-K		N---E--LEQ-LQ--QI---C-S
Unamed *Anoxybacillaceae* strains (0/ >20)	*Anoxybacillus sp. B2M1*	ANB56589	-S---D--A----Q-FE---QY---L-S-H		N-----------S----------
	unclassified Anoxybacillus	WP_052661008	-S---D--A----Q-FE---QY---L-S-H		N-----------S----------
	Anoxybacillus sp. P3H1B	KXG09649	-S---D--A----Q-FE---QH---L-S-H		N-----------S----------
	Anoxybacillus sp.	WP_290908000	-SA----------NA-E--HT----T-H-T		S-------------------C--
	Anoxybacillus sp. J5B_2022	WP_268852423	-SA----------T--E---TT-Y-V-N-S		N-------------------C--
	Anoxybacillus sp. ST70	WP_220382690	--A-----T----Q--ET--Q---AN-R-K		N--KA---A--ID----------
	uncultured Anoxybacillus sp.	WP_297990911	--A-----T----R--ET-EQHVRAN-R-K		N-G-V---RD--D-------C--

Figure S21: Partial sequence alignment of the DUF2225 domain-containing protein showing a 1 aa insert (highlighted) in a conserved region that is exclusively shared by species from the *Anoxybacillus*_C clade. This CSI is also not shared by any other species from the family *Anoxybacillaceae*.

Group	Species	Accession	102		141
Brevibacillus (28/ 28)	*Brevibacillus brevis*	WP_064200419	CPTNITSVEQKAIREAAERSGG	A	REVFIEEEPKVAAVGAG
	Brevibacillus fulvus	WP_204517879	----------------------	-	-----------------
	Brevibacillus aydinogluensis	MDT3417822	----------------------	-	-----------------
	Brevibacillus antibioticus	TKI58551	----------------------	-	-----------------
	Brevibacillus migulae	WP_134683445	----------------------	-	-------------I---
	Brevibacillus dissolubilis	WP_139491111	----------------------	-	---Y-------------
	Brevibacillus massiliensis	WP_019121104	--------------------A-	-	-----------------
	Brevibacillus marinus	WP_126428825	--------------------C-	-	---YL------------
	Brevibacillus laterosporus	WP_113756261	----------------------	-	-------------I---
	Brevibacillus gelatini	WP_122902813	----------------------	-	-Q---------------
	Brevibacillus parabrevis	WP_063229019	----------------------	-	-Q---------------
	Brevibacillus choshinensis	WP_055745663	----------------------	-	---Y-------------
	Brevibacillus centrosporus	WP_092268249	----------------------	-	---Y-------------
	Brevibacillus nitrificans	WP_122926912	----------------------	-	---Y-------------
	Brevibacillus fluminis	WP_122920599	----------------------	-	---Y-------------
	Brevibacillus reuszeri	WP_103109788	----------------------	-	---Y-------------
	Brevibacillus invocatus	WP_251242632	----------------------	-	---Y-------------
	Brevibacillus borstelensis	WP_003388382	----------------------	-	-----------------
	Brevibacillus composti	WP_198827777	----------------------	-	-----------------
	Brevibacillus panacihumi	WP_023555223	----------------------	-	-----------------
	Brevibacillus humidisoli	WP_230043601	----------------------	-	---YL-----I------
	Brevibacillus daliensis	WP_232697874	---D------------------	-	-------------I---
	Brevibacillus ruminantium	WP_251872474	----------------------	-	-----------------
	Brevibacillus thermoruber	MDA5108132	----------------------	-	-----------------
	Brevibacillus halotolerans	MBA4531840	----------------------	-	-------------I---
	Brevibacillus fortis	PSJ94879	----------------------	-	-----------------
	Brevibacillus formosus	KLH99007	----------------------	-	-----------------
	Brevibacillus agri	MBY0051223	----------------------	-	-Q---------------
A.sediminis (1/ 1)	*Anoxybacillus sediminis*	WP_171564130	----------------------	-	-----------------
Unnamed Brevibacillus strains	*Brevibacillus sp. BC25*	WP_007722070	----------------------	-	-----------------
	Brevibacillus sp. SYP-B805	WP_165212388	------P---------------	-	-------------I---
Other *Anoxybacillaceae* (0/ >15)	*Anoxybacillus pushchinoensis*	WP_091704120	---------R---K----K---		KK-YL--------I---
	Anoxybacillus amylolyticus	WP_066322661	---------R---K----K---		KK-YL--------I---
	Anoxybacillus ayderensis	WP_181519478	---------R---K----K---		KK-YL--------I---
	Anoxybacillus calidus	WP_181537067	---------R---K----K---		KK-YL--------I---
	Anoxybacillus flavithermus	WP_012576168	---------R---K----K---		KK-YL--------I---
	Anoxybacillus gonensis	WP_009361902	---------R---K----K---		KK-YL--------I---
	Anoxybacillus mongoliensis	WP_183242450	---------R---K----K---		KK-YL--------I---
	Anoxybacillus suryakundensis	WP_055440763	---------R---K----K---		KK-YL--------I---
	Anoxybacillus tengchongensis	WP_183247552	---------R---K----K---		KK-YL--------I---
	Anoxybacillus tepidamans	WP_027407998	---------R---K----K---		KK-YL--------I---
	Anoxybacillus vitaminiphilus	WP_111644721	---------R---K----K---		KK-YL--------I---
	Anoxybacillus voinovskiensis	WP_183183310	---------R---K----K---		KK-YL--------I---
	Geobacillus icigianus	WP_033018257	----T----R---K----K---		KK-YL--------I---
	Geobacillus jurassicus	WP_066232662	----T----R---K----K---		KK-YL--------I---
	Geobacillus kaustophilus	WP_044732801	----T----R---K----K---		KK-YL--------I---
	Geobacillus stearothermophilus	WP_289667120	----T----R---K----K---		KK-YL--------I---
	Geobacillus subterraneus	WP_322310652	----T----R---K----K---		KK-YL--------I---
	Parageobacillus caldoxylosilyticus	WP_244380448	----T----R---K----K---		KK-YL--------I---
	Parageobacillus thermantarcticus	WP_090949538	----T----R---K----K---		KK-YL--------I---
	Parageobacillus toebii	WP_062754390	--S-T----R---K----K---		KKIYL--------I---
	Thermolongibacillus altinsuensis	WP_132947363	------AI-R---K----K---		KK-YL--------I---
Other Bacteria (2/ >90)	*Listeria booriae*	WP_185610290	----------------------		KQ--M--------I---
	Bacillus cereus	WP_199710995	----------------------		KT--L------S-----
	Paenibacillus contaminans	WP_113034222	---------------------A		----L--------I---
	Escherichia coli	HBE2402071	------------------K---		KK-YL--------I---
	Viridibacillus soli	WP_100794821	------------------K---		-N--L--------I---
	Listeria weihenstephanensis	WP_036059655	--------------D-------		KQ--M--------I---
	Jeotgalibacillus aurantiacus	WP_227394441	------------------K---		KR--L--------I---
	Rubeoparvulum massiliense	WP_048601475	-----------------Q---A		-T--L------------
	Priestia megaterium	RBN42345	------------------K---		KTIYL--------I---
	Jeotgalibacillus proteolyticus	WP 104058573	------------------K---		KR--L--------I---
	Viridibacillus arvi	WP_053417878	------------------K---		-N--L--------I---
	Bacillus thuringiensis	WP_193412727	----------------------		KT--L------------
	Bacillus paranthracis	WP_258431292	----------------------		KT--L------------
	Jeotgalibacillus malaysiensis	WP_039811500	------------------K---		KRI-L--------I---
	Brevibacterium sp. JNUCC-42	QOS98180	----------------------	-	-------------I---
	Mesorhizobium sp. MOO.F.Ca.ET.186.01.	TGV29875	----------------------	-	-Q---------------

Figure S22: Partial sequence alignment of the rod shape-determining protein showing a 1 aa insert (highlighted) in a conserved region that is generally exclusively shared by *A.sediminis* and species from the genus *Brevibacillus*. This CSI is also not shared by most other species from the family *Anoxybacillaceae* or other bacteria. A few exceptions in distantly related species are present.

Group	Species	Accession	162		205
			IGGKNVYSFGIRSGMKEEFEWAKEN		MHLYKYDVLEPVKQVLPTI
Brevibacillus (23/ 23)	*Brevibacillus brevis*	WP_016740148	IGGKNVYSFGIRSGMKEEFEWAKEN		MHLYKYDVLEPVKQVLPTI
	Brevibacillus panacihumi	WP_122914909	-------------------------		-------------------
	Brevibacillus fluminis	WP_122920532	-------------------------		-------------------
	Brevibacillus migulae	WP_134683375	-------------------------		-------------------
	Brevibacillus invocatus	WP_251242667	-------------------------		-------------------
	Brevibacillus composti	WP_198827849	-------------------------		-------------------
	Brevibacillus borstelensis	WP_039071513	-------------------------		-------------------
	Brevibacillus parabrevis	WP_063228946	---------------R---------		-------------------
	Brevibacillus reuszeri	WP_103104668	---------------R---------		-------------------
	Brevibacillus laterosporus	WP_113756323	L------------------------		-------------------
	Brevibacillus centrosporus	WP_092268317	---------------R---------		---T-F-------K-----
	Brevibacillus nitrificans	WP_122926501	---------------R---------		---T-F-------K-----
	Brevibacillus marinus	WP_126428956	---E----------T----V----H		-------------------
	Brevibacillus massiliensis	WP_019121034	-------------------L-----		-N-----------S---E-
	Brevibacillus fortis	WP_106839674	----------------D--------		-------------------
	Brevibacillus dissolubilis	WP_139491035	-------------------------		-----F-------------
	Brevibacillus fulvus	WP_204517978	-------------------A-----		-------------------
	Brevibacillus choshinensis	WP_055745733	---------------R---------		-------------K-----
	Brevibacillus daliensis	WP_232697941	-------------------------		-------------------
	Brevibacillus humidisoli	WP_230047067	-------------------------		-----------I-------
	Brevibacillus formosus	WP_106779449	----------------D--------		-------------------
	Brevibacillus ruminantium	WP_251872546	M------------------------		------------------L
	Brevibacillus gelatini	WP_122902886	-------------------D-----		-N-----------------
A.sediminis (1/ 1)	*Anoxybacillus sediminis*	WP_171564032	-------------------------		-------------------
Unnamed *Brevibacillus* strains	*Brevibacillus*	WP_029100425	-------------------------		-------------------
	Brevibacillus sp.	MBO8165359	---R----------T----------		------------------V
	Brevibacillus sp. SYP-B805	WP_165213515	-------------------------		-------------------
	Brevibacillus sp. HB1.3	WP_172139225	----------------D--------		-------------------
	Brevibacillus sp. SKDU10	WP_064016432	L------------------D-----		-------------------
	Brevibacillus sp. M2.1A	WP_173610428	----------------D------G-		-------------------
	unclassified Brevibacillus	WP_007724780	----------------D--------		-------------------
Other *Anoxybacillaceae* (4/ >10)	*Anoxybacillus pushchinoensis*	WP_091700098	--P--------------------Q-		-YIA-F-----L-E----L
	Anoxybacillus mongoliensis	WP_183242532	--P----------------------		-YIA-F-----L-E----L
	Anoxybacillus flavithermus	AST05999	--P--------------------Q-		-YIA-F-----L-E----L
	Anoxybacillus ayderensis	WP_021094345	--P--------------------Q-		-YIA-F-----L-E----L
	Anoxybacillus kamchatkensis	WP_181519450	--P--------------------Q-	G	-YIA-F-----L-E----L
	Anoxybacillus thermarum	WP_043967280	--P--------------------Q-	G	-YIA-F-----L-E----L
	Anoxybacillus voinovskiensis	WP_183183245	--P----------------------	G	-YIA-FE----L-E----L
Other Bacteria (4/ >90)	*Planococcus maritimus*	WP_274843323	--P---------------------Q	G	-----FE----L------L
	Bhargavaea ginsengi	WP_092054617	--PH------------------R--	G	--IS-F-----L------L
	Planococcus koreensis	WP_135500826	--P----------------------	G	---S-FE----L------L
	Neobacillus vireti	WP_024027287	--P----------------------	G	--IS-F-----L-EI---L
	Edaphobacillus lindanitolerans	WP_076756823	--PR------------------R--	G	---S-F-----LRE---AL
	Lysinibacillus capsici	WP_225340673	--P----------------------	G	--IS-FE----L-E-----
	Bacillus subtilis	WP_019846779	--PH---------------------	G	--IS-FE----L-E---KL
	Bhargavaea cecembensis	WP_063179987	--PR------------------R--	S	--IS-F-----L-E---AL
	Neobacillus soli	WP_066073702	--P--------------------Q-	G	--IS-F-----L-EI---L
	Planococcus halocryophilus	WP_240749100	--P----------------D-----	G	---S-FE----L-E----L
	Lysinibacillus sphaericus	WP_223556860	--P----------------------	G	--IS-FE----L-E----L
	Lysinibacillus varians	WP_025218564	--P----------------------	G	--IS-FE----L-E----L
	Planococcus faecalis	WP_078080675	--P----------------D-----	G	---S-FE----L-E----L
	Lysinibacillus fusiformis	WP_241605598	--P----------------------	G	--IS-FE----L-E----L
	Lysinibacillus cavernae	WP_155593588	--P----------------------	G	--IS-FE----L-E----L
	Lysinibacillus contaminans	WP_053585225	--P----------------------	G	--IS-FE----L-E----L
	Bacillus ectoiniformans	WP_204553633	--PS---------------------	G	--IS-F-----L-EI---L
	Planococcus versutus	WP_049692963	--P----------------------	G	--IS-FE----L-K----L
	Jeotgalibacillus proteolyticus	WP_104058673	--P----------------------	G	--IS-FE----L-EI---L
	Jeotgalibacillus soli	WP_041086932	--P----------------------	G	--IS-FE----L-EI---L
	Bacillus siamensis	PIK32614	--PQ--F------------------	G	--IS-FE----L-K---QL
	Streptococcus pneumoniae	CEX85688	--PE--------------------V	G	-N---F-----L-E---KL
	Niallia taxi	WP_127738976	--P---------------------V	G	--IS-F-----L-EI---L
	Planococcus donghaensis	WP_065525487	--P----------------D-----	G	--IS-FE----L-E----L
	Bacillus cytotoxicus	WP_251227975	--PE--------------------V	G	-N---F-----L-K----L
	Psychrobacillus soli	WP_142605289	--P----------------D-----	G	--IS-FE----L-E----L
	Psychrobacillus lasiicapitis	WP_142537985	--P----------------D-----	G	--IS-FE----L-E----L
	Ammoniphilus sp. YIM 78166	WP_134702442	M-------------LR---A-----		-N-F-F-----L-------
	Ammoniphilus sp. CFH 90114	WP_129199259	M-------------LR---A-----		-N---F-----L-K-----
	Ammoniphilus resinae	WP_209811992	---------------R---Y-----		-N-F-F-----L-K---S-
	Ammoniphilus oxalaticus	WP_120190512	--------------T--------KH		-N-F-F---A-LQK----L

Figure S23: Partial sequence alignment of the agmatinase protein showing a 1 aa deletion (highlighted) in a conserved region that is generally specific for *A.sediminis* and species from the genus *Brevibacillus*. This CSI is also not shared by most other species from the family *Anoxybacillaceae* (except for 4 *Anoxybacillus* species) or other bacteria. A few exceptions in distantly related species are present.

Group	Species	Accession	103		143
Brevibacillus (20/ 20)	*Brevibacillus brevis*	RED27321	FKDAGLVTELTYGDASSRFDANVE	E	DHYHAICSSCGAIRDF
	Brevibacillus borstelensis	WP_003391638	-------R----------------	-	--------E--S----
	Brevibacillus composti	WP_198827372	-------R----------------	-	--------E--S----
	Brevibacillus fulvus	WP_204517544	-------R---------------A	-	-------TK--S-Q--
	Brevibacillus reuszeri	WP_049736954	------------------------	-	----------------
	Brevibacillus choshinensis	WP_055747419	-------V----------------	-	--------K----S--
	Brevibacillus invocatus	WP_122911242	-------R----------------	-	-------MN--S-S--
	Brevibacillus gelatini	WP_122906720	-------I------S---------	-	----------------
	Brevibacillus panacihumi	WP_031305571	-------R----------------	-	-------IH--S-S--
	Brevibacillus laterosporus	WP_104033823	--E----R----------------	D	E---I---E----Q--
	Brevibacillus migulae	WP_134686832	-------R--------------IV	-	--------E---VH--
	Brevibacillus dissolubilis	WP_139492389	-------R----------------	-	E------NE--S-H--
	Brevibacillus fluminis	WP_122915949	-------K----------------	-	E---V---G--V----
	Brevibacillus massiliensis	WP_173391410	--E----R------S-------TH	-	E---V---E----H--
	Brevibacillus marinus	WP_126425371	--E----R--------------TM	-	E---I---E--D----
	Brevibacillus agri	ELK43525	-------I----------------	-	--------N-------
	Brevibacillus thermoruber	WP_029100587	--E----R----------------	-	-------TE-------
	Brevibacillus humidisoli	WP_230044928	--E----R--------------TM	-	E---I---K--D----
	Brevibacillus daliensis	WP_232697837	-------R----------------	-	E---I---D--S-H--
	Brevibacillus ruminantium	WP_251871826	-------R----------------	-	--------E--S----
A.sediminis (1/ 1)	*Anoxybacillus sediminis*	WP_212132677	--E----R----------------	-	-------TK-------
Unnamed *Brevibacillus* strains	*Brevibacillus sp. MCWH*	WP_171565028	--E----R----------------	-	-------TK-------
	Brevibacillus sp. SYP-B805	WP_165215291	--E----R--------------IS	-	--------E---VH--
	Brevibacillus sp. CF112	EJL39555	-------I----------------	-	--------N-------
	Brevibacillus sp. BC25	WP_039961469	------------------------	-	--------T----S--
	Brevibacillus sp. SKDU10	WP_064018839	--E----R----------------	D	E---I---E----Q--
Other Bacteria (1/ >100)	*Risungbinella massiliensis*	WP_044641066	--E----R------S-------LM		----V--TN--K-T--
	Numidum massiliense	WP_054951725	-------R---------------S		-----------K-E--
	Lihuaxuella thermophila	WP_089971944	--E----R--------------MD		E---V--RK--K-T--
	Novibacillus thermophilus	WP_077719804	--E----R------S---Y---TV		-------TQ--K-K--
	Aneurinibacillus tyrosinisolvens	WP_047155535	--ET---R--------------I-		E----V-VK-NKVE--
	Planifilum fimeticola	WP_211295706	--E----R--------------MS		----V--RR--K-T--
	Baia soyae	WP_131848516	------IR----------------		----V--QT--K-T--
	Paenactinomyces guangxiensis	WP_181752814	--E----R--------------MG		E---V---R--K-T--
	Melghirimyces profundicolus	PTX60702	--E----R--------------M-		E---I--RE--R-V--
	Thermoflavimicrobium dichotomicum	WP_093226905	--E----R--------------MT		E---V--KQ--K-T--
	Rubeoparvulum massiliense	WP_048600699	--EN---R----------Y---MT		E---V--TE----H--
	Thermoflavimicrobium daqui	RAL24308	-------R--------------MT		E---V--KK--K-T--
	Polycladomyces abyssicola	BCU82909	--E----R--------------MT		----V--RE--K-T--
	Calditerricola satsumensis	WP_054672802	--E----R------N-------MS		----V--T---K-V--
	Seinonella peptonophila	WP_073155775	--E----R--------------MS		----V--NH--K-T--
	Laceyella sacchari	WP_022735699	--E----R--------------MM		----V--RE--K-T--
	Melghirimyces algeriensis	WP_185956229	--E--M-R--------------M-		E---I--RE--K-V--
	Planifilum fulgidum	WP_092040483	--E----R------S-------MS		----I--RR--K-T--
	Paenibacillus dendritiformis	WP_168181592	-IE----R------S--------S		-------EQ--K-V--
	Paenibacillus thiaminolyticus	WP_119791869	-IE----R------S--------S		-------EQ--K-V--
	Paenibacillus popilliae	WP_006285165	-IE----R------S--------S		-------EQ--K-I--
	Desmospora activa	WP_107727754	--E----R--------------MH		E---I--RE--K-V--
	Marinithermofilum abyssi	WP_188648861	--E----R------S-------MS		----V--RE--K-V--
	Kroppenstedtia pulmonis	WP_173220212	--E----R--------------MD		----I--RD--K-V--
	Staphylospora marina	WP_124726557	--E----K------S-------MM		----V--RK--K-T--
	Kroppenstedtia guangzhouensis	GGA51050	--E--M-R--------------MK		E---V--RE--K-V--
	Paenibacillus tengchongensis	WP_151737459	-ME--M-R------S--------S		----V--QE--K-E--
	Shimazuella alba	WP_160802921	-RE----R--------------MD		----V--HN--H-T--
	Shimazuella kribbensis	WP_028778421	-RE---IR--------------MN		----V--HH--H-T--
	Calidifontibacillus oryziterrae	WP_017754307	--EV---R------S-----C--S		-------EQ--K-V--
	Paenibacillus etheri	WP_060625695	-ME--M-R------S--------S		----V--QQ--K-E--
	Kroppenstedtia eburnea	QKI83199	--E--M-R--------------MT		E---I--RE--K-V--
	Paenibacillus rhizophilus	WP_124697006	-ME--M-R------N--------T		----I--QK--K-E--
	Aneurinibacillus danicus	WP_146812456	---V---R----------Y---MS		-----V-IH--MVK--
	Caldalkalibacillus thermarum	WP_188623755	--E----H----------------		A---V--RE--K-V--
	Paenibacillus dakarensis	WP_054957391	-IE--M-R------N--------S		----V--EK--K-E--
	Paludifilum halophilum	WP_094265239	--E----R------S-------MN		----I--RD--K-V--
	Paenibacillus assamensis	WP_169449364	-IE----R------S------DIS		-------EK--K-A--
	Shimazuella soli	WP_240877051	-RE----R--------------MD		----V--HY--H-T--
	Paenibacillus gallinarum	WP_191804064	-TQ--M-R------N-------IS		E---I--EK--T-K--
	Paenibacillus taiwanensis	WP_028544570	-IE----H------S--------S		-------EK--K-A--
	Paenibacillus apiarius	WP_087434967	-IE----R------S------D-S		-------EQ--K-V--
	Paenibacillus luteus	WP_141502739	-VE----R------D-----S-LS		-------K---S-V--
	Brevibacterium sp. JNUCC-42	QOS97605	--E----R----------------	D	E---I---E----Q--

Figure S24: Partial sequence alignment of the Fur family peroxide stress response transcriptional regulator protein showing a 1 aa insert (highlighted) in a conserved region that is generally exclusively shared by *A.sediminis* and species from the genus *Brevibacillus*. This CSI is also not shared by most other species from the family *Anoxybacillaceae* or other bacteria. One exception in a distantly related species is present.

Group	Species	Accession	146		180
Brevibacillus (28/ 28)	*Brevibacillus brevis*	WP_188066926	FEVKANY	S	GNRSLTKQKIWLTEDFKPTHAEIMDAS
	Brevibacillus fulvus	WP_204519291	-------	-	---M--R------------R-----TN
	Brevibacillus borstelensis	WP_003389792	-------	-	---------------L--KR-----SN
	Brevibacillus invocatus	WP_122910234	-------	-	-------------------------S-
	Brevibacillus panacihumi	WP_122912439	-------	-	-------------------Q-----S-
	Brevibacillus centrosporus	WP_122960119	-------	-	-------------------------S-
	Brevibacillus choshinensis	WP_203355164	-------	-	-------------------------S-
	Brevibacillus nitrificans	WP_122925717	-------	-	-------------------------S-
	Brevibacillus migulae	WP_134685619	-------	-	-----------M-------K------N
	Brevibacillus fluminis	WP_122916879	-------	-	-----A-----M-------Q-----NN
	Brevibacillus massiliensis	WP_019123945	-------	-	-----------MS-----AK-----SN
	Brevibacillus formosus	WP_047074007	-------	-	---------------------------
	Brevibacillus fortis	WP_106841961	-------	-	---------------------------
	Brevibacillus composti	WP_198828441	-------	-	------------SD-L--KR-----SN
	Brevibacillus porteri	WP_106835608	-------	-	---------------------------
	Brevibacillus antibioticus	WP_137031090	-------	-	---------------------------
	Brevibacillus dissolubilis	WP_139491925	-------	-	-----------M-------Q-----NN
	Brevibacillus agri	WP_242507426	-------	T	-------------------------S-
	Brevibacillus reuszeri	WP_103108042	-------	-	-------------------------S-
	Brevibacillus parabrevis	WP_063229455	-------	-	-------------------------S-
	Brevibacillus humidisoli	WP_230044446	-------	-	------R------------------GN
	Brevibacillus ruminantium	WP_251873492	-------	-	------------S--L--KR-----SN
	Brevibacillus daliensis	WP_232699912	-------	-	----FS-------DELI-KK-----NN
	Brevibacillus gelatini	WP_122905449	-------	T	-------------------------S-
	Brevibacillus halotolerans	WP_031411760	-------	-	----FS-----MSN-LR--R-----NN
	Brevibacillus laterosporus	WP_018673806	-------	-	----FS-----MSN-LR-MR-----NN
	Brevibacillus thermoruber	WP_029100999	-------	N	------R-------EL---R-----SN
	Brevibacillus marinus	WP_126425052	-------	-	-------------------------GN
A.sediminis (1/ 1)	*Anoxybacillus sediminis*	WP_171566173	-------	-	------R-------EL---R-----SN
Unnamed *Brevibacillus* strains	*Brevibacillus sp.*	MBO8164050	-------	-	------R------------R------N
	Brevibacillus sp. SYP-B805	WP_165213231	-------	-	-----------M-------K-----SN
	Brevibacillus sp. DP1.3A	WP_173621363	-------	-	---------------------------
	Brevibacillus sp. BC25	WP_007721096	-------	-	---------------------------
	Brevibacillus sp. Leaf182	WP_056488781	-------	-	---------------------------
	Brevibacillus sp. HB2.2	WP_173607087	-------	-	---------------------------
	Brevibacillus sp. SKDU10	WP_064019187	-------	-	----FS-----MSN-LR--R-----NN
Other Bacteria (4/ >15)	*Bacillaceae bacterium*	MBO8171640	-----D-		Q-----S--------LL-SRV-V--TD
	Ammoniphilus resinae	WP_245203465	-----D-		Q----DT--V--N--LS-VRV-V--TN
	Aneurinibacillus thermoaerophilus	QYY43165	-----D-		Q-----T---V-NKSLE-VRVDV--SN
	Aneurinibacillus terranovensis	WP_027416541	-----D-		L-----T---T-DKSLD-VRV--R-SN
	Aneurinibacillus aneurinilyticus	WP_021623785	-----D-		Q-----S---T-NKSLE-VRVDV--TN
	Aneurinibacillus migulanus	KIV59728	-----D-		Q-----S---T-NKSLE-VRVDV--TN
	Aneurinibacillus tyrosinisolvens	WP_047154857	-Q---D-		Q-----T---T-NKSLD-LRVDV--SN
	Aneurinibacillus soli	WP_096467538	-----E-		Q-----S-R-V-NKGLT-QKV-V--SN
	Paenibacillaceae bacterium	HBI05459	-T-----		P-Q---T---IFDKAL--KKV----SN
	Caldalkalibacillus thermarum	GGK26838	-DTA---		H-KT-KH-R---DK-L---KVHL---D
	Anoxybacillus sp. LAT_38	MCG6197405	-------	N	------R-------EL---R-----SN
	Brevibacterium sp. JNUCC-42	QOS98828	-------	-	----FS-----MSN-LR-KK-----NN
	Pasteuria penetrans	WP_149453057	-D-V---	-	VFPQ--R-----DKEL--RKI--R-E-
	Marininema mesophilum	WP_091741931	---A---	P	Q-Q--KY-R--MN--YH-KKV-VL--E

Figure S25: Partial sequence alignment of the outer membrane lipoprotein-sorting protein showing a 1 aa insert (highlighted) in a conserved region that is generally exclusively shared by *A.sediminis* and species from the genus *Brevibacillus*. This CSI is also not shared by most other species from the family *Anoxybacillaceae* or other bacteria. A few exceptions in distantly related species are present.

Group	Species	Accession	104		139
Brevibacillus (24/ 24)	*Brevibacillus brevis*	WP_048031337	LDISRELCMNNMRRILDSAKKNGN	I	FVRIDMEDYAH
	Brevibacillus invocatus	WP_122909172	-----D-----------V------	-	-----------
	Brevibacillus massiliensis	WP_019121505	-----D--LH-------A------	-	-----------
	Brevibacillus migulae	WP_134685717	----KD-----------T------	-	-----------
	Brevibacillus fluminis	WP_122916047	-----D--VT--------------	-	-----------
	Brevibacillus panacihumi	WP_122913505	-----------------A---F--	-	-----------
	Brevibacillus gelatini	WP_122903305	-----D---T-------T------	-	-----------
	Brevibacillus formosus	WP_219663232	---------D--------------	-	-----------
	Brevibacillus choshinensis	WP_055747516	---------S-------A-R----	-	-----------
	Brevibacillus fortis	WP_106838275	---------E--------------	-	-----------
	Brevibacillus centrosporus	WP_092267097	---------Q-------A--A---	-	-----------
	Brevibacillus nitrificans	WP_122924988	---------Q-------A--A---	-	-----------
	Brevibacillus antibioticus	WP_137030708	---------D----------N---	-	-----------
	Brevibacillus dissolubilis	WP_139492319	----Y------------T--RH--	-	-----------
	Brevibacillus laterosporus	WP_104032698	-----D--VS-------T--QC--	-	-----------
	Brevibacillus reuszeri	WP_049736814	-----D---T----T--V--NL--	-	-----------
	Brevibacillus thermoruber	WP_044900347	-----------------A------	-	-----------
	Brevibacillus daliensis	WP_232697750	-----D--VS----V--A--RCD-	L	-----------
	Brevibacillus ruminantium	WP_251871954	--------I-------EF-RDH--	-	-----------
	Brevibacillus humidisoli	WP_230044807	---------D-------A--RY--	N	-----------
	Brevibacillus fulvus	WP_204517488	-----D--VA-------T---C--	M	-----------
	Brevibacillus borstelensis	WP_172920615	---------T-------Y--NH--	-	-----------
	Brevibacillus composti	WP_198827454	----------------QF-REH--	-	-----------
	Brevibacillus marinus	WP_126425312	--------VG-------A--RF--	N	-----------
A.sediminis (1/ 1)	*Anoxybacillus sediminis*	WP_230077779	-----D-----------A------	-	-----------
Unnamed *Brevibacillus* strains	*Brevibacillus sp.*	MBO8164596	---------T-------A--QY--	N	-----------
	Brevibacillus sp. SYP-B805	WP_165213969	-----D-----------V------	-	-----------
	Brevibacillus sp. BC25	WP_007722740	---------D--------------	-	-----------
	Brevibacillus sp. DP1.3A	WP_173618109	---------D--------------	-	-----------
	Brevibacillus sp. RS1.1	WP_173627842	---------D--K-----------	-	-----------
	Brevibacillus sp. SKDU10	WP_064018887	-----D--VS-------T--QC--	-	-----------
	Brevibacillus sp. 7WMA2	WP_163246886	-----D--VG-------T--QC--	-	-----------
	unclassified Brevibacillus	WP_171565100	-----D-----------A------	-	-----------
Other Bacteria (3/ >50)	*Lihuaxuella thermophila*	SEN28584	----K------------R--QY--		-----------
	Paenibacillus taiwanensis	WP_028544048	---------S---Q---A--QH--		-----------
	Effusibacillus lacus	WP_096181978	-----D---S-------R-SQY--		-----------
	Alicyclobacillus montanus	WP_072873256	----QD--LD--H----T---H--		-----------
	Thermoflavimicrobium daqui	WP_113659018	----KD---E---Q--NR--EY--		-----------
	Paenibacillus albiflavus	WP_132417826	---------Q---K---T-S-HQI		-----------
	Microaerobacter geothermalis	WP_236408388	----KD---K------ET---H--		-----------
	Effusibacillus pohliae	WP_018131577	----K----D-------R--QYD-		-----------
	Paenibacillus phytorum	WP_171647463	-----D--V---K----C-RQH--		-----------
	Aneurinibacillus terranovensis	WP_027417939	---DQN---S-------A-R-Y--		-----------
	Paenibacillus alginolyticus	WP_268617571	--------V---K----C-RQH--		-----------
	Robertmurraya korlensis	WP_066054304	-----D--VK--K----R--MY--		-----------
	Priestia abyssalis	WP_078414652	----K----K-------R--MH--		-----------
	Laceyella tengchongensis	MRG28449	----K----K-------C--RLN-		---------E-
	Paenibacillus aceris	WP_167068416	-----D--VS-------R-RMH--		-----------
	Laceyella sacchari	WP_022738341	----K----K-------C--RLN-		---------E-
	Priestia aryabhattai	WP_193426718	----K----E------HT-RM-NI		---------L-
	Paenibacillus apiarius	WP_206094081	-----D--LS---S--EA-SRYD-		-----------
	Paenibacillus elgii	WP_108533984	---DK----S-------R--EY--		-----------
	Alicyclobacillus tolerans	WP_272908264	--------ID--H----R-RTYH-		-----------
	Paenibacillus germinis	WP_171690469	----K---V---K---NR-R-Y--		-----------
	Paenibacillus dendritiformis	WP_168178797	--L------R---A---T-AAC--		-----------
	Cohnella endophytica	WP_120975063	----AD--LS--K----R-RQY--		----------R
	Peribacillus saganii	WP_117327868	-----DF-VA---K---T---TN-		---------S-
	Alicyclobacillus herbarius	WP_026961059	----ED---R-------T--SH--		S-N-------R
	Lysinibacillus sp. SDF0063	TQR31475	---------D--------------	-	-----------
	Longirhabdus pacifica	WP_128895797	---DYD--LKH-SM---A--TY--	N	-----------
	Brevibacterium sp. JNUCC-42	QOS97666	-----D--VS-------T--QC--	-	-----------

Figure S26: Partial sequence alignment of the proline dehydrogenase protein showing a 1 aa insert (highlighted) in a conserved region that is generally exclusively shared by *A.sediminis* and species from the genus *Brevibacillus*. This CSI is also not shared by most other species from the family *Anoxybacillaceae* or other bacteria. A few exceptions in distantly related species are present.

Group	Species	Accession	206		240
Brevibacillus (18/ 18)	*Brevibacillus brevis*	WP_106655671	LPQQAVVTGTLADIEERL	A	QNSHITNPAIILVGDV
	Brevibacillus thermoruber	WP_029097830	--E--T------T---TV	Q	RHPG-S----------
	Brevibacillus invocatus	WP_122910780	--D--T---------Q--	-	E-P-------------
	Brevibacillus borstelensis	WP_194247168	M-E-TT------------	-	AGP--S----------
	Brevibacillus reuszeri	WP_049737260	--N-TT-----EN----V	-	E-PR-S----------
	Brevibacillus panacihumi	WP_122912137	--E--T---------Q--	-	D-PQ----------E-
	Brevibacillus composti	WP_198827233	--E-TT-----E------	-	SGPK-S--------E-
	Brevibacillus fortis	WP_106842042	------------------	-	----------------
	Brevibacillus porteri	WP_106836444	------------------	-	----------------
	Brevibacillus antibioticus	WP_137030382	------------------	-	----------------
	Brevibacillus agri	WP_005832017	--K---------------	-	E---------------
	Brevibacillus parabrevis	WP_274874976	--K---------N-----	-	E---V-----------
	Brevibacillus formosus	WP_088906250	------L-----------	-	E---------------
	Brevibacillus gelatini	WP_122906569	-----------T---D--	-	E---------------
	Brevibacillus ruminantium	WP_251871639	M-E-TT------------	-	AGP--S----------
	Brevibacillus centrosporus	WP_092266228	----VTL-----------	-	GHREF---------N-
	Brevibacillus choshinensis	WP_055747222	----VTL----EN----M	-	T--Q------------
	Brevibacillus nitrificans	WP_122924249	----VTL-----------	-	GHRE----------N-
A.sediminis (1/ 1)	*Anoxybacillus sediminis*	WP_230076604	--E--T---S--T-V-TV	Q	RHPG-S----------
Unnamed *Brevibacillus* strains	*Brevibacillus sp. WF146*	WP_065067889	--E--T------T---TV	Q	RHPG-S----------
	Brevibacillus sp. LEMMJ03	WP_143862420	--E--T------T---TV	Q	RHPG-S----------
	Brevibacillus sp. BC25	WP_007725969	----------------H-	-	E---------------
	Brevibacillus sp. HB1.4B	WP_173623167	------------------	-	----------------
	Brevibacillus sp. AG	WP_271755992	------------------	-	----------------
	Brevibacillus sp. AG162	WP_142064286	------------------	-	----------------
	Brevibacillus sp. HB1.1	WP_174225384	------------------	-	----------------
	Brevibacillus sp. HB2.2	WP_173606162	------------------	-	--------------E-
	Brevibacillus sp. HB1.3	WP_172141987	------------------	-	--------------E-
	Brevibacillus sp. HD1.4A	WP_173596050	--K---------N-----	-	E---V-----------
	Brevibacillus sp. DP1.3A	WP_173620104	------------------	-	E---------------
	Brevibacillus sp. M2.1A	WP_173608961	-----------V------	-	----------------
	unclassified Brevibacillus	WP_171565830	--E--T---S--T-V-TV	Q	RHPG-S----------
Other Bacteria (2/ >40)	*Anoxybacillus flavithermus*	WP_004888851	--E-KTIVA--DTVVDVV		KKHP-S--S--I--E-
	Calditerricola satsumensis	WP_188817311	--E-ET------T-V--V		RAAGLKP---TV----
	Cytobacillus firmus	WP_274576672	TAK-RTI----ET-A-DI		-VHG-S--SM------
	Bacillus methanolicus	WP_150916624	TEK-RTI-----T-A-DI		RKHD-S--SLV-----
	Peptococcus niger	WP_091790740	R-E--T-----ET-V-VV		EAAGF-S----II---
	Bacillus pakistanensis	MBM7587617	TKK-RTI--N-ST-AQDI		-EHR-S---M------
	Cytobacillus oceanisediminis	WP_217031655	TAN-RTI----ET-A-DI		-IHG-S--SM------
	Tumebacillus flagellatus	WP_038091811	M-E-RT-V---ST-VDIA		REQE-A--S-------
	Sporosarcina globispora	WP_053435458	TAN-RTI----ET-AKDI		-YHG-S--SM------
	Alkalihalobacillus akibai	WP_052013286	YSK-KT---S--T-ANKV		-QEN----------N-
	Bacillus solitudinis	WP_100407083	M-E-R-------T-V-VS		AEQK-K--S--I----
	Robertmurraya siralis	WP_095311078	TEK-RTI-----T-A-DI		RKHD----SLV-----
	Spirosoma profusum	WP_190886126	--E-RS-F-RVDN-V--V		NQQG-D-----V----
	Aliifodinibius salicampi	WP_265791227	--EEKIAL--VET-VHEV		DKHGL----L--I---
	Massilibacterium senegalense	WP_062198065	MGS-QT-----ET-VQCV		EE-N-S----V---N-
	Caldanaerobius fijiensis	WP_073342842	M-D-VT-I---K--SQKV		KE-G-S---V-VI---
	Desulfuribacillus stibiiarsenatis	WP_069703436	R-E-WTL----ET-V-TV		EKAKF-S-------E-
	Tumebacillus permanentifrigoris	WP_245884401	M-E-RT-V---TT-V-IA		REQE-A--S-----E-
	Shouchella gibsonii	WP_203086533	-GK-RT-----ST-VQKI		KEEKVS----T-----
	Tumebacillus algifaecis	ASS74197	--E-NT-V---ET-VDIA		REQE-A--S-------
	Robertmurraya massiliosenegale	WP_019156691	--E-KT-----DT-C-MV		KKEN-S--SM-VI-E-
	Aneurinibacillus tyrosinisolvens	WP_236692649	MGR-KTL-----S-V-EV		DK-GFS--------EI
	Spirosoma validum	WP_191040045	--E-QTAF-RVDT-VDQV		AQQG-D-----II---
	Neobacillus niacini	WP_045522281	TKN-RTI--N-ST-AQDI		-IHR-S---M------
	Caldanaerobius polysaccharolyticus	WP_026486922	T-H-TT-V---K--CRKV		KE-G-G---V-VI---
	Mesobacillus persicus	WP_090740576	TKE-RT------T-VGIV		KHED-Q---M-V--E-
	Oscillospiraceae bacterium	MCL2163588	TAE-RCIS-V--T-V-NV	R	EKGGART---VI--K-
	Candidatus Dadabacteria bacterium	MCC6713226	Y-S--S----V---A-KA	-	ARED-SS---VVT-E-

Figure S27: Partial sequence alignment of the uroporphyrinogen-III C-methyltransferase protein showing a 1 aa insert (highlighted) in a conserved region that is generally exclusively shared by *A.sediminis* and species from the genus *Brevibacillus*. This CSI is also not shared by most other species from the family *Anoxybacillaceae* or other bacteria. A few exceptions in distantly related uncharacterized species are present.

Group	Species	Accession	84		125
Brevibacillus (20/ 20)	*Brevibacillus brevis*	WP_012684838	EVAERICRFPEVKAVYLMSGA	S	YDLSIILEGKTMKEVATFVS
	Brevibacillus invocatus	WP_122910587	---------------------	-	----VV------R---S---
	Brevibacillus choshinensis	WP_055747346	---------------------	-	----VV------R-------
	Brevibacillus reuszeri	WP_049738126	---------------------	-	-----V------R---N---
	Brevibacillus panacihumi	WP_031305559	---K--------Q--------	-	----VV------R---S---
	Brevibacillus fortis	WP_106841352	---------------------	-	--------------------
	Brevibacillus formosus	WP_197241511	---------------------	-	--------------------
	Brevibacillus parabrevis	WP_063229677	---------------------	-	--------------------
	Brevibacillus borstelensis	WP_003392188	---D--------Q--------	-	----VV------R---A---
	Brevibacillus gelatini	WP_122906909	---Q-----------------	-	--------------------
	Brevibacillus composti	WP_198827330	---D--------Q-------V	-	----VV------R-------
	Brevibacillus ruminantium	WP_251871792	---D--------E--------	-	----VV------R---A---
	Brevibacillus humidisoli	WP_230043606	---------------------	G	----VV------R--SS---
	Brevibacillus daliensis	WP_232697879	K-----A--------------	G	--I-VL------H-I-N--T
	Brevibacillus marinus	WP_126428830	------G--------------	G	----VV------R---S---
	Brevibacillus aydinogluensis	WP_212133200	---------------------	-	----VV------R---S---
	Brevibacillus centrosporus	WP_092266437	---------------------	-	----VV------R-------
	Brevibacillus fluminis	WP_122920626	------S-----E--------	N	----VVM---S-R---D---
	Brevibacillus laterosporus	WP_239208477	K-----S--------------	G	--I-VV------H---N--T
	Brevibacillus dissolubilis	WP_139491106	---K--T--------------	-	----VV-----LH---N---
A.sediminis (1/ 1)	*Anxoybacillus sediminis*	WP_171566593	---------------------	-	----VV------R---S---
Unnamed *Brevibacillus* strains	*Brevibacillus sp.*	MBO8165416	---------------------	G	----VV------R---S---
	Brevibacillus sp. M2.1A	WP_173609076	---------------------	-	--------------------
	Brevibacillus sp. AF8	WP_232773581	---------------------	-	--------------------
	Brevibacillus sp. HB2.2	WP_173606450	---------------------	-	--------------------
	unclassified Brevibacillus	WP_173623232	---------------------	-	--------------------
Other Bacteria (1/ >100)	*Anoxybacillus ayderensis*	MCL6618032	------Y------S-------		----VVI--RS-S-I-H---
	Anoxybacillus pushchinoensis	WP_091700898	------Y------S-------		----VVI--RS-S-I-H---
	Anoxybacillus rupiensis	MBB3909209	------Y------S-------		----VVI--RS-S---H---
	Anoxybacillus calidus	WP_181537335	------YK-----S-------		----VVI--RS-S---H---
	Anoxybacillus flavithermus	WP_003398747	------Y------S-------		----VVI--RS-S-I-H---
	Anoxybacillus caldiproteolyticus	WP_181556393	------Y------S-------		----V-I--RS-S---Q---
	Anoxybacillus voinovskiensis	WP_183185903	------Y------S-------		----VVI--RS-A---H---
	Anoxybacillus amylolyticus	WP_066322900	------Y------S-------		----VVI--RS-A---H---
	Geobacillus kaustophilus	WP_011232400	------Y------S-------		----VVI--RS-A-I-Q---
	Geobacillus proteiniphilus	OKO88981	------Y------S-------		----VVI--RS-A-I-Q---
	Geobacillus thermoleovorans	AEV20583	------Y------S-------		----VVI--RS-A-I-Q---
	Geobacillus jurassicus	WP_066227916	------Y------S-------		----VVI--RS-A-I-Q---
	Parageobacillus toebii	WP_062755864	------Y------S-------		----VVI--RS-S---Q---
	Parageobacillus thermantarcticus	WP_090951841	------YH-----S-------		----VVI--RS-S---K---
	Saccharococcus thermophilus	WP_166908109	------Y------S-------		----VVI--RS-S---Q---
	Thermolongibacillus altinsuensis	WP_132949005	------Y------S-------		----VVI--RS-S-I-H---
	Bacillus taeanensis	WP_113804046	------Y--------------		----VQI-----T---S---
	Aneurinibacillus migulanus	KIV52366	D-----Y-----QS-------		----VVI-----RQ--Q---
	Halalkalibacterium halodurans	WP_010899492	------Y-------L------		----VVI-----S---R---
	Aneurinibacillus terranovensis	WP_035099958	D-----Y-----QS-------		----VVI-----RQ--Q---
	Alkalihalobacillus okhensis	WP_034626549	------Y-------L------		----VVI-----S-I-R---
	Aneurinibacillus danicus	WP_174760626	DI----Y-----QS-------		----VVI-----RQ--Q---
	Ectobacillus funiculus	WP_129729368	-I--K-Y-Y----S-------		-----V------S---S--A
	Alkalihalophilus marmarensis	WP_251189523	Q-----Y-------L------		----VVI-----S-I-R---
	Salsuginibacillus kocurii	WP_018921816	------Y-----R-L------		----VVI-----A-I-R---
	Anaerobacillus alkaliphilus	WP_129077440	D-----Y-----R-L------		----VVI-----N-I-R---
	Marinococcus halophilus	WP_079476998	------H-------L--V--T		---QVVI-----S-I-S---
	Sediminibacillus dalangtanensi	WP_209366493	-----Y--------------		----VQI---------F---
	Virgibacillus senegalensis	WP_053217541	-----Y--------------		----VQI---------F---
	Calderihabitans maritimus	WP_088553320	------Y------S------G		----VVI----L----R--A
	Neobacillus kokaensis	WP_191274383	------A-----SSL------		-----TI-----NQI-S---
	Bacillus alveayuensis	WP_044747385	------Y------S-------		----V-I--RS-S---H---
	Alteribacillus bidgolensis	SDH70566	------Y-------L--V---		----VVID-N--S-I-S---
	Neobacillus dielmonensis	WP_042462686	------A-----SSL------		-----TI-----Q-I-A---
	Terribacillus saccharophilus	WP_095220014	D-----Y--------------		----VSV-----SQ--R---
	Salibacterium halotolerans	WP_093336301	------Y-------L--V---		----VVID----S-I-G---
	Salsuginibacillus halophilus	WP_106588953	------Y-----Q-L------		---AVVI--E--S-I-A---
	Bacillus salipaludis	WP_133333856	------A-----SSL------		-----TI-----NQI-N---
	Rhodococcus rhodochrous	WP_016696531	---D--------Q--------	-	----VV------R---A---

Figure S28: Partial sequence alignment of the Lrp/AsnC family transcriptional regulator protein showing a 1 aa insert (highlighted) in a conserved region that is generally exclusively shared by *A.sediminis* and species from the genus *Brevibacillus*. This CSI is also not shared by most other species from the family *Anoxybacillaceae* or other bacteria. A few exceptions in a distantly related species are present.

Group	Species	Accession	27	Insert	65
Geobacillus (12/ 12)	*Geobacillus stearothermophilus*	OAO76761	GQVGPRVAELLGIPYVTTITKLDI	A	DGGNVTVVRDVEGD
	Geobacillus thermoleovorans	AMV11875	------------------------	-	---K----------
	Geobacillus vulcani	WP_031407824	------------------------	-	---K----------
	Geobacillus kaustophilus	WP_044733052	------------------------	-	---K----------
	Geobacillus proteiniphilus	OKO97197	------------------------	-	---K----------
	Geobacillus jurassicus	WP_066226750	------------------------	V	---K----------
	Geobacillus thermodenitrificans	WP_008881143	------------------------	-	---K----------
	Geobacillus subterraneus	WP_063165073	------------------------	-	---K----------
	Geobacillus uzenensis	WP_063165073	------------------------	-	---K----------
	Geobacillus zalihae	WP_013144555	------------------------	-	---K----------
	Geobacillus thermocatenulatus	WP_025949432	------------------------	-	---K----------
	Geobacillus icigianus	WP_033020346	----------------------E-	-	--------------
Unnamed *Geobacillus* strains	*Geobacillus genomosp. 3*	WP_020960813	------------------------	-	---K----------
	Geobacillus sp. TFV-3	WP_160155630	------------------------	-	---K----------
	Geobacillus sp. A8	EQB95661	------------------------	-	---K----------
	Geobacillus sp. BMUD	WP_171660488	------------------------	-	---K----------
	Geobacillus	WP_044742492	------------------------	-	---K----------
	Geobacillus sp. PK12	WP_129447349	------------------------	-	---K----------
	unclassified Geobacillus	WP_100658973	------------------------	-	---K----------
	Geobacillus sp. BCO2	KPD01502	------------------------	-	--------------
	Geobacillus sp. DSP4a	WP_171698047	------------------------	-	--------------
	Geobacillus sp. WSUCF1	EPR27862	------------------------	-	---K----------
	Geobacillus sp. 46C-IIa	WP_081208616	------------------------	-	--KK----------
Other *Anoxybacillaceae* (0/ >15)	*Parageobacillus toebii*	WP_062677857	------------------------		--DK--I-------
	Parageobacillus thermoglucosidasius	WP_064549615	------------------------		--DK--I-------
	Parageobacillus thermantarcticus	WP_090947580	------------------------		--DK----------
	Anoxybacillus pushchinoensis	WP_091701397	----------------------E-		--T--K--------
	Anoxybacillus thermarum	WP_043965883	----------------------E-		--T--K--------
	Anoxybacillus tepidamans	WP_183253530	---------M--------------		--DK----------
	Anoxybacillus caldiproteolyticus	WP_194519043	-------------S----------		---K--I-------
	Anoxybacillus voinovskiensis	WP_183183533	------------------------		--DKA---------
	Anoxybacillus vitaminiphilus	WP_111644565	------------------------		---K-M--------
	Anoxybacillus amylolyticus	WP_066326616	------------------------		--DKA-I-------
	Anoxybacillus flavithermus	WP_003396511	----------------------E-		--T--K--------
	Anoxybacillus calidus	WP_181537508	------------------------		--SK----------
	Anoxybacillus suryakundensis	WP_055440040	----------------------E-		--T--KA-------
	Anoxybacillus tengchongensis	WP_183249733	----------------------EM		--T--KA-------
	Anoxybacillus mongoliensis	WP_183241349	----------------------E-		--T--KA-------
	Saccharococcus thermophilus	WP_166910621	------------------------		--NK----------
	Thermolongibacillus altinsuensis	WP_132947574	----------------------E-		--T--K--------
Other Bacteria (0/ >50)	*Bacillus alveayuensis*	WP_044894560	------------------------		---K--I-------
	Sutcliffiella cohnii	WP_066415440	-----------N----------E-		---K--I-------
	Bacillus kwashiorkori	WP_062355311	------------------------		N-T--S--------
	Neobacillus fumarioli	WP_066365924	-----------N------------		--TK---T------
	Sutcliffiella horikoshii	WP_064100183	-----------D----------E-		---KA-I-------
	Neobacillus mesonae	WP_251468896	----------------------E-		--NQ---T------
	Weizmannia coagulans	WP_133536945	--------------------N-E-		---KA--T------
	Neobacillus massiliamazoniensis	WP_090637947	-----------N----------E-		--NK---T------
	Cytobacillus horneckiae	WP_066191537	-----------N---I--------		--T-----------
	Peribacillus butanolivorans	WP_053347222	---------I-----I--------		AA---S-I------
	Bacillus sinesaloumensis	WP_077619011	-----------D----------EV		--SK----------
	Cytobacillus eiseniae	WP_066396545	-----------N----------E-		--S-----------
	Cytobacillus solani	WP_053477228	-----------N------------		A-S-A---------
	Heyndrickxia vini	WP_202779777	--------D---------------		N-E-----------
	Bacillus dafuensis	WP_057771876	-----------N----------E-		A-S-----------
	Weizmannia acidiproducens	WP_018664919	--------D-------------E-		--EK---T------
	Bacillus tepidiphilus	WP_153122931	---------------I----N-E-		--TK-S--------
	Peribacillus simplex	WP_137024178	---------I--------------		EN---S-I------
	Peribacillus muralis	WP_064465930	---------I-E---I--------		AD---S-I------
	Peribacillus faecalis	WP_190997922	-----------N---I--------		--T-A---------
	Caldibacillus thermolactis	WP_173661955	--------D-----------D-K-		--EK----------
	Bacillus canaveralius	WP_101578190	-------------S--------S-		---T----------
	Bacillus rubiinfantis	WP_042354853	---------------I------E-		--ST---T------
	Caldibacillus kokeshiiformis	WP_263114798	--------D-----------D-K-		--EK----------
	Peribacillus frigoritolerans	WP_262869522	---------I-----I--------		EN---S-I------
	Robertmurraya siralis	WP_095307734	-----------Q----------E-		--SE--I-------
	Neobacillus piezotolerans	WP_115452743	----------------------E-		---TAKI---I---
	Fredinandcohnia onubensis	WP_099352055	---------I-D------------		--ST----------
	Bacillus massilionigeriensis	WP_075982506	-----------N----------E-		--SKA--T------
	Bacillus pakistanensis	WP_205169419	---------Q-D---I--------		--DK----------

Figure S29: Partial sequence alignment of the electron transfer flavoprotein beta subunit showing a 1 aa insert (highlighted) in a conserved region that is exclusively shared by species from the genus *Geobacillus*. This CSI is also not shared by any other species from the family *Anoxybacillaceae* or other bacteria.

Group	Species	Accession	295		328
Geobacillus (12/ 12)	*Geobacillus stearothermophilus*	KAF6510349	INEVAARVPHLAKLAPASDIH	Y	IEDLHEAGGVSA
	Geobacillus thermodenitrificans	AB067286	-----S-------------V-	-	------------
	Geobacillus kaustophilus	WP_011231532	-----S-------------V-	-	------------
	Geobacillus jurassicus	WP_066228893	---I-S-------------V-	F	------------
	Geobacillus zalihae	WP_047818498	---I---------------V-	-	------------
	Geobacillus thermoleovorans	QDY73631	---I---------------V-	-	------------
	Geobacillus vulcani	WP_031408961	---I-----------------	-	------------
	Geobacillus subterraneus	WP_063165687	-------------------V-	-	------------
	Geobacillus uzenensis	WP_063165687	-------------------V-	-	------------
	Geobacillus thermocatenulatus	WP_025950504	-----S---------------	-	------------
	Geobacillus proteiniphilus	WP_013145048	---I---------------V-	-	------------
	Geobacillus icigianus	WP_033019062	---I-----------------	-	------------
Unnamed *Geobacillus* strains	*Geobacillus sp. WSUCF-018B*	WP_100664310	---------------------	-	------------
	Geobacillus sp. GHH01	AGE22607	---I-----------------	-	------------
	Geobacillus sp. 46C-IIa	WP_081210988	-----S-------------V-	-	------------
	Geobacillus sp. C56-T2	WP_144974848	-----S---------------	-	------------
	Geobacillus sp. BC02	KPD00427	-----S---------------	-	------------
	Geobacillus genomosp. 3	WP_020960162	-------------------V-	-	------------
	Geobacillus sp. ZGt-1	WP_236687333	-----S-------------V-	-	------------
	Geobacillus	WP_063165687	-------------------V-	-	------------
	Geobacillus sp. WSUCF1	EPR27356	---------------------	-	------------
	Geobacillus sp. 12AMOR1	AKM19227	---I---------------V-	-	------------
	Geobacillus sp. BMUD	WP_171660600	---I-----------------	-	------------
	Geobacillus sp. TFV-3	WP_160156433	---I---------------V-	F	------------
Other *Anoxybacillaceae* (0/ >15)	*Parageobacillus toebii*	WP_062678014	---I---------------V-		------------
	Parageobacillus thermantarcticus	WP_090949962	---I--K------------V-		------------
	Parageobacillus thermoglucosidasius	WP_013400568	---I---------------V-		------------
	Parageobacillus caldoxylosilyticus	WP_017436004	---I---------------V-		------------
	Anoxybacillus voinovskiensis	WP_183184949	-----S-------------M-		------------
	Anoxybacillus flavithermus	WP_041638303	-----S-------------M-		------------
	Anoxybacillus caldiproteolyticus	WP_258561043	-------------------V-		------------
	Anoxybacillus kestanbolensis	WP_077428508	-----S-------------M-		------------
	Anoxybacillus mongoliensis	WP_183242238	-----S-------------M-		------------
	Anoxybacillus amylolyticus	WP_066324265	-----S-------------M-		------------
	Anoxybacillus pushchinoensis	WP_091700724	-----S-------------M-		------------
	Anoxybacillus tepidamans	WP_027409049	-----S---------------		------------
	Anoxybacillus rupiensis	WP_183186959	---------------------		------------
	Anoxybacillus gonensis	EMI11218	-------------------M-		------------
	Anoxybacillus tengchongensis	WP_183248208	-------------------M-		------------
	Anoxybacillus ayderensis	WP_085787944	-------------------M-		------------
	Anoxybacillus suryakundensis	CUA80703	-------------------M-		------------
	Anoxybacillus thermarum	KIQ94040	-------------------M-		------------
	Saccharococcus thermophilus	WP_166909549	---I---------------V-		------------
Other Bacteria (1/ >30)	*Paenibacillus azotifigens*	WP_193567392	----------S--------VF		------------
	Bacillus aquiflavi	WP_163242035	-----S-------------V-		------------
	Bacillus alveayuensis	WP_044896240	-------------------V-		------------
	Bacillus tequilensis	WP_167872727	-----E-------------VY		------------
	Niallia nealsonii	WP_101178510	-----E-------------V-		------------
	Priestia megaterium	TYR80543	-----S-----S-------VF		------------
	Niallia circulans	WP_061800075	-----S-----S----S--V-		------------
	Niallia taxi	WP_127740735	-----S-----S----S--V-		------------
	Priestia endophytica	WP_268584665	-----------S-------V-		------------
	Priestia filamentosa	WP_243519590	-----------S-------V-		------------
	Priestia abyssalis	WP_078410423	-----------S-------V-		------------
	Bacillus kexueae	WP_243387863	-----N-----S-------V-		------------
	Bacillus massiliglaciei	WP_110927620	-----------S-------V-		------------
	Bacillus atrophaeus	WP_061573033	-----E-----S-------VY		------------
	Bacillus vallismortis	WP_268551355	-----E-----S-------VY		------------
	Bacillus velezensis	WP_139886910	-----E-----S-------VY		------------
	Bacillus inaquosorum	WP_268438934	-----E-------------VF		------------
	Bacillus nakamurai	WP_061521027	-----E-------------VF		------------
	Bacillus spizizenii	WP_242733872	-----E-------------VF		------------
	Bacillus halotolerans	WP_106293841	-----E-------------VF		------------
	Streptococcus pneumoniae	CJS15957	-----E-------------VF		------------
	Bacillus intestinalis	OPG92445	-----E-------------VF		------------
	Bacillus mojavensis	WP_268471366	-----E-------------VF		------------
	Bacillus cabrialesii	WP_215797401	-----E-------------VF		------------
	Priestia aryabhattai	WP_045294483	-----E-------------VF		------------
	Flavobacterium thermophilum	ST012398	---I---------------V-	-	------------

Figure S30: Partial sequence alignment of the dihydroxy-acid dehydratase showing a 1 aa insert (highlighted) in a conserved region that is generally exclusively shared by species from the genus *Geobacillus*. This CSI is also not shared by most other species from the family *Anoxybacillaceae* or other bacteria. One exception in a distantly related species is present.

Group	Species	Accession	84	Insert	117
Geobacillus (12/ 12)	*Geobacillus stearothermophilus*	WP_095858690	FLVNGSTAGNLAMIAAVCDKNK	Q	KVIVQRNCHKS
	Geobacillus thermoleovorans	WP_068895949	-------------------GKR	K	-----------
	Geobacillus kaustophilus	WP_011229539	-------------------GKR	K	-----------
	Geobacillus vulcani	WP_031406361	-------------------GKR	K	-----------
	Geobacillus zalihae	WP_060788333	-------------------GKR	K	-----------
	Geobacillus jurassicus	WP_066231077	-------------------GKR	K	-----------
	Geobacillus proteiniphilus	OK093512	-------------------GKR	K	-----------
	Geobacillus thermodenitrificans	WP_011886599	-------S-----------EK-	R	-----------
	Geobacillus subterraneus	WP_033842748	------------------REKG	-	-----------
	Geobacillus uzenensis	WP_063167066	-------------------EK-	R	-----------
	Geobacillus thermocatenulatus	WP_025950895	-------------------GKR	K	-----------
	Geobacillus icigianus	WP_033017934	------------------REKG	-	-----------
Unnamed *Geobacillus* strains	*Geobacillus*	WP_025039043	-------------------GKR	K	-----------
	Geobacillus sp. A8	EQB94407	-------------------GKR	K	-----------
	Geobacillus sp. C56-T3	WP_013143956	-------------------GKR	K	-----------
	Geobacillus sp. Y412MC52	WP_012820430	-------------------GKR	K	-----------
	Geobacillus sp. BMUD	WP_171661373	-------------------GKR	K	-----------
	Geobacillus sp. JS12	WP_063193890	-------------------GKR	K	-----------
	Geobacillus sp. TFV-3	WP_160155092	-------------------GKR	K	-----------
	Geobacillus sp. MAS1	ESU70882	-------------------GKR	K	-----------
	Geobacillus sp. WSUCF1	EPR26288	-------------------GKR	K	-----------
	Geobacillus sp. BCO2	KPC99495	-------------------GKR	K	-----------
	Geobacillus genomosp. 3	WP_023817403	-------------------EK-	R	-I---------
	Geobacillus sp. PA-3	WP_060475481	-------S-----------EK-	R	-----------
	Geobacillus sp. MR	WP_171355519	-------S-----------EK-	R	-----------
	Geobacillus sp. 46C-IIa	WP_081210890	---------------T---EK-	R	-----------
	Geobacillus sp.	MBR2516119	----------------------	-	-----------
	Geobacillus sp. Sah69	WP_055358753	----------------------	-	-----------
	Geobacillus sp. C56-T2	WP_144976939	------------------REKG	-	-----------
	Geobacillus sp. B4113_201601	KYD27297	------------------REKG	-	-----------
	unclassified Geobacillus	WP_033006942	-------------------GKR	K	-----------
Other *Anoxybacillacae* (0/ >15)	*Parageobacillus toebii*	WP_062756509	--------------F---GEK-		-----------
	Parageobacillus caldoxylosilyticus	WP_042410427	-------T------F---GEK-		-----------
	Parageobacillus thermoglucosidasius	WP_256833679	-------V------F---GEK-		-----------
	Parageobacillus thermantarcticus	WP_090949745	-------V------F-A-GEK-		-----------
	Anoxybacillus flavithermus	WP_099669045	----------------A-E---		-----------
	Anoxybacillus tengchongensis	WP_183250113	----------------A-E---		-----------
	Anoxybacillus suryakundensis	WP_055441886	----------------A-E---		-----------
	Anoxybacillus mongoliensis	WP_183244436	---------------V--E---		-----------
	Anoxybacillus pushchinoensis	WP_091704201	----------------A-VQ--		-----------
	Anoxybacillus thermarum	WP_043968423	--------------T-A-E---		-----------
	Anoxybacillus kestanbolensis	WP_077428159	--------------V-A-E---		-----------
	Anoxybacillus ayderensis	WP_085788852	-------S------T-A-E---		-----------
	Anoxybacillus calidus	WP_181538822	-------S------F---EE--		-----------
	Anoxybacillus caldiproteolyticus	WP_199426855	-------V------F-T-EEE-		-----------
	Anoxybacillus amylolyticus	WP_066322245	-------V------F-T-EEH-		-----------
	Anoxybacillus voinovskiensis	WP_183185568	-------V------F-T-EEH-		-----------
	Anoxybacillus vitaminiphilus	WP_111646251	-------S---T--F---EE--		-I---------
	Anoxybacillus rupiensis	WP_240371913	--I----V------F-T-EE-E		P----------
	Anoxybacillus tepidamans	MBB5325868	-------V-----VF-T-EE--		E----------
	Saccharococcus thermophilus	WP_166907420	--------------F-A-GEK-		-----------
	Thermolongibacillus altinsuensis	WP_132949369	-------V-------T--QE--		-I---------
Other Bacteria (0/ >50)	*Priestia taiwanensis*	WP_188390040	-------V------LS--GE-D		--L--------
	Bacillus alveayuensis	WP_044895816	-------S---V--FS--EE-R		-----------
	Filobacillus milosensis	WP_134340201	--------------LS--GPGD		SI---------
	Halobacillus fulvus	RWZ50141	--I----V-----VL---GRGD		TI---------
	Sutcliffiella deserti	WP_223703280	-------V------L-T-TRKS		R-L--------
	Bacillus pinisoli	WP_246945982	-------V-----VLS--SRDE		--L--------
	Halobacillus litoralis	QAS54621	-------------VLS--KPGE		-I-I-------
	Planococcus ruber	WP_246808589	-------V------Y---G-GD		Q------S---
	Paraliobacillus salinarum	WP_182201638	--I----V------L-S-GMDD		---I-------
	Oceanobacillus indicireducens	WP_188856991	--I----V------L-T-KQGE		-----------
	Thalassobacillus pellis	WP_205094575	--I----S-----LMS--MED-		--L--------
	Gracilibacillus thailandensis	WP_153836827	-------V------L-T--SGD		Q-L--------
	Gracilibacillus saliphilus	WP_163582981	-------V------L-T--SGD		Q-L--------
	Gracilibacillus lacisalsi	WP_018934309	-------V------L-T--SGD		Q-L--------
	Amphibacillus marinus	WP_091497702	-------V------L-T-GL-D		QI---------
	Bacillus massiliglaciei	WP_110926060	----------I---L-A-GEGD		T-L--------
	Planococcus rifietoensis	WP_058382248	--------------H---GEGD		S------S---
	Priestia megaterium	WP_168241443	-------V------M-A-SDGT		--L--------

Figure S31: Partial sequence alignment of the aminotransferase class I/II-fold pyridoxal phosphate-dependent enzyme protein showing a 1 aa insert (highlighted) in a conserved region that is exclusively shared by species from the genus *Geobacillus*. This CSI is also not shared by any other species from the family *Anoxybacillaceae* or other bacteria.

Group	Species	Accession	124		164
Geobacillus (12/ 12)	*Geobacillus stearothermophilus*	WP_033016698	RSIKAAEKLPYVTPANETRRALLEQI		GTDVYNLYEITKDNY
	Geobacillus thermocatenulatus	WP_025949107	--------------------------		---------------
	Geobacillus thermoleovorans	UPT58425	--------------------------		-A-I-----------
	Geobacillus thermodenitrificans	WP_029761747	-----V-------S------T-----		-A-------------
	Geobacillus icigianus	WP_033018873	-----------I--------P-----		-A---D---------
	Geobacillus kaustophilus	WP_044732316	--------------------------		-A-------------
	Geobacillus jurassicus	WP_066230889	--------------------------		-A-------------
	Geobacillus subterraneus	WP_063166746	------------A-------------		-A-------------
	Geobacillus uzenensis	WP_063166746	------------A-------------		-A-------------
	Geobacillus zalihae	WP_011229954	--------------------------		-A-------------
	Geobacillus proteiniphilus	WP_011229954	--------------------------		-A-------------
	Geobacillus vulcani	WP_031405643	-------------------QS-----		-A-------------
Parageobacillus (6/ 6)	*Parageobacillus thermoglucosidasius*	WP_013401620	-------------------KS-----		-A-I-----------
	Parageobacillus caldoxylosilyticus	WP_017436573	-------------------KS-----		-A-I-----------
	Parageobacillus yumthangensis	PDM39820.1	-------------------KS-----		-A-------------
	Parageobacillus thermantarcticus	WP_090950030	-------------------KS-----		-A-------------
	Parageobacillus galactosidasius	WP_012749178	-------------------KS-----		-A-------------
	Parageobacillus toebii	WP_012749178	-------------------KS-----		-A-------------
Saccharococcus (1/ 1)	*Saccharococcus thermophilus*	WP_166911912	-------------------KN-----		-A-------------
Unnamed *Geobacillus* and *Parageobacillus* strains	*Geobacillus*	WP_100664095	------------M-------------		-A-------------
	Geobacillus sp. DSP4a	WP_050368226	--------------------------		-A-------------
	Geobacillus sp. 12AMOR1	AKM17812	--------------------------		-A-------------
	Geobacillus sp. Manikaran-105	WP_100659743	--------------------------		-A-------------
	Geobacillus sp. WSUCF1	EPR28724	--------------------------		-A-------------
	Geobacillus sp. 46C-IIa	WP_081210834	------------M-------------		-A-------------
	Geobacillus sp. C56-T3	WP_013146322	--------------------------		-S-------------
	Geobacillus sp. T6	KLR75232	--------------------------		---------------
	Geobacillus sp. JS12	WP_063193748	--------------------T-----		-A-------------
	Geobacillus sp. TFV-3	WP_160155417	---------------S----------		-A-------------
	Geobacillus sp. C56-T2	WP_144972813	--------------------P-----		-A---D---------
	Geobacillus genomosp. 3	WP_020958676	--------------------------		-A-------------
	Parageobacillus	WP_012749178	-------------------KS-----		-A-------------
	Parageobacillus sp. VR-IP	WP_175245506	-------------S-----KS-----		-A-I-----------
	Parageobacillus genomosp. 1	WP_043903564	-------------------KS-----		-A-------------
Other *Anoxybacillaceae* (0/ >10)	*Anoxybacillus amylolyticus*	WP_066327128	--T----------L-----K--F---	NQ	-E-I------S----
	Anoxybacillus voinovskiensis	WP_183183835	--T----------L-----K--F---	NQ	-E-I------S----
	Anoxybacillus tepidamans	WP_027410701	-------------L-----KS-----	NQ	-Q-I-H----S----
	Anoxybacillus caldiproteolyticus	WP_181557194	-------------L-----KS-----	NQ	-E-I------S----
	Anoxybacillus calidus	WP_220129239	-------------F-----KS-----	NN	-Q-I------S----
	Anoxybacillus vitaminiphilus	WP_111646347	--T----------L-----KH-----	NN	-E-I------S----
	Anoxybacillus pushchinoensis	WP_091705034	--T----------L-----KR-W-E-	NQ	-E-I------S----
	Anoxybacillus flavithermus	WP_004888898	--T----------L-----KR-W-E-	NQ	-E-I------S----
	Anoxybacillus kestanbolensis	WP_077429965	--T----------L-----KR-W-E-	NQ	-E-I------S----
	Anoxybacillus ayderensis	WP_021095750	--T----------L-----KP-W-AM	NQ	-E-I------S----
	Anoxybacillus gonensis	WP_009360846	--T----------L-----KQ-W-A-	NQ	-E-I------S----
	Thermolongibacillus altinsuensis	WP_132949183	-------------F-----KS--DE-	NQ	-E-I------S----
Other Bacteria (0/ >50)	*Neobacillus thermocopriae*	QAV27641	--T----------L-----KR-W-E-	NQ	-E-I------S----
	Bacillus methanolicus	WP_274854696	--Q---------I------KS-F--L	NA	AGNI---F--S--Q-
	Sutcliffiella halmapala	WP_078381765	--M---------VL-----KS--DE-	NG	-E-I------S--Q-
	Metabacillus mangrovi	WP_155113978	-----------AIN-----T------	NN	-E-IFS-------L-
	Sutcliffiella deserti	WP_223703436	--M---------IL-----QS---E-	NG	-E-I------SR-Q-
	Metabacillus schmidteae	WP_102233056	--T---------MN-----AH-----	NN	-E-IF--F---R-Q-
	Bacillus tianshenii	WP_204414762	--T---------VL-----QS--DE-	NG	-E-I------S--L-
	Neobacillus niacini	WP_221823573	------------VK-----KS-Y--L	NA	DG---D----P--Q-
	Metabacillus flavus	WP_211556601	--T---------EC-----K---D--	NN	-E-I-S----K--L-
	Sutcliffiella horikoshii	WP_223492476	--T-----I---VL-----KS--DE-	NG	-E-I------SR-Q-
	Metabacillus halosaccharovorans	WP_264142724	--TR--------VN-----AH-----	NN	-E-IF--F---R-Q-
	Metabacillus litoralis	WP_226671235	--T---------VN-----AN-----	NN	-E-LF--F-----L-
	Peribacillus frigoritolerans	TDL77779	--T---------VN-----KP--DE-	NQ	-E-I------S--L-
	Metabacillus endolithicus	WP_247339578	--T---------VN-----AH-----	NN	-E-IF--F--SR-Q-
	Metabacillus idriensis	WP_191556639	--T---------VN-----KP--DE-	NQ	-E-I------S--L-
	Metabacillus dongyingensis	WP_223439712	--T---------VN-----KP--DE-	NQ	-E-I------S--L-
	Mesobacillus subterraneus	WP_251480536	------------VK-----Q-IY--L	NA	NE-I-D----P--Q-
	Metabacillus kandeliae	WP_231791021	--T---------INS----A------	NE	-Q-I---F--P--L-
	Litchfieldia alkalitelluris	WP_078549868	--TM--------AL-----KS--DE-	NG	-Q-I-----VS--L-
	Bacillus weihaiensis	WP_273129248	--T---A-----VN-----AS-----	NN	-E-IF--F-----L-
	Metabacillus indicus	WP_029285924	--T---------VY-----KP--DE-	NQ	-E-I------S--L-
	Sutcliffiella rhizosphaerae	WP_230504028	--T---------VF-----KS--DE-	ND	-E-I------SR-Q-
	Metabacillus bambusae	WP_207982426	--T---A------N-----AS-----	NN	-E-LF--F-----L-
	Metabacillus crassostreae	WP_204954836	--T---------VN-----AN-----	NN	-E-LF--F--S--L-

Figure S32: Partial sequence alignment of the GNAT family protein showing a 2 aa deletion (highlighted) in a conserved region that is exclusively shared by species from the larger *Geobacillus* clade. This CSI is also not shared by any other species from the family *Anoxybacillaceae* or other bacteria.

Group	Species	Accession	Sequence (6–55)
			6 … 55
Geobacillus (11/ 11)	*Geobacillus stearothermophilus*	WP_064213561	VDGYNIIGAWPALRRLKEE G DLAAARDLLIDKLADYKGFTGDHVVVVFDA
	Geobacillus thermocatenulatus	WP_025950555	-----------E------- - ----------E-M------------I----
	Geobacillus kaustophilus	WP_044732996	-----------E------- - ----------E-M------------I----
	Geobacillus zalihae	WP_081212669	-----X-----E------- - ----------E-M------------I----
	Geobacillus jurassicus	WP_066234029	-----------E-----D- - ----------E-M------------I----
	Geobacillus thermodenitrificans	WP_029761773	-----------V--Q---- - ------------M-----------MI----
	Geobacillus subterraneus	WP_063167007	------------------- - ------------M-----------II----
	Geobacillus uzenensis	WP_063167007	------------------- - ------------M-----------II----
	Geobacillus thermoleovorans	WP_012820478	-----------E------- - ----------E-M------------I----
	Geobacillus proteiniphilus	WP_012820478	-----------E------- - ----------E-M------------I----
	Geobacillus icigianus	WP_033021043	-----M------------- - ----------A--------------I----
Parageobacillus (6/ 6)	*Parageobacillus thermoglucosidasius*	WP_003247524	-----M-----E--K---- D ----------S-M-E-Q-----K-II----
	Parageobacillus yumthangensis	PDM39382.1	-----M-----E--K---- D ----------S-M-E-Q-----Q-II----
	Parageobacillus thermantarcticus	WP_090949434	-----M-----E--K---- D ----------S-M-E-Q-----R-II----
	Parageobacillus toebii	WP_062677079	-----M-----E--K---- D ----------S-M-E-Q-----Q-II----
	Parageobacillus caldoxylosilyticus	KYD15363	-----------E--E---- D ---T--------M-E-QA----R-II----
	Parageobacillus galactosidasius	WP_012748867	-----M-----E--K---- D ----------S-M-E-Q-----Q-II----
Saccharococcus (1/ 1)	*Saccharococcus thermophilus*	WP_166907272	-----M-----E--K---- D ------------M-E-Q-----Q-II----
Unnamed Geobacillus and Parageobacillus strains	*Geobacillus*	WP_012820478	-----------E------- - ----------E-M------------I----
	Geobacillus sp. JS12	WP_063193543	-----------E------- - ----------E-M------------I----
	Geobacillus sp. TFV-3	WP_160155141	-----------E------- - ----------E-M------------I----
	Geobacillus sp. C56-T2	WP_144972356	-----M------------- - ----------A--------------I----
	Geobacillus genomosp. 3	WP_020958344	------------------- - ------------M------------I----
	Geobacillus sp. 46C-IIa	WP_081210619	------------------- - ------------M-----------II----
	Parageobacillus	WP_012748867	-----M-----E--K---- D ----------S-M-E-Q-----Q-II----
	Parageobacillus genomosp. 1	EZP79091	-----M-----E--K---- N ------------M-E-Q-----R-II----
Other Anoxybacillaceae (0/ >10)	*Anoxybacillus caldiproteolyticus*	WP_181557449	-----------E--K---- D ---S------S-M-E-Q----YK-II----
	Anoxybacillus tepidamans	WP_183255709	-----------E--K---H ---S------S-M---Q----NK-II----
	Anoxybacillus voinovskiensis	WP_183185492	-----------E--K---H -------M--E-M-E-Q----HK-II----
	Anoxybacillus amylolyticus	WP_066322102	-----------E--K--GH --S----M--E-M-E-Q----HK-II----
	Anoxybacillus calidus	WP_181538841	-----------E--Q---H ---S------A-M-E-QA---YK-II----
	Anoxybacillus vitaminiphilus	WP_111646103	-----------E--Q---H ---S------A-M-E-QA---CK-II----
	Anoxybacillus mongoliensis	WP_183244231	-----------E--E-RDA ---L---R-VE-M-E-QA---CK-II----
	Anoxybacillus flavithermus	WP_192948627	-----------E--E-RDT --SL---R-VE-M-E-QA---CK-II----
	Anoxybacillus tengchongensis	WP_183250251	-----------E--E-RDT --SL---R-VE-M-E-QA---CK-II----
	Anoxybacillus thermarum	WP_043964886	-----------E--E-RDA ---L---R-VERM-E-QA---CK-II----
	Thermolongibacillus altinsuensis	WP_132949354	-----------E--K-RD- ---L------E-M-E-QA---YK-II----
Other Bacteria (2/ >100)	*Priestia abyssalis*	WP_078414995	-----------E-----DG --P-------E-M-E--AY--FR-I-----
	Caldibacillus thermolactis	WP_263061333	-----M-----E-QA--D- ----------E---E---YM-I--I-----
	Caldibacillus kokeshiiformis	WP_173660748	-----M-----E-QA--D- ----------E---E---YM-I--I-----
	Bacillus xiapuensis	WP_100332774	-----------E--E---T -------R--ERM-E-Q----SR-I-----
	Caldibacillus pasinlerensis	WP_161920404	-----M-----E-QA--DK ----------E---E---YM-I--I-----
	Bacillus andreraoultii	WP_033827304	-----M-----E-QK--NS -F--------E-M-E-Q-Y--TK-I-----
	Halobacillus halophilus	WP_014641403	-----M-----E-K----Q -FGQ------EMM-E-QSY---RII-I---
	Halobacillus litoralis	WP_225197056	-----M-----E-KK---T --GQ------EMM-E-QSY---RIII----
	Bacillus taeanensis	WP_113808018	-----------E-Q---K- ---S------E-M-E-QAY--YK-M-I---
	Bacillus alveayuensis	WP_044895178	-----------E--E---H ---S------A-M-E-QA---YK-II----
	Halobacillus karajensis	WP_035511720	-----M-----E-KS---K --GQ------E-M-E-QSY---RIMI----
	Halobacillus mangrovi	WP_085026925	-----M-----E-K----K --GQ------EM--E-QSY---RII-----
	Ectobacillus panaciterrae	WP_028400840	----------E---K---- E-Q---------M---Q-Y--TK-II----
	Peribacillus simplex	WP_061141090	-----------E--E---R -------R--EMM-E-QA---SR-I-----
	Halobacillus salinus	WP_079477369	-----M-----E-K----K --GQ------EMM-E-QSY-E-RIII----
	Bacillus mesophilus	WP_163181896	-----------K--E--QN --S-------E-M-E--AYS-YR-II----
	Halobacillus fulvus	RWZ52140	-----M-----E-K----K --GQ-------MM-E-QSY---RII-----
	Mesobacillus maritimus	WP_251435675	-----------E--A--TK ---------VE-M-E-RAY--YR-II----
	Halobacillus faecis	WP_146818493	-----M-----E-KS---K --GQ------EMM-E-QSY---RIMI----
	Halobacillus aidingensis	WP_089654449	I----M-----E-KN---K --GQ------EMM-E-QSY---RIMI----
	Calidifontibacillus oryziterra	WP_017754881	-----------E--E--KQ -F-S---R--E-M-E--A---YR-II----
	Bacillus ectoiniformans	WP_204558166	-----------E--E--KH -------R-VELM-E-Q----SK-II----
	Neobacillus notoginsengisoli	WP_118923786	-----------E--E--KG -------R--ELM-E-Q-YS-YR-II----
	Priestia megaterium	TYR78502	-----M-----N--K-RDN ---S---V--E-M-E-QAY--YR-II----
	Virgibacillus dakarensis	WP_088051809	----------EE-Q----R -M-Q---R--ERM-E--A---NR-II----
	Peribacillus frigoritolerans	WP_268572235	-----------E--E---R -------R--EMM-E-QA---YR-I-----
	Brevibacillus borstelensis	WP_003392022	-----------D--V---- E RMDE------S-----QS---IK-II----
	Brevibacillus gelatini	WP_122906792	-----------E--I---Q E RMDE------S-M-E-QSY--TK-II----

Figure S33: Partial sequence alignment of the NYN domain-containing protein showing a 1 aa insert (highlighted) in a conserved region that is generally exclusively shared by species from the larger *Geobacillus* clade. Note that the homologue for one species within the genus *Geobacillus* is missing. This CSI is also not shared by most other species from the family *Anoxybacillaceae* or other bacteria. A few exceptions in distantly related species are present.

Group	Species	Accession	95	(highlighted)	145
Geobacillus (12/ 12)	*Geobacillus stearothermophilus*	WP_235597858	PVYGTKLTLGLAEAILKEQGIA		NAKLNEIHPDAELLFDKAKVTFFRTIHSI
	Geobacillus thermocatenulatus	WP_025951127	--------I-----L-----VS		G-----V------V----R----------
	Geobacillus thermodenitrificans	WP_008878528	---------------------T		--N--------------------------
	Geobacillus kaustophilus	WP_044731678	-I------------L-----VS		G-----V------V---------------
	Geobacillus subterraneus	WP_033843538	--------I-----L-----VS		G---------V--V---------------
	Geobacillus jurassicus	WP_066229787	--------------L-----VS		G------D-----V------A--------
	Geobacillus zalihae	WP_060788136	-VS		G-----V------V---------------
	Geobacillus thermoleovorans	WP_011230778	--------------L-----VS		G-----V------V---------------
	Geobacillus vulcani	WP_031410328	--------------L-----VS		G-----V------V---------------
	Geobacillus proteiniphilus	WP_074043409	--------------L-----VS		G-----V------V---------------
	Geobacillus uzenensis	OXB88191	-----------V--------VT		--N----------S---------------
	Geobacillus icigianus	WP_033023125	--------I-----L-----VS		G---------V--V---------------
Parageobacillus (6/ 6)	*Parageobacillus thermoglucosidasius*	WP_003251634	-------------S------VT		----I--RS-S-IF---------------
	Parageobacillus yumthangensis	PDM40364.1	-------------S-------T		----I--RS-S-I----------------
	Parageobacillus caldoxylosilyticus	WP_017437030	---------------------T		----H--RSNS-V----------------
	Parageobacillus thermantarcticus	WP_090947975	-------------S-------T		----I--RS-S-IF---------------
	Parageobacillus toebii	WP_015863503	-------------S-------T		----I--RS-S-I----------------
	Parageobacillus galactosidasius	WP_015863503	-------------S-------T		----I--RS-S-I----------------
Saccharococcus (1/ 1)	*Saccharococcus thermophilus*	WP_166908458	---------A-----------T		-T--H--RS-S-V----------------
Unnamed Geobacillus and Parageobacillus strains	*Geobacillus sp. WSUCF1*	EPR26958	--------------L-----VS		G-----V------V---------------
	Geobacillus sp. BCO2	KPC98640	--------I-----L-----VS		G-----V------V----R----------
	Geobacillus sp. FJAT-46040	WP_096225502	--------------L-----VS		G-----V------V---------------
	Geobacillus sp. TFV-3	WP_160156618	-I------------L-----VS		G-----V------V---------------
	Geobacillus sp. C56-T2	WP_144973810	--------I-----L-----VS		G------------V---------------
	Geobacillus sp. Y412MC52	WP_013523423	--------------L-----VS		G-----V---E--V---------------
	Geobacillus sp. DSP4a	WP_050368521	----------------------		-----------------------------
	Geobacillus sp. PA-3	WP_060475759	---------------------T		--N--------------------------
	Geobacillus sp. 46C-IIa	WP_081206920	-----------V--------VT		--N----------S-----A---------
	Geobacillus genomosp. 3	WP_020959403	-----------V---------T		--N----------S---------------
	unclassified Geobacillus	WP_015374438	--------------L-----VS		G-----V------V---------------
	Parageobacillus	WP_064550983	-------------S-------T		----I-VRS-S-IF---------------
	Parageobacillus sp. VR-IP	WP_175243454	---------------------T		----H--RSNS------------------
	Parageobacillus genomosp. 1	WP_043904165	--------------------VM		----H--RS-S-V----------------
Other Anoxybacillaceae (0/ >15)	*Anoxybacillus rupiensis*	WP_183186268	-----------V-EK------S	A	S-P-I--RS-S-IA---TI----------
	Anoxybacillus amylolyticus	WP_066323955	------------QEK---HDTT	T	T-PFIDV-S-S-VV-E--T----------
	Anoxybacillus voinovskiensis	WP_183183130	------------QEK---HDMT	T	T-PFI---S-S-VV-E--T----------
	Anoxybacillus tepidamans	WP_183251540	-----------V-EK---HEVS	T	T-PFI---S-C-VT-E--T----------
	Anoxybacillus caldiproteolyticus	WP_181557239	-------------EK-R-H-VT	S	S-PFV--RS-S-VV-E-------------
	Anoxybacillus calidus	WP_181536758	---A-----A-V-EK---H-VT	V	R-Q---V-S-SVVT-E---M---------
	Anoxybacillus vitaminiphilus	WP_111643779	---A-----A-V-EK---H-VT	V	R-Q---V-S-SVVT-E-V-M---------
	Anoxybacillus flavithermus	WP_041638519	----A----A-M-EK------T	S	K--RI---A-S-IV----V-S--Q-N---
	Anoxybacillus pushchinoensis	WP_091699943	----A----A-M-EK------T	S	K--RI---A-S-IV----I-S--P-N---
	Anoxybacillus mongoliensis	WP_183241468	----A----A-M-EK------T	S	K--RI---A-S-IV----IIS--Q-N---
	Anoxybacillus kestanbolensis	WP_252506253	----A----A-M-EK------T	S	K--RI---A-S-IV-E--I-S--Q-N---
	Anoxybacillus suryakundensis	WP_055441103	----A----A-M-EK------T	S	K--RI---A-S-IV-E--IIS--Q-N---
	Anoxybacillus tengchongensis	WP_183246967	----A----A-M-EK------T	S	K--RI---A-S-IV-E--IIS--Q-N---
	Anoxybacillus thermarum	WP_043967712	----A----A-V-EK------T	S	K--RI--NANS-IV----V-S--Q-N---
	Anoxybacillus ayderensis	KIP22603	----A----A-V-EK------T	S	K--RI--NANS-IV----V-S--Q-N---
	Anoxybacillus gonensis	WP_009362534	----A----A-M-EK------T	S	K--RI--NANS-IV----V-S--Q-N---
	Thermolongibacillus altinsuensis	WP_132948196	---A-----A-V-EK---H-VT	T	K-ERV-VRS-TQ-S-K---IS--Q-N---
Other Bacteria (6/ >50)	*Cytobacillus kochii*	WP_218971969	--------IA--K-KM----FK	G	K---V---S-T--H--HCEIS--K-N---
	Cytobacillus stercorigallinarum	MBD7936668	--------IA--K-KM----FK	G	K-E-V---S-T--H--HCEIS--K-N---
	Bacillus alveayuensis	WP_044893716	---A-----A-V-EK---HNV-	V	RPQ---V-S-SVVN-E-V-I---------
	Cytobacillus oceanisediminis	WP_144542547	---------A--K-K----EYK	G	K-QFI--DS-SK-Q--S-E------S---
	Bacillus canaveralius	PLR84267	--------IA--Q-K-R--EFN	G	T-DFR--NS-SR-N--R-A------N---
	Cytobacillus gottheilii	WP_218970425	---------A--KSK---NEFK	G	K-Q-I--Q-ETQ-E-ET-Q-S----T---
	Bacillus smithii	WP_003352426	---------A--KDR-RQEKGI	E	SV-FYT--S-SR-R--TVD------T---
	Alkalihalobacillus trypoxylicola	WP_061947243	-----------V-EK---A--W	R	Q---HL-NE-STVVAGDTN------N---
	Tepidibacillus fermentans	WP_207893656	-----------V-SK---AN-L	A	ET---LVNG-S-IELGSF-IS----N---
	Mesobacillus jeotgali	WP_218973343	--------IA--KEKM---EFS	G	AVDFH--NA-TV-N--SVS-S--K-N---
	Robertmurraya kyonggiensis	WP_136829408	-I-------A--K--I---EYR	G	K-EFI--NS-TI-E--D-R----K-N---
	Domibacillus epiphyticus	WP_076763103	---------A--KMR-R-ENVK	E	DV-FYTV-S-SR-R--HSE------T---
	Paenibacillus antri	WP_138193914	-----------I--K---ANLL	G	ET-RIL----T--NLGSITAS----N---
	Cohnella herbarum	WP_169279402	-I-A-R-----IQGK---H-LL	A	R-Q--Q--SNSKVT-GTI--S--S-N---
	Peribacillus frigoritolerans	WP_268590454	---------A-IKTK---D-FN	G	R-TFT--DS-SL-S-PQ-A-S----N---
	Mesobacillus selenatarsenatis	WP_041966681	--------IA--KEKM---EFS	G	SVDFH--NA-TV-R--SVS-S--K-N---
	Alteribacillus bidgolensis	WP_091582606	-I--G--AI--LKGK-E-H-LL	R	R----VVRE-Q-IK-K-T-IS----T---
	Peribacillus simplex	WP_125161966	---------A-IKTK---D-FN	G	R-TFT--DS-SL-S-PQ-A-S----N---
	Priestia taiwanensis	WP_188387668	-I---------V-DK-SQV-VL	S	K-N-QV-DA-SIMM----S-S--K-T---
	Paenibacillus soyae	WP_257444883	-----P-----IRGK---H--S		DKN-HT-Q--S-ITLG-LP-A----N---
	Flavobacterium thermophilum	STO11791	----------------------		-----------------------------
	Calidifontibacillus oryziterrae	WP_017753813	-I---------V-DQ-M-A--Q		T-D-R---SETV-E-EH-S-S----T---
	Paenibacillus oryzae	WP_068685741	---A-------I-LK---HRLK		KPE-Y--T-AS--A-GEL-LS----N---
	Sporomusaceae bacterium	MBC8015727	-----Q-AM---QCR---NNV-		-VS--T-K-GD----GSIQ-G-I--S---
	Syntrophomonadaceae bacterium	NLU50352	--------M-IV--K-R-H-LN		G-R--IVK-RDK-TLGPFE-E-I-VS---

Figure S34: Partial sequence alignment of the ribonuclease J protein showing a 1 aa deletion (highlighted) in a conserved region that is generally exclusively shared by species from the larger *Geobacillus* clade. This CSI is also not shared by most other species from the family *Anoxybacillaceae* or other bacteria. A few exceptions in distantly related species are present.

			143		192
Anoxybacillaceae species (36/ 36)	*Anoxybacillus pushchinoensis*	WP_091702361	TSSYHLEVFSLYREHNESLCELMN	T	TFHLHARTLERKKGFITYLKEAEKI
	Anoxybacillus flavithermus	WP_004892513	-------I----------------	-	N------------------------
	Anoxybacillus ayderensis	WP_042535060	-------I----------------	-	A------------------------
	Anoxybacillus tengchongensis	WP_183250001	-------I----------------	-	A-Y----------------------
	Anoxybacillus suryakundensis	WP_055441737	-------I----------------	-	A-Y----------------------
	Anoxybacillus kestanbolensis	MCL9969846	-------I----------------	-	N------------------------
	Anoxybacillus mongoliensis	WP_183243822	-------I----------------	-	N------------------------
	Anoxybacillus thermarum	WP_043964835	-------I----------------	-	N------------------------
	Anoxybacillus gonensis	AKS39450	-------I----------------	-	N------------------------
	Anoxybacillus tepidamans	WP_027409927	-------I----K---D-------	S	H-L---K------------------
	Anoxybacillus voinovskiensis	GGJ64553	-------I----K-----------	S	H-F---K------------------
	Anoxybacillus amylolyticus	WP_066322812	-------I----K---D-------	S	H-L---K------------------
	Anoxybacillus rupiensis	MBS2771263	-------I---DKD--N-------	S	H-G---K------------------
	Anoxybacillus calidus	WP_181537208	-------I----K---D-------	-	H-Y-N-K------------------
	Anoxybacillus vitaminiphilus	WP_111645015	-------I----K---D-------	-	H-Y-N-K------------------
	Anoxybacillus caldiproteolyticus	WP_181556512	-------I----K--SD-------	S	H-F-N-K------------------
	Geobacillus stearothermophilus	WP_049626239	-------I----ED--R-------	S	H-F-N-KM-----------------
	Geobacillus vulcani	WP_031407142	-------I----E---RA------	S	H-L-N-K------------------
	Geobacillus thermodenitrificans	ARA99763	-------I----E---R-------	S	H-F-N-K------------------
	Geobacillus thermocatenulatus	ASS98247	-------I----E---KA------	S	H-F-N-K------------------
	Geobacillus proteiniphilus	WMJ16624	-------I----E---RA------	S	H-F-N-K------------------
	Geobacillus subterraneus	BBW97563	-------I----E---H-------	R	Y-F-N-K------------------
	Geobacillus thermoleovorans	GAJ57498.1	-------I----E---RA------	S	H-F-N-K------------------
	Geobacillus kaustophilus	WP_044732603	-------I----E---RA------	S	H-F-N-K------------------
	Geobacillus jurassicus	WP_066227819	-------I----E---RA------	S	H-F-N-K------------------
	Geobacillus uzenensis	OXB91397	-------I----E---R-------	S	H-F-N-K------------------
	Geobacillus icigianus	MEB3751108	-------I----E---H-------	R	Y-F-N-K------------------
	“*Geobacillus zalihae*”	OQP18672	-------I----E---RA------	S	H-F-N-K------------------
	Parageobacillus thermoglucosidasius	WP_003248094	-------I----E---D-------	S	H-F-N-K------------------
	Parageobacillus thermantarcticus	WP_090948447	-------I----E---D-------	S	H-F---K------------------
	Parageobacillus galactosidasius	WP_015865029	-------I----E---N-------	S	H-F-N-K------------------
	Parageobacillus yumthangensis	PDM39158.1	-------I----E---N-------	S	H-F-N-K------------------
	Parageobacillus caldoxylosilyticus	WP_042410563	-------I----E---N-------	S	H-F-N-K------------------
	Parageobacillus toebii	WP_062677270	-------I----E---N-------	S	H-F-N-K------------------
	Saccharococcus thermophilus	WP_166907923	-------I----E---N-------	S	H-F-N-K------------------
	Thermolongibacillus altinsuensis	TCL47730	-------I----------------	-	A-Y-N--------------------
Other Bacteria (0/ >150)	*Bacillus massiliglaciei*	WP_110928503	-------I----K---DA----L-		H-G-K-K------------------
	Bacillus zhangzhouensis	WP_264008235	-------I----K---DA------		H---NSK-------Y----------
	Metabacillus fastidiosus	WP_066224991	-------I----K---D-------		--Q-NSK------------------
	Neobacillus cucumis	MBI0577656	-------I----K---D-------		--G-NSK------------------
	Metabacillus litoralis	WP_146947791	-------I----K---D-------		--D-NSK------------------
	Metabacillus sediminilitoris	WP_136352427	-------I----K---D-------		--D-NSK------------------
	Bacillus infantis	WP_224902474	-------I----K---D-------		--G-NSK------------------
	Metabacillus bambusae	WP_207980876	-------I----K---D-------		--D-NSK------------------
	Robertmurraya korlensis	WP_066058783	-------I----K---D-------		--G-NSK------------------
	Fredinandcohnia onubensis	WP_099352693	-------I----K---D-------		V-D-N-K------------------
	Bacillus timonensis	WP_010676561	-------I----K---D-------		V-E-N-K------------------
	Bacillus wudalianchiensis	OCA89187	-------I----K---DA------		S-D-NSK------------------
	Bacillus acidicola	WP_066269671	------------K---D-------		S-D-NSK------------------
	Bacillus altitudinis	NQD53723	-------I----K---DA------		Q---NSK-----------M------
	Metabacillus crassostreae	WP_204955709	-------I----K---D-------		--E-NSK------------------
	Neobacillus pocheonensis	MCM2536087	-------I----K---D-------		--G-NSK------------------
	Bacillus weihaiensis	WP_072578449	-------I----K---D-------		--D-NSK------------------
	Fredinandcohnia humi	WP_057999083	-------I----KD--D-------		V-D-N-K------------------
	Bacillus salipaludis	WP_133333981	------------K---D-------		--G-NSK------------------
	Cytobacillus depressus	WP_151537177	------------K---D-------		--D-NSK------------------
	Metabacillus lacus	WP_154307491	-------I----K---D-------		----NSK------------------
	Heyndrickxia vini	WP_202779311	-------I----K---D-------		F-Q-NSK------------------
	Aquibacillus sediminis	WP_138418368	-------I-N--Q---D-------		S-G---KI----------I------
	Weizmannia ginsengihumi	WP_025727129	-------I----K---D---K---		A-Q-NSK------------------
	Metabacillus idriensis	WP_070876434	-------I----K---D-------		S---NSK-------Y----------
	Priestia megaterium	WP_168245652	-------I----K---D-------		--G-NSK-------Y----------
	Neobacillus endophyticus	WP_173059821	-------I----K---D-------		--G-NSK-------Y----------
	Siminovitchia thermophila	WP_205178737	-------I--I-K---D-------		S-Q-NSK-----N------------
	Neobacillus fumarioli	WP_066366049	-------I----K---D-------		--G-NCK-------Y----------
	Bacillus suaedaesalsae	WP_204204361	-------I----K---D-------		E-E-NSK-------Y----------
	Metabacillus kandeliae	WP_231790522	-------I----K---D-------		----NSK-------Y----------
	Bacillus tuaregi	WP_071393609	-------IA---K---D-------		S-D-NSK------------------
	Heyndrickxia sporothermodurans	WP_066230374	-------I----K---D-------		F---NSK------------------
	Peribacillus cavernae	WP_126863295	------------K---DA------		Q-G-K-K-------Y----------
	Lederbergia panacisoli	WP_257582177	-------I----K---D-------		D-G-NSK-----N------------
	Peribacillus frigoritolerans	TDL78866	-------I----K---D-------		----NSK-------Y----------
	Lederbergia citrisecundus	WP_213110910	-------I----K---D-------		D-G-NSK-----N------------
	Bacillus australimaris	WP_060698392	-------I----K---DA------		Q-Q-NSK-------Y---M------
	Lederbergia citri	MBS4195399	-------I----K---D-------		D-G-NSK-----N------------
	Lederbergia wuyishanensis	WP_244681295	-------I----K---D-------		D-G-NSK-----N------------

Figure S35: Partial sequence alignment of the DNA-binding protein WhiA showing a 1 aa insert (highlighted) in a conserved region that is generally exclusively shared by species from the family *Anoxybacillaceae*. This CSI is also not shared by most other bacteria.

			212		267
Anoxybacillaceae species (35/ 35)	*Anoxybacillus pushchinoensis*	WP_091700607	EEIFHLVSLLKPGHLGNAEQFQKRY		GKLRTLHDDAHLKELIRKVMIRHRRHDTGIE
	Anoxybacillus kestanbolensis	WP_077428750	-----------------------K-		-------------------------------
	Parageobacillus yumthangensis	PDM41318.1	----N--------------Y-A-T-		--T-S-QTNE---A--N-----N--V-----
	Anoxybacillus ayderensis	MCL6617922	-------------------------		-------------------------------
	Anoxybacillus flavithermus	WP_003394071	-------------------------		-------------------------------
	Anoxybacillus suryakundensis	WP_055440953	-------------------------		---------V---------------------
	Anoxybacillus mongoliensis	WP_183240803	-------------------------		---------V---------------------
	Anoxybacillus tengchongensis	WP_183246295	-------------------------		---------V---------------------
	Anoxybacillus thermarum	WP_043964043	-------------------------		-R-----------------------------
	Anoxybacillus gonensis	WP_009373932	-------------------------		-------------------------------
	Anoxybacillus amylolyticus	WP_066326182	------------------SY-AET-		--G---QT-E---V-VN-----N--I-----
	Anoxybacillus rupiensis	MBB3906829	------------------TY-AET-		--E-S-QT-E---A-VN-----N--AN----
	Anoxybacillus tepidamans	WP_027407775	------------------SY-AET-		--T-S-QT-E---A-VN-----N--I-----
	Anoxybacillus voinovskiensis	WP_183183869	------------------SS-AET-		--G---QT-E---A-VN-----N--L-----
	Anoxybacillus calidus	WP_181535303	------------------AY-TEK-		--T-S--A-E---S-VN-----N--T-----
	Anoxybacillus vitaminiphilus	WP_111644203	------------------AY-TEK-		--M-S--A-E---A-VN-----N--M-----
	Anoxybacillus caldiproteolyticus	WP_199425705	----N-------------AY-TEM-		--K-S-QT-E---A-VN-----N--A-----
	Geobacillus stearothermophilus	WP_053532197	----N----------------A-T-		--T-AVQANE---A-VN-----N--A--P--
	Geobacillus subterraneus	WP_033844173	----N-----------S----ANT-		--T-AVQAND---A-VN-----N--A--P--
	Geobacillus zalihae	WP_060787690	----N-----------SS---A-T-		--T-AVQTND---A-VN-----N--A--P--
	Geobacillus jurassicus	WP_066230519	----N----------------A-T-		--T-AVQTNE---A-VN-----N--A--P--
	Geobacillus proteiniphilus	WP_074043494	----N----------------A-T-		--T-AIQTND---A-VN-----N--A--P--
	Geobacillus kaustophilus	WP_044730847	----N----------------A-T-		--T-AVQTND---A-VN-----N--A--P--
	Geobacillus thermodenitrificans	WP_011887795	----N----------------A-T-		--T-AVQTND---A-VN-----N--A--P--
	Geobacillus thermocatenulatus	WP_025948958	----N-----------SS---A-T-		--T-AVQTND---A-VN-----N--A--P--
	Geobacillus thermoleovorans	WP_011231908	----N-----------SS---A-T-		--T-AVQTND---A-VN-----N--A--P--
	Geobacillus vulcani	WP_ 031408303	----N-----------SS---A-T-		--T-AVQAND---A-VN-----N--A--P--
	Geobacillus icigianus	WP_033019001	----N-----------S----ANT-		--T-AVQAND---A-VN-----N--A--P--
	Parageobacillus thermoglucosidasius	WP_125009489	--M-N--------------Y-A-T-		--T---QTNE---A-VN-----N--V-----
	Parageobacillus galactosidasius	WP_089097910	----N--------------Y-A-T-		--T-S-QTNE---A--N-----N--I-----
	Parageobacillus caldoxylosilyticus	WP_026078459	----N--------------Y-A-T-		--T-S-QTNE---A-VN-----N--A-----
	Parageobacillus thermantarcticus	WP_090948596	----N--------------Y-A-T-		--T-A-QTNE---A-VN-----N--V-----
	Parageobacillus toebii	WP_205424574	----N--------------Y-A-T-		--T-S-QTNE---A--N-----N--V-----
	Saccharococcus thermophilus	WP_166910241	----N--------------Y-A-T-		--T-S-QTNE---A-VN-----N--A-----
	Thermolongibacillus altinsuensis	WP_132948593	-----------------VNH--EK-		--M-S-NE-E---T--------N--N-----
Other Bacteria (0/ >100)	*Peribacillus simplex*	WP_251430957	------------------SL-SEK-	K	--G-NII--E-----VNT----N--A-----
	Bacillus massiliglaciei	WP_110927431	----------------S-SL-SEK-	K	--G-S----Q------NT----N--A-----
	Rossellomorea vietnamensis	WP_148941661	-----------------ESG-SEK-	K	KGS-SVGN-------VN-----N--S-----
	Peribacillus huizhouensis	WP_182501560	------------------AL-SEK-	K	--G--IYE-E---K-VNT----N--A-----
	Bacillus cihuensis	WP_028391779	------------------AL-SEK-	K	--G--IYE-E---K-VNT----N--A-----
	Rossellomorea aquimaris	WP_044340024	-----------------ESG-SD--	K	KGD---QE-E---S--N-----N--S-----
	Peribacillus muralis	WP_241577300	------------------SL-SEK-	K	--GKNII--E-----VNT----N--A-----
	Cytobacillus firmus	WP_035329977	S---N------------ESA-YEN-	K	KDS-S-N--------VN-----N--A-----
	Cytobacillus oceanisediminis	WP_217026200	S---N------------ESA-YEN-	K	KDS-S-N--------VN-----N--A-----
	Peribacillus deserti	WP_204543981	D-----------------SL-AE-F	K	--K-D-NE-KR----VN-----N--A-----
	Sporosarcina globispora	WP_053434422	S---N------------ESA-YEN-	K	KDS-S-N--------VN-----N--A-----
	Neobacillus massiliamazoniensi	CRK84112	----N------------ETT-YEK-	K	RDS-S----E----IVN-----N--A-----
	Peribacillus frigoritolerans	WP_249594817	------------------SL-SEK-	K	--G-NIIE-E-----VNT----N--A-----
	Bacillus testis	WP_050615745	D----------------SSM-AQQ-	K	--G-EIY--------VNA--L-N---E----
	Peribacillus saganii	WP_117327875	D-----------------AL-SEKF	K	--G-NII--K-----VNT----N--A-----
	Rossellomorea arthrocnemi	WP_201713472	-----------------ETG-TD--	K	KGD---QE-E---N--N-----N--G-----
	Bacillus massilionigeriensis	WP_075982284	----N------------ESV-YEK-	K	KDS-SI---E-----VN-----N--S-----
	Bacillus haikouensis	QWC21648	-----------------ESG-TD--	K	KGD--V---DS-NQ--N-----N--S-----
	Peribacillus acanthi	WP_108670820	------------------AY-SEKF	K	--G-NINE--Y----VN-----N--G-----
	Peribacillus kribbensis	WP_026692913	D-----------------SL-AEKF	K	--K-NMNE-QR----VN-----N--A-----
	Priestia megaterium	WP_107918120	----N------------KDY-EELF	S	A-K-S-QN-------VN-----N--------
	Priestia aryabhattai	WP_226554897	----N------------KDY-EELF	S	A-K-S-QN-------VN-----N--------
	Bacillus salacetis	WP_119548298	-----------------ESA-SEK-	K	KGS-SSGN-D-----VN-----N--S-----
	Bacillus massiliogorillae	WP_042345997	D-----------------SL-S-T-	K	--G-QINE-E-----VNA----N---E----
	Neobacillus rhizophilus	WP_213120193	----N------------ETV-YEK-	K	RDS-S-N--Q-----VN-----N--A-----
	Cytobacillus gottheilii	WP_080845152	S---N------------ETA-YEK-	K	KDA-S-D--D-----VN-----N--A-----
	Bacillus mediterraneensis	WP_071460109	----N------------E-A-FD--	K	KAS-SV---E--R--VN-----N--G-----
	Bacillus infantis	WP_148977194	S---N------------ETA-YEK-	K	KDS-S-D-N------VN-----N--A-----
	Bacillus methanolicus	WP_004437792	----N------------ESA-FEK-	K	RDS-S-N--K--R--VN-----N--A-----
	Cytobacillus luteolus	MBP1942708	----N------------QSY-EES-	N	A-E-S-DG-K-----VN-----N--D-----
	Neobacillus novalis	WP_066089848	----N-----------SETS-YEK-	K	RDA-S-N--E-----VN-----N--A-----
	Peribacillus asahii	WP_252286715	------------------KL-AEK-	K	--G--IQE-Q-----VNT----N--A--K--
	Neobacillus dielmonensis	WP_042458338	----N-----------SQTA-YEK-	K	RDA-S-N--K-----VN-----N--A-----
	Peribacillus butanolivorans	WP_098176740	------------------TL-SEK-	K	--G-NVIE-E-----VNT----N--A-----
	Falsibacillus albus	WP_121678940	D-V--------------ESI-AEK-	K	KGD-SIQE----R--VN-----N--A-----
	Bacillus fonticola	WP_170007638	----N------------LAD-ADK-	D	RAE-S-QQE-------N-----N--E-----
	Neobacillus piezotolerans	WP_115453771	----N------------ESS-FDK-	R	KDS--VA---N----VN-----N--G-----
	Mesobacillus jeotgali	WP_192470914	----N-----------SETA-FDK-	K	KDA-SVN--E-----VN-----N--S-----

Figure S36: Partial sequence alignment of the SNF2-related protein showing a 1 aa deletion (highlighted) in a conserved region that is generally exclusively shared by species from the family *Anoxybacillaceae*. Note that the homologue for *Geobacillus uzenensis* is missing. This CSI is also not present in most other bacteria.

Group	Species	Accession	238		293
			NMLRQPEFSDIDKLRALFNIIEQEKEFYRLLRK	H	NQQGIQVKIGTENDVEGMENCS
Anoxybacillaceae species (36/ 36)	*Anoxybacillus pushchinoensis*	WP_091703604	NMLRQPEFSDIDKLRALFNIIEQEKEFYRLLRK	H	NQQGIQVKIGTENDVEGMENCS
	Parageobacillus yumthangensis	PDM41387.1	---N-------Q-V-S-MKM-----D-------	-	-RK----T--R--QLS------
	Anoxybacillus flavithermus	WP_192952836	---------------------------------	-	----------------------
	Anoxybacillus rupiensis	MED4879058.1	---N-------E-V-P-L-------DI------	Q	S-K-VK-S--R--ELH------
	Anoxybacillus ayderensis	MCL6617763	----------------L-V--------------	-	K---------------------
	Anoxybacillus thermarum	WP_043965494	----------------L-V--------------	-	K---------------------
	Anoxybacillus mongoliensis	WP_183240963	--------N---------V--------------	-	K---V-----------------
	Anoxybacillus suryakundensis	WP_055441021	--------N---------V--------------	-	K---V-----------------
	Anoxybacillus tengchongensis	WP_183246176	--------N---------V--------------	-	K---V-----------------
	Anoxybacillus kestanbolensis	OOE04986	---------------------------------	-	----------------------
	Anoxybacillus gonensis	AKS38970	----------------L-V--------------	-	K---------------------
	Anoxybacillus amylolyticus	WP_066326267	---S-------Q-I-S-MTM-----D-------	-	R-K----T--R--QLR------
	Anoxybacillus voinovskiensis	WP_183183944	---S-------Q-I-S-MAM-----D-------	-	R-K---IT--R--QLS------
	Anoxybacillus tepidamans	WP_183253779	---S-------Q-V-S-MT------D-------	N	SKK----T--R--QLS------
	Anoxybacillus vitaminiphilus	WP_111644280	---S----N--E-V-SIMTV-----D--N----	-	-K-----T--K--QLR------
	Anoxybacillus calidus	WP_181535166	---S----N--E-V-SIMTM-----D--N----	-	-K-----T--K--QLR------
	Anoxybacillus caldiproteolyticus	WP_181554461	---I-------Q-V-S-MMM-----D-------	-	SKK----T--R--QLS------
	Geobacillus stearothermophilus	Q45550	---N----N--Q-I-P-MKM-----D-------	-	-RK----T--R--QLSE-----
	Geobacillus kaustophilus	WP_044730787	---N-------Q-V-P-L-------DI------	Q	--K-VR-S--H--ELR------
	Geobacillus thermodenitrificans	ABO67787	--FN-------Q-V-P-L-------DI------	Q	S-K-VR-S--R--ELS------
	Geobacillus subterraneus	WP_168368814	---N-------Q-V-P-L-------DI------	Q	--K-V--S--R--ELS------
	Geobacillus thermoleovorans	WP_068895601	--FN-------Q-V-P-LD------DI------	Q	T-K-VR-S--H--ELR------
	Geobacillus thermocatenulatus	WP_025948895	---N-------Q-V-P-LD------DI------	Q	T-K-VR-S--H--ELR------
	Geobacillus vulcani	WP_031408161	---N-------Q-V-P-L-------DI------	Q	T-K-VR-S--H--ELR------
	Geobacillus jurassicus	WP_066234386	---N-------Q-V-P-L-------DI------	Q	T-K-VR-S--H--ELR------
	Geobacillus uzenensis	OXB90934	---N-------Q-V-P-L-------DI------	Q	--K-V--S--R--ELS------
	Geobacillus proteiniphilus	OK091283	---N-------Q-V-P-L-------DI------	Q	--K-VR-S--H--ELR------
	Geobacillus icigianus	WP_033018915	---N-------Q-V-P-L-------DI------	Q	--K-VR-S--H--ELR------
	Geobacillus zalihae	QNU25679	---N-------Q-V-P-LD------DI------	Q	T-K-VR-S--H--ELR------
	Parageobacillus thermantarcticus	WP_090947440	---N-------Q-V-P-MKM-----D-------	-	-RK----T--R--QLS------
	Parageobacillus thermoglucosidasius	WP_073519233	---N-------Q-V-S-MKM-----D-------	-	-RK----T--R--QLS------
	Parageobacillus caldoxylosilyticus	WP_042406309	---N-------Q-I-P-MKM-----D-------	-	-RK----T--R--QLS------
	Parageobacillus toebii	WP_015864549	---N-------Q-V-S-MKM-----D-------	-	-RK----T--R--QLS------
	Parageobacillus galactosidasius	OXB92457	---N-------Q-V-S-MKM-----D-------	-	-RK----T--R--QLS------
	Saccharococcus thermophilus	WP_208404521	---N-------Q-I-P-MKM-----D-------	-	-RK----T--R--QLS------
	Thermolongibacillus altinsuensis	WP_132948668	----------E-V-S-M----------------	-	Q--------K--E-K---D--
Other Bacteria (4/ >100)	*Anoxybacillus sediminis*	UFJ60103.1	IMD----R-V--VKDILELL--NDQLVH-FGP		PSE--T-R--Q--QLDA-KE--
	Bacillus timonensis	MCA1031812	---S----H--Q-I-S-LTM--N-----E---T		--T--NI---R--Q-SA-----
	Cytobacillus luteolus	WP_193535505	---S----N--K-I-S-LTM--------E---T		--T--SI---R--R-SA-----
	Cytobacillus suaedae	QOR65589	---S----N--K-I-S-LTM--------E---T		--T--SI---R--R-SA-----
	Litchfieldia salsa	WP_090855029	---S----H-VQ-I-S-LTM--EKQD--Q----		S-T--NI---R--QFSA-----
	Litchfieldia alkalitelluris	WP_078545540	---S----H-VQ-I-S-LTM--EKQD--Q--K-		S-T--NI---R--QFSA-----
	Streptococcus pneumoniae	CJG63528	---S----H--Q-V-S-LTM-DN-A---DI--H		K-V-------R--SSTA--D--
	Bacillus cereus	TKJ00067	---S----H--Q-V-S-LTM-DN-A---DI--H		K-V-------R--SSTA--D--
	Margalitia shackletonii	WP_055739224	---N----H--Q-I-S-MDM--R-QG--D-F-H		IPE--H----K--KLTE-----
	Bacillus anthracis	MBC8937289	---S----H--H-V-S-LTM-DN-A---DI--H		K-V-------R--SATA--D--
	Metabacillus litoralis	WP_121662894	---S-------GRI-S-LTM------L-G--KA		-SA--SI---K---LSA-----
	Bacillus mesophilum	WP_151573292	---S----H----V-N-M-M-----G--D-I-H		HHS-------R--NN-AID---
	Cytobacillus gottheilii	WP_080844953	---S----H----V-N-M-M-----G--D-I-H		HHS-------R--NN-AID---
	Priestia koreensis	WP_053402063	---N----N--N-V-S-LTM----DQVT---HS		-KL-------R--QESA-----
	Bacillus paranthracis	WP_163118634	---S----H--Q-V-S-LTM-DN-A---DI--H		K-V-------R--SATA--D--
	Bacillus benzoevorans	WP_184526500	---S----N----V-N-LKL----DD--DI---		-PT--HIR--R--NNTA--D--
	Peribacillus kribbensis	WP_026692842	---Q----N-L--IKT-LSM-D--QDM-K--S-		-PA--H----R--KISA-----
	Cytobacillus eiseniae	WP_066396086	---N----H-FE-I-S-MTM----DSI-E-V-E		-RS--NI---R--NNSA-D---
	Bacillus clarus	WP_042979952	---S----H--Q-V-S-LTM-DN-A---DI--H		K-V-------R--SSTA--D--
	Bacillus pacificus	WP_262464573	---S----H--Q-V-S-LTM-DN-A---DI--H		K-V-------R--SSTA--D--
	Cytobacillus oceanisediminis	WP_110066108	---S----H--E-IKN-M-M-----GI-D-I--		-KA--NI---R--NNSA-----
	Cytobacillus firmus	WP_264738600	---S----H--E-VKN-M-M-----GI-D-I--		-KS--NI---R--NNSA-----
	Bacillus infantis	MCA1039059	---S----H--E-I-N-M-M----ESI-D-I--		DKS--NI---R--ENSA-----
	Bacillus wiedmannii	WP_075307982	---S----H--H-V-S-LTM-DN-A---DI--H		K-V----N--R--SATA--D--
	Niallia endozanthoxylica	WP_150440011	---N----HN-E-V-N-LDL-----D--DII--		-PS--H----R--VNSAL-D--
	Bacillus acidicola	WP_066262050	-I-N----N--H-I-S-MEM--G--GV-E-FKH		TPA--H----K--QLSE--D--
	Ectobacillus panaciterrae	WP_028399256	---A----H--H-V-S-L-M----Q---H---N		KEI-------R--GASA--D--
	Bacillus mycoides	WP_144550900	---S----H--Q-V-S-LTM-DN-AA--DI--H		K-V-------R--SSTA--D--
	Peribacillus asahii	WP_252290872	--------H-VH-IHS-MSM----QG--E-FKQ		-PA--N----R--QISAL-D--
	Bacillus tropicus	WP_087986343	---S----H--H-V-S-LTM-DN-A---DI--H		K-V-------R--SATA--D--
	Metabacillus iocasae	WP_239583331	--FS----N-V--M---LTM----QQV-QM--A		-KA--DI---R--E-SA-----
	Mesobacillus maritimus	WP_221872111	---N----N----I-S-MAM----DGISQ-I--		-PK--N----R--NNSA-D---
	Sporosarcina globispora	WP_053434340	---S----H--E-VKN-M-M----EGI-D-I--		-KA--NI---R--NNSA-----
	Bacillus massiliglaciei	WP_110926922	---S----H----V-S-MSM-----G--D-MQQ		-PS--T----R--QNTVL----
	Mesobacillus subterraneus	WP_125481994	-I-S----N-VE-I-S-M-M----DGI-Q----		--A--NI-------NSA-----
	Jeotgalibacillus proteolyticus	WP_104056954	---N-------E---L-MDM--R-ENL-K-I-P	S	DGS-------K--E-IA-----

Figure S37: Partial sequence alignment of the heat-inducible transcriptional repressor HrcA protein showing a 1 aa insert (highlighted) in a conserved region that is generally exclusively shared by species from the family *Anoxybacillaceae*. This CSI is also absent in most other bacteria. A few exceptions in distantly related species are present.

Group	Species	Accession	101		150
Anoxybacillaceae species (35/ 35)	*Anoxybacillus pushchinoensis*	WP_091699997	LVETSVRIVKERKMNEVKDRAQELANKRLVELLVPGKAKQPM		KNPFELLF
	Anoxybacillus kestanbolensis	WP_077429199	--		--------
	Parageobacillus yumthangensis	PDM41257.1	-------L-------------EQQ------------Q--T-		---L----
	Anoxybacillus gonensis	WP_035065868	-----------K------EQ-EQ-------------T----		--------
	Anoxybacillus flavithermus	WP_004891077	-----------K------------------------------		--------
	Anoxybacillus tengchongensis	WP_183247052	-----------K------EQ-EQ-------------T----		--------
	Anoxybacillus mongoliensis	WP_183241573	-----------K------EQ-EQ-------------T----		--------
	Anoxybacillus thermarum	WP_043964772	-----------K------EQ-EQ-------------T----		--------
	Anoxybacillus suryakundensis	WP_055441149	-----------K------EQ-EQ-------------T----		--------
	Anoxybacillus ayderensis	WP_021094218	-----------K------EQ-EQ-------------T----		--------
	Anoxybacillus amylolyticus	WP_066323773	----A--L-------D-----EKQ------------Q--T-		---L----
	Anoxybacillus rupiensis	MBS2771000	-------L------Y------EQQ-----I-------P--TI		---L-M--
	Anoxybacillus tepidamans	WP_183251674	-------L-------------EQQ------------Q--T-		---L----
	Anoxybacillus voinovskiensis	WP_183183065	----A--L-------D-----EKQ------------Q--T-		---L----
	Anoxybacillus calidus	WP_181536694	-------L---------R---E-Q-----I-------Q--T-		--------
	Anoxybacillus vitaminiphilus	WP_111643713	-------L---------R---E-Q------------Q--T-		--------
	Anoxybacillus caldiproteolyticus	WP_199426026	-------L-------------EQQ------------Q--T-		---L----
	Geobacillus stearothermophilus	WP_047819262	---------------------EQQ------------Q--TI		---L----
	Geobacillus kaustophilus	WP_044733181	-------L-------------EQQ------------P--TI		---L----
	Geobacillus subterraneus	WP_033844851	-------L-------------EQQ------------P--TI		---L----
	Geobacillus jurassicus	WP_066230151	-------L-------------EQQ--R---------P--TI		---L----
	Geobacillus thermoleovorans	AEV18718	-------L-------------EQQ------------P--TI		---L----
	Geobacillus thermocatenulatus	AST00949	---------------------EQQ------------Q--TI		---L----
	Geobacillus zalihae	OQP17915	---------------------EQQ------------P--TI		---L----
	Geobacillus proteiniphilus	WMJ18080	-------L-------------EQQ------------P--TI		---L----
	Geobacillus icigianus	MEB3749557	-------L-------------EQQ------------P--TI		---L----
	Geobacillus thermodenitrificans	ARA97172	-------L------S------EQQ------------Q--TI		---L----
	Geobacillus uzenensis	OXB88551	-------L------S------ERQ------------Q--TI		---L----
	Geobacillus vulcani	WP_031410437	-------L-------------EQQ------------P--TI		---L----
	Parageobacillus toebii	WP_062678229	-------L-------------EQQ------------Q--T-		---L----
	Parageobacillus caldoxylosilyticus	WP_218687752	-------L-------------EQQ-----------RQ--TI		---L----
	Parageobacillus thermantarcticus	WP_090948035	-------M---------R---EQQ------------Q--T-		---L----
	Parageobacillus thermoglucosidasius	MBY6267652	-------M-------------EQQ------------Q--T-		--------
	Saccharococcus thermophilus	WP_166908335	-------L-------------EQQ-----------RQ--TI		---L----
	Thermolongibacillus altinsuensis	WP_132948133	----A--M---K------------------------S--AV		--------
Other Bacteria (2/ >50)	*Fictibacillus nanhaiensis*	WP_251322345	-------L---Q-------K-EQN------------KTETN	Y	---L-MF-
	Fictibacillus arsenicus	WP_066289392	-------L---L-------K-EQN------------KTETN	Y	---L-MF-
	Fictibacillus halophilus	WP_198767029	-------L---L---D---K-EQN------------KTETS	F	---L-MF-
	Metabacillus idriensis	WP_070877242	-------L---E---A---SA-E-N--R---------RK--TS	Y	---L-M--
	Metabacillus indicus	WP_029565597	-A-----L---E-------AL-E-N------------K--TS	Y	---L-M--
	Bacillus subtilis	WP_141770850	-------LI--E------EQ-E-N----I-R------K--SG	V	-----MF-
	Bacillus altitudinis	NQD51977	----AI-L---E--KD-QEE-EKQ------H------K-SQS	V	-----M--
	Fictibacillus gelatini	WP_035349721	-----I-L---Q--LA---K-E-N------------Q-ETG	Y	---L-MF-
	Bacillus licheniformis	WP_257008079	-------L---E------GI-E-N------R-----RK--TG	A	-----M--
	Bacillus sonorensis	WP_255265699	-------L---E------GI-E-N------R-----RK--TG	A	-----M--
	Fictibacillus aquaticus	WP_094250823	-----I-L---I--H----S-E-N------------K-ENV	M	---L-MF-
	Fictibacillus solisalsi	WP_090233249	-----I-L---QR-H----K-E-N------------Q-ESS	Y	---L-MF-
	Bacillus paralicheniformis	WP_165426969	-----I-L---E------GI-E-N------R-----RK--TG	A	-----M--
	Fictibacillus macauensis	WP_007202535	-----I-L---QR-L----K-E-N-----I-------Q-ESS	M	---L-M--
	Bacillus mojavensis	WP_010334242	-------LI--E-I----EQ-E-N----I-R------K--SG	V	-----MF-
	Bacillus vallismortis	WP_121642565	-------LI--E-I-----Q-E-N----I-R------K--SG	V	-----MF-
	Litchfieldia alkalitelluris	WP_078547147	-------L---E-IV--Q---E-N--R---------QK--SS	Y	---L-M--
	Bacillus intestinalis	KFK80260	-------LI--E------EQ-E-N----I-R------K--SG	V	-----MF-
	Metabacillus fastidiosus	WP_066225385	-T--AI-L---E------GT-E-N--R---------K--TA	Y	---L-M--
	Bacillus rugosus	WP_166852976	-------LI--E------EQ-E-N----I-R------K--SG	V	-----MF-
	Bacillus stercoris	WP_227098544	-------LI--E------EQ-E-N----I-R------K--SG	V	-----MF-
	Bacillus glycinifermentans	WP_048354621	-------L---E------GI-E-N------R-----RK--AG	A	-----M--
	Bacillus cabrialesii	WP_263458279	----A--LI--E------EQ-E-N----I-R------K--SG	V	-----MF-
	Sutcliffiella cohnii	AST92064	--------I--ER-EA---K-E-N--T---------Q-ETN	Y	-----M--
	Bacillus halotolerans	WP_059335796	-------LI--E-I----EQ-E-N----I-R------K--SG	V	-----MF-
	Metabacillus niabensis	WP_174881106	----A--L---E--TD--GA-E-N-------------K--ST	V	---L-M--
	Peribacillus kribbensis	WP_026692498	-------LI--E--LS-----EQN--S--------SAK--AN	F	---L-M--
	Bacillus pumilus	WP_230030146	----AI-L---E--KD-QEE-EKQ------H------K-SQS	V	-----M--
	Bacillus methanolicus	WP_155815631	-------L---E---S--E--E-H--R--------SAK-SSN	Y	---L-M--
	Bacillus rhizoplanae	WP_230573484	-----------E--VH---K-E-Q--Q-I--I---H-Q-ESG	F	---L-M--
	Bacillus safensis	WP_044330661	----AI-L---E--KD-QEE-EKQ------H------K-SQS	V	-----M--
	Alkalihalobacterium elongatum	WP_216827994	--D--I-----E---S-R-K-E-Q--Q-I-D----SAK--TQ	Y	---L-M-
	Priestia megaterium	RBN39193	-------L---E---G-R---E-N----I------S-K--QS	F	-----MI
	Bacillus carboniphilus	WP_226538343	-------L---E--EK--NQ-E-Q-----------S-R--TS	Y	---L-M-
	Sutcliffiella horikoshii	WP_064101119	-------L---E--AS---K-L-N----I--------Q-NTS	Y	---L-MF-
	Bacillus mediterraneensis	WP_071459436	-T---I-----E--V------E-N--Q--------SLK--SF		---LDM--
	Mesobacillus zeae	WP_119111556	-T-----L---E--QD--E--EGN--R--------SSK-HGY		---LDM--

Figure S38: Partial sequence alignment of the HslU-HslV peptidase ATPase subunit protein showing a 1 aa deletion (highlighted) in a conserved region that is generally exclusively shared by species from the family *Anoxybacillaceae*. Note that the homologue for *Parageobacillus galactosidasius* is missing. This CSI is also absent in most other bacteria. A few exceptions in distantly related species are present.

			669		719
	Anoxybacillus pushchinoensis	WP_091702253	LNEVLQAVNETKTNISAVSGRTD	H	RNKMATIHMTIFIHNVSHLQKVVERIK
	Anoxybacillus flavithermus	WP_012574407	-----------------------	-	---------------------------
	Anoxybacillus thermarum	WP_043966880	-----------------------	-	---------------------------
	Anoxybacillus mongoliensis	WP_183241154	-----------------------	-	---------------------------
	Anoxybacillus gonensis	WP_035067181	-----------------------	-	---------------------------
	Anoxybacillus tengchongensis	WP_183250271	-----------------------	-	---------------------------
	Anoxybacillus suryakundensis	WP_055441848	-----------------------	-	---------------------------
	Anoxybacillus ayderensis	WP_085788096	-----------------------	-	---------------------------
	Anoxybacillus kestanbolensis	OOE02335	-----------------------	-	---------------------------
	Parageobacillus yumthangensis	PDM41451.1	-------------D-------S-	-	---I-------A-R-I-------D---
	Anoxybacillus amylolyticus	WP_066326415	-------------D-------S-	-	---I-------A-R-I-----I-----
	Anoxybacillus tepidamans	WP_183253881	-------------D-------S-	-	---I-------A---------------
	Anoxybacillus rupiensis	MBS2771531	-------------D-------S-	-	---I-------A-Q-------I-----
	Anoxybacillus voinovskiensis	WP_183183639	-------------D-------S-	-	---I-------A-R-I-----------
	Anoxybacillus vitaminiphilus	WP_111645848	-------------D-------S-	-	---I-----A-S---L-----------
	Anoxybacillus calidus	WP_181535025	-------------D-------S-	-	---I-----A-S---L-----------
	Anoxybacillus caldiproteolyticus	WP_181554533	-------------D-------S-	-	---I-------A-Q-------------
Anoxybacillaceae species (36/ 36)	*Geobacillus stearothermophilus*	KFL14738	-----------R-D-------S-	-	-H-I-------A---I-----------
	Geobacillus kaustophilus	WP_042380606	-----------R-D-------S-	-	-H-I-------A---L-----------
	Geobacillus jurassicus	WP_066228691	-----------R-D-------S-	-	-H-I-------A---L-----------
	Geobacillus subterraneus	WP_168368830	-----------R-D-------S-	-	-H-I-------A---I-----------
	Geobacillus vulcani	WP_031408062	-------I---R-D-------S-	-	-H-I-------A---L-----------
	Geobacillus thermodenitrificans	MEC5187265	-----------R-D-------S-	-	-H-I-------A---I-----------
	Geobacillus thermoleovorans	MED4973820	-----------R-D-------S-	-	-H-I-------A---L-----------
	Geobacillus proteiniphilus	WMJ17046	-----------R-D-------S-	-	-H-I-------A---L-----------
	Geobacillus thermocatenulatus	ASS98732	-----------R-D-------S-	-	-H-I-------A---L-----------
	Geobacillus zalihae	WP_301099951	-----------R-D-------S-	-	-H-I-------A---L-----------
	Geobacillus icigianus	WP_033018073	-----------R-D-------S-	-	-H-I-------A---LG----------
	Geobacillus uzenensis	OXB90875	-----------R-D-------S-	-	-H-I-------A---I-----------
	Parageobacillus thermoglucosidasius	WP_042383460	-------------D-------S-	-	---I-------A-R-I-----I-D---
	Parageobacillus galactosidasius	WP_089097943	-------------D-------S-	-	---I-------A-R-I-------D---
	Parageobacillus toebii	WP_062677167	-------------D-------S-	-	---I-------A-R-I-------D---
	Parageobacillus thermantarcticus	WP_090947499	-------------D------KS-	-	---I-------A-R-I-------D---
	Parageobacillus caldoxylosilyticus	WP_017435556	-------------D-------S-	-	---I---N---A-R---------D---
	Saccharococcus thermophilus	WP_166910493	-------------D-------S-	-	---I---N---A-R---------D---
	Thermolongibacillus altinsuensis	WP_132947664	----------A------------	-	---I-----S-------------D---
	Sutcliffiella cohnii	WP_066420805	-------------D-T-----S-		---------A-A-K---------D---
	Bacillus safensis	BBP91156	----------------S---KS-		---V-----A---Q-IN--H-------
	Bacillus amyloliquefaciens	AHY24674	----------------S---KS-		---V-----A---Q-IN--H-------
	Bacillus subtilis	WP_032727487	----------------S---KS-		---V-----A---Q-IN--H-------
	Sutcliffiella halmapala	WP_078379565	-------------D-N-----S-		---------A-A---I---H-------
	Sutcliffiella horikoshii	WP_251433574	-------------D-T-----S-		---------A-A---IN------D---
	Bacillus tianshenii	WP_204416063	-------------D-N----KS-		-----S---A-A---I-----------
	Sutcliffiella deserti	WP_223702861	-------------D-N-----S-		-------N-A-A---I-----------
	Escherichia coli	MXF67979	----------------S---KS-		---V-----A---Q-IN--H-------
	Mesobacillus persicus	WP_090741189	--------------------KS-		---V-----S-A-T---------D---
	Bacillus massiliglaciei	WP_110927057	--------------------KS-		---V-----S-----IA----------
	Robertmurraya korlensis	WP_066050013	--------------------KS-		-------N-S-A-N-------------
	Neobacillus piezotolerans	WP_115453823	-------------D-T-----S-		-------L---A----A------D---
	Sporosarcina globispora	WP_053434284	---------------------S-		---S---N-S-A---------------
	Cytobacillus firmus	RBP94017	---------------------S-		---S---N-S-A---------------
	Peribacillus asahii	WP_119117468	--------------------KS-		---V-----S-Y-F-LA----------
Other Bacteria (5/ >100)	*Priestia megaterium*	TYR82303	--------------------KS-		---------S-S---I---H-------
	Bacillus cihuensis	WP_028392051	--------Y----D-T----KS-		---V---N-S-A-Q-I---------V-
	Peribacillus deserti	WP_101640548	--------------------KS-		---V-----S-S-N--N---R------
	Peribacillus huizhouensis	WP_182501748	--------Y----D-T----KS-		---V---N-S-A-Q-I---------V-
	Neobacillus soli	WP_066063894	--------------------KS-		-------S-S-A---------------
	Peribacillus muralis	WP_241577406	--------------------KS-		---V-----S-Y---IA------D---
	Metabacillus iocasae	WP_205182703	---------------------S-		---------S-S---IN--H-------
	Pontibacillus chungwhensis	WP_036785570	---------------T--T--S-		-----------M-Q-I---R---D---
	Peribacillus alkalitolerans	WP_163101826	---------------T-----S-		---V---N-S-A----N----------
	Peribacillus acanthi	WP_108669479	---------------T-----S-		---V---N-S-A----N----------
	Streptococcus pneumoniae	CJD46674	--------T----Y-------S-		-------N-S-S-R-LQ--K-------
	Mycobacteroides abscessus subs	SHP92392	------------------A-KS-		-------N-S-A-N-------------
	Cytobacillus oceanisediminis	WP_144545317	---------------------S-		---A---N-S-A-----------D---
	Pontibacillus halophilus	WP_026802095	---------------T-----S-		-----------M---I---RS------
	Halalkalibacterium ligniniphilum	WP_017728619	--Q-----T-S--T-------S-	-	---V-------S---ID--H----K--
	Alkalihalobacillus alcalophilus	WP_003324369	-----H--T-SR-S-H-----S-	-	---V-------L-R--D------DK--
	Halalkalibacterium halodurans	WP_010897410	--------T-S--L-N-----S-	-	--RV-------S---ID--H----K--
	Alkalihalobacillus pseudalcaliphilus	KMK75724	-----H--T-SR-S-N-----S-	-	---V-------L-R--D------DK--
	Alkalihalobacillus trypoxylicola	WP_061948240	--------T-SR-S-N-----S-	N	---V-------S-R--D------DK--

Figure S39: Partial sequence alignment of the bifunctional (p)ppGpp synthetase/guanosine-3’,5’-bis(diphosphate) 3’-pyrophosphohydrolase protein showing a 1 aa insert (highlighted) in a conserved region that is generally exclusively shared by species from the family *Anoxybacillaceae.* This CSI is also absent from most other bacteria. A few exceptions in distantly related species are present.
